# G Protein-Coupled Oestrogen Receptor Actions Targeting the Hallmarks of Cancer in Human Prostate Cells: From Cell Fate to Metabolic Reprogramming

**DOI:** 10.3390/cancers18071137

**Published:** 2026-04-01

**Authors:** Marília I. Figueira, Henrique J. Cardoso, Lara R. S. Fonseca, Tiago M. A. Carvalho, Sara Correia, Patrícia Arinto, Rui Henrique, Adriana O. Santos, Cláudio J. Maia, Sílvia Socorro

**Affiliations:** 1 RISE-Health, Department of Medical Sciences, Faculty of Health Sciences, University of Beira Interior, 6200-506 Covilhã, Portugal; henrique.cardoso@ipface.pt (H.J.C.); tiago.carvalho@ubi.pt (T.M.A.C.); scorreia@fcsaude.ubi.pt (S.C.); asantos@fcsaude.ubi.pt (A.O.S.); cmaia@fcsaude.ubi.pt (C.J.M.); 2 RISE-Health, Department of Chemistry, Faculty of Sciences, University of Beira Interior, 6200-506 Covilhã, Portugal; lara.fonseca@ubi.pt; 3 Cancer Genetics Group, IPO Porto Research Center (CI-IPOP)/RISE@CI-IPOP (Health Research Network), Portuguese Oncology Institute of Porto (IPO Porto)/Porto Comprehensive Cancer Center, 4200-072 Porto, Portugal; i12821@ipoporto.min-saude.pt; 4 Department of Laboratory Genetics, Portuguese Oncology Institute of Porto (IPO Porto)/Porto Comprehensive Cancer Center, 4200-072 Porto, Portugal; 5 Department of Pathology and Cancer Biology and Epigenetics Group—Research Center, Portuguese Oncology Institute of Porto/Porto Comprehensive Cancer Center Raquel Seruca (Porto.CCC), 4200-072 Porto, Portugal; rmhenrique@icbas.up.pt; 6 Department of Pathology and Molecular Immunology, ICBAS—School of Medicine and Biomedical Sciences, University of Porto, 4050-313 Porto, Portugal

**Keywords:** prostate cancer (PCa), G protein-coupled oestrogen receptor (GPER), proliferation, apoptosis, migration and invasion, metabolism, oxidative stress

## Abstract

**Background/Objectives:** The G protein-coupled oestrogen receptor (GPER) has anti-tumorigenic effects in several human cancers. However, its role in prostate cancer (PCa) remains incompletely defined. The present study investigated GPER’s role in targeting the hallmarks of PCa. **Methods:** Tissue microarrays were used to analyse GPER immunoexpression in PCa samples. Non-neoplastic (PNT1A) and neoplastic (LNCaP, DU145 and PC3) prostate cells were treated with the GPER-specific agonist, G1. Cell viability, proliferation, cell cycle, apoptosis, migration and invasion were evaluated. Glucose consumption, lactate production, lactate dehydrogenase activity and oxidative status were determined spectrophotometrically. **Results:** GPER immunoreactivity was higher in PCa than in benign prostatic hyperplasia and inversely correlated with PSA serum levels. G1 modulated GPER subcellular location in prostate cells, being detected at the cell membrane, endoplasmic reticulum, and residually in the nucleus. GPER activation decreased cell viability and proliferation, induced cell cycle arrest at G2/M phase, and increased PCa cells apoptosis. Additionally, GPER activation inhibited the migration and invasion of DU145 cells, and long-term exposure to G1 reduced epithelial–mesenchymal transition, an effect not observed in PC3 cells, indicating the importance of cell-specific contexts. Our results also showed that G1 treatment modulated the metabolic profile of PCa cells, changing glucose, amino acids and lipid metabolism. Finally, G1 increased oxidative stress in PCa cells. **Conclusions:** Overall, this study demonstrated that GPER activation affects a broad range of PCa hallmarks. These findings support an anti-cancer role for GPER in PCa and encourage further exploration of its action in regulating metabolism and as a therapeutic target.

## Simple Summary

The incidence of prostate cancer (PCa) continues to rise worldwide. As a hormone-dependent malignancy, PCa is strongly regulated by androgens and oestrogens. The G protein-coupled oestrogen receptor (GPER) is a membrane-associated oestrogen receptor, responsible for rapid non-genomic signalling, and has been associated with anti-tumorigenic effects in several cancers. However, its role in PCa remains unclear. Here, we investigated the impact of GPER in targeting multiple hallmarks of PCa. GPER activation by G1 reduced tumour cell growth and progression, supporting its anti-cancer role in this human neoplasia.

## 1. Introduction

Prostate cancer (PCa) is the second most frequently diagnosed malignancy in men worldwide, and its incidence continues to increase [1,2]. Despite the continuous advances in clinical management, significant challenges remain, particularly in overcoming resistance to androgen deprivation therapy [3,4].

PCa is a hormone-dependent cancer primarily driven by androgens, which play a preponderant role in the onset and progression of the disease, being associated with increased cell proliferation rates [5] and reduced apoptosis [6]. Also, it is widely accepted that PCa is sensitive to oestrogen, being hormones historically used for treatment before being discontinued due to adverse effects [7,8,9,10,11,12].

The G protein-coupled oestrogen receptor (GPER) is a membrane-bound oestrogen receptor (ER) that mediates rapid, non-genomic effects of oestrogens [13] by mobilising intracellular calcium and activating the phosphatidylinositol 3-kinase (PI3K)/AKT, mitogen-activated protein kinase (MAPK), Src kinase and AmpC pathways [14].

A substantial amount of evidence from different human cancers supports an anti-tumorigenic role for GPER [15,16,17,18,19,20], including inhibition of proliferation, migration, invasion and epithelial–mesenchymal transition (EMT), as well as induction of cell cycle arrest and apoptosis [16,19,21,22,23,24,25]. Some studies have reported the anti-cancer role of GPER in PCa, indicating that it reduces cell proliferation and tumour growth by arresting the cell cycle and increasing apoptosis [26,27,28]. For this reason, the GPER-specific agonist, G1 (GPR30-specific compound 1) [29], a substituted dihydroquinoline, with a tetrahydro-3H-cyclopenta[c]quinoline core structure [29], was investigated considering its potential utility in PCa treatment. Indeed, it has been shown that G1 inhibited the growth of castrate-resistant PCa cells but not of androgen-responsive cells, which supports GPER as a therapeutic target in castrate-resistant PCa (CRPC). However, other studies reported pro-carcinogenic actions of GPER in the prostate, inducing cell proliferation and tumour growth, restricting apoptosis and enhancing tumour progression [30,31,32]. Also, discrepancies exist concerning the expression pattern of GPER in the human prostate. Rago et al. [33] characterised GPER expression in benign and neoplastic tissues, which was inversely related to neoplastic cell differentiation [33]. Contrastingly, Lam et al. reported that GPER expression is higher in metastatic CRPC than in primary PCa [28]. These inconsistencies concerning GPER actions and expression strongly limit the perspective of targeting GPER in PCa therapy. Another bottleneck is that most studies have focused on the classical hallmarks of cancer (i.e., sustaining proliferative signalling and resisting cell death), rather than considering the new cancer hallmarks, such as metabolic reprogramming [34]. Furthermore, as GPER has been identified as a metabolic regulator [35], it is of utmost importance to investigate the role of GPER agonists in cancer cell metabolism.

The present study aimed to comprehensively characterise the impact of GPER activation on a broad range of cancer hallmarks in human prostate cells. G1 effects in PCa cell proliferation, apoptosis, migration, invasion, EMT, oxidative stress, and glucose, amino acids and lipid metabolism were investigated. Also, the GPER expression pattern in non-neoplastic and neoplastic human prostate and its subcellular localisation in human prostate cells were evaluated.

## 2. Materials and Methods

### 2.1. GPER Immunohistochemistry and Staining Scores

Tissue microarrays (TMAs) containing cases of benign prostatic hyperplasia (BPH, 34) and prostate adenocarcinoma (45), including non-neoplastic adjacent tissue and prostatic intraepithelial neoplasia (PIN) lesions (9), were obtained from the archive of the Pathology Service of “Hospital Geral de Santo António”, Oporto, including patients subjected to radical prostatectomy between 2010 and 2011 who did not receive preoperative chemotherapy or any other type of treatment.

TMA paraffin-blocks were cut into 2–4 μm sections, which were processed for immunohistochemical analysis. TMAs sections were deparaffinised in xylol and rehydrated through a series of graded ethanol solutions. Heat-induced antigen retrieval was performed in citrate buffer (10 mM, pH 6.0) for 20 min. Then, sections were permeabilised with 1% Triton X-100 for 15 min at room temperature. The activity of endogenous peroxidases was blocked with 3% hydrogen peroxide for 10 min. Phosphate-buffered saline (PBS) containing 1% bovine serum albumin (PBA) and 0.3 M glycine was used to avoid unspecific staining. Sections were incubated overnight at 4 °C with rabbit anti-GPER (1:50, (K-19)-R: sc-48524-R; Santa Cruz Biotechnology (SCBT), Dallas, TX, USA) primary antibody diluted in PBA, followed by 1 h incubation at room temperature with the goat anti-rabbit IgG biotinylated antibody (diluted 1:200 in PBA). Antibody binding was detected with the ExtrAvidin-Peroxidase reagent (1:20), min and 3,3-diaminobenzidine hydrochloride (Sigma-Aldrich, St. Louis, MO, USA) as a chromogen. TMAs sections were counterstained with Mayer’s haematoxylin (Bio-Optica, Milano, Italy), dehydrated, cleared and mounted with Entellan (Coverquick 2000, BDH Prolabo Chemicals, Fontenay-sous-Bois, France). Negative controls were performed by omission of the primary antibody.

The TMA examination and immunoreactivity scoring were performed by an experienced pathologist. GPER immunoreactivity scores were determined based on the intensity of staining and classified as “1” (low intensity) or “2” (high intensity). GPER immunoreactivity was correlated with clinical and histopathological patient data, namely age, histological diagnosis, total serum PSA levels, percentage of free PSA, Gleason scores, tumour node and metastasis (TNM) staging, and the presence of bone metastasis.

### 2.2. Cell Lines and Treatments

PNT1A (non-neoplastic) and LNCaP, DU145 and PC3 (neoplastic) cell lines were obtained from the European Collection of Cell Cultures (ECACC, Salisbury, UK). These human PCa cell lines correspond to distinct metastatic stages of progression and aggressiveness [36,37,38,39]. LNCaP cells are androgen-sensitive whereas DU145 and PC3 cells are androgen-insensitive and model the CRPC stage. Cells were cultured in RPMI 1640 medium (Sigma-Aldrich) supplemented with 10% foetal bovine serum (FBS) (Sigma-Aldrich) and 1% penicillin/streptomycin (Sigma-Aldrich) and maintained at 37 °C in an equilibrated atmosphere with 5% CO_2_. Upon reaching 60% confluence, cells were maintained in phenol red-free RPMI 1640 medium (Sigma-Aldrich) containing 5% charcoal-stripped FBS (CS-FBS) (Sigma-Aldrich), for an additional 24 h [40]. Subsequently, cells were treated with increasing concentrations of the GPER agonist G1 (Tocris Bioscience, Madrid, Spain), or vehicle for 24 h. The tested concentration range, from 0.001 to 100 μM, was selected based on previous in vitro studies investigating GPER activation, which showed that micromolar concentrations are commonly required to elicit measurable anti-proliferative effects [26]. For long-term experiments, cells were exposed to 1 µM G1 under the same conditions for 28 days.

Alternatively, PNT1A, LNCaP, DU145 and PC3 cells were treated with a normo-physiological concentration (10 nM) of 5α-dihydrotestosterone (DHT, Sigma-Aldrich) [41,42] or vehicle for 24 and 48 h.

### 2.3. Protein Extraction

Non-neoplastic and neoplastic cells were lysed in an appropriate volume of radioimmunoprecipitation assay buffer (RIPA) as described previously. Total protein concentration was subsequently determined using the BCA assay (Thermo Fisher, Rockford, IL, USA).

### 2.4. Western Blot (WB)

Total proteins were separated by SDS-PAGE using 12.5%, 10%, 7.5% or 7% polyacrylamide gels, transferred onto PVDF membranes (Bio-Rad, Hercules, CA, USA), and membranes were blocked as described in [40], prior to incubation overnight at 4 °C with the appropriate primary antibodies (Supplementary Table S1). Thereafter, incubation proceeded with the secondary antibodies: goat anti-rabbit IgG-HRP (1:40,000, sc:2004; SCBT) or goat anti-mouse IgG-HRP (1:40,000, sc:2005; SCBT). For WB analyses of G1-treated samples, a mouse monoclonal anti-α-tubulin antibody (1:10,000, Sigma-Aldrich) was used for protein loading control, whereas a mouse monoclonal anti-β-actin antibody (1:10,000, Sigma-Aldrich) served for the protein loading control of DHT-treated samples.

Immunoreactive bands were detected by incubating membranes with ECL substrate (Bio-Rad) for 5 min and using the ChemiDoc™ MP Imaging System (Bio-Rad). Band densities were quantified and normalised to the respective α-tubulin or β-actin signal as described in [40].

### 2.5. Fluorescent Immunocytochemistry

PNT1A, LNCaP, DU145 and PC3 cells were seeded in 24-well plates containing sterile glass coverslips and allowed to adhere overnight. Subsequently, cells were treated with G1 as described. After 24 h of treatment, cells were fixed and permeabilized as described previously [43]. Non-specific binding sites were blocked with PBS containing 0.1% Tween-20 (PBS-T) and 20% FBS, and cells were then incubated for 1 h at room temperature with the following primary antibodies: rabbit anti-Ki-67 (1:50, ab16667; Abcam, Cambridge, UK), rabbit anti-GPER (1:50, ab39742; Abcam) or rabbit anti-calnexin (1:50, H-70: sc-11397; SCBT). Alexa Fluor 488 goat anti-rabbit IgG or Alexa Fluor 546 goat anti-rabbit IgG were used as secondary antibodies (Invitrogen, Thermo Fisher, Carlsbad, CA, USA). For double immunolabelling experiments, an additional 1 h blocking step was included between calnexin staining and the subsequent GPER staining. Omission of the primary antibodies allowed us to confirm staining specificity. Cell nuclei were stained with Hoechst 33342 (5 µg/mL, Invitrogen, Thermo Fisher) for 10 min and plasma membrane with wheat germ agglutinin (WGA, 5 µg/mL, Invitrogen, Thermo Fisher) for 15 min prior to permeabilization. Lamellae were mounted using Dako fluorescent mounting medium (Dako, Glostrup, Denmark). Images were captured using the Axio Imager A1 microscope (Carl Zeiss, Göttingen, Germany) or a Zeiss LSM 710 laser scanning confocal microscope (Carl Zeiss).

### 2.6. Cell Viability Assay

Prostatic cell lines (PNT1A, LNCaP, DU145 and PC3) were plated in 96-well plates and allowed to adhere prior to treatment with increasing concentrations of G1 (0.001 µM–100 µM) for 24 h. Cell viability was assessed using the colorimetric MTT assay, as reported in [40]. Absorbance of the resultant purple-coloured solution was measured at 570 nm using the xMark™ Spectrophotometer (Bio-Rad). The absorbance value is directly proportional to the number of metabolically active, viable cells in each experimental condition.

### 2.7. Cell Proliferation Analysis

LNCaP, DU145 and PC3 cell counting was performed by nuclear staining with Hoechst 33342 (5 µg/mL) for 10 min. The number of cells in control and G1-treated groups (1 µM) was determined in a total of 10 400× magnification fields per slide. Images were acquired using the Axio Imager A1 microscope (Carl Zeiss).

The proliferation index was assessed by fluorescent immunocytochemistry analysis of the nuclear cell proliferation marker Ki-67 [44]. The percentage of Ki-67-positive cells out of the total number of Hoechst-stained nuclei was determined in 10 randomly selected 400× magnification fields in each lamella.

### 2.8. Flow Cytometry

Propidium iodide (PI) staining and flow cytometry were used to evaluate cell cycle distribution and the occurrence of apoptotic (sub-G1) nuclei. Briefly, LNCaP (1,000,000 cells/t-flask 25 cm^2^), DU145 (300,000 cells/t-flask 25 cm^2^) and PC3 (200,000 cells/t-flask 25 cm^2^) cells were treated with G1 as described (2.2). Twenty-four hours after stimulation, adherent cells were harvested by trypsin treatment and pooled with the cells collected in the supernatant medium. The cell suspension was kept on ice, pelleted, and then resuspended in 950 µL PBS before being fixed with −20 °C cold 70% ethanol in ice for 30 min and used. Fixed cells were centrifuged, and the ethanol solution was removed. For DNA fragments extraction, 800 µL of PBS and 1 mL of DNA extraction buffer (0.2 M sodium phosphate, 0.1% Triton X-100, pH 7.8) were added to the pellet of cells. After 5 min incubation, cells were centrifuged, the supernatant discarded, and the pellet resuspended in the staining solution (20 μg/mL PI and 0.2 mg/mL of RNase-free DNase). At least 20,000 events were acquired per sample using a FACSCalibur flow cytometer (BD Biosciences, Paramus, NJ, USA), with a 488 nm laser. Debris and residual necrotic cells were excluded, using appropriate gating strategies, based on lower diameter (Forward scatter; FSC) and reduced PI fluorescence (Fluorescence channel 3; FL3), compared to hypodiploid apoptotic cells. FlowJo software (version 10.8.1) was used for all data analyses. Cell cycle analysis was performed in asynchronously growing cells to evaluate the overall effect of G1 under standard culture conditions, reflecting the heterogeneous proliferative state typical of tumour cell populations.

### 2.9. Caspase-3-like Activity Assay

Caspase-3-like activity was evaluated as describe in [40]. Briefly, 5 µL of total protein extracted from PNT1A, LNCaP, DU145 and PC3 cells were incubated overnight at 37 °C with 85 µL of assay buffer (20 mM HEPES, pH 7.4, 2 mM EDTA, 0.1% CHAPS, 5 mM DTT) and 200 µM of caspase-3 specific substrate Ac-DEVD-pNA (Sigma-Aldrich). The formation of the yellow chromophore pNA (Sigma-Aldrich) was quantified by measuring absorbance at 405 nm using the xMark™ Spectrophotometer (Bio-Rad). The concentration of released pNA was determined by interpolation from a standard curve, and the estimated caspase-3-like activity values were normalised to the total protein content (µg) of each sample.

### 2.10. Terminal Deoxynucleotidyl Transferase dUTP Nick-End Labelling (TUNEL)

TUNEL assay was carried out using the In Situ Cell Death Detection Kit, TMR red (Roche, Basel, Switzerland) according to the manufacturer’s guidelines. In sum, cells were washed using PBS, fixed in 4% PFA, and permeabilised using 0.1% Triton X-100 in 0.1% sodium citrate for 2 min on ice. Subsequently, cells were incubated with the enzyme solution (equilibrated in label solution) for 1 h at 37 °C in a humidified chamber, protected from light. After that, cells were rinsed with PBS and nuclei were counterstained with Hoechst 33342 (5 µg/mL, 10 min). After washing, cells were mounted using Dako fluorescent mounting medium (Dako). Images were captured using a Zeiss LSM 710 laser scanning confocal microscope (Carl Zeiss). The apoptotic index was calculated as the percentage of TUNEL-positive cells relative to the total number of Hoechst-stained nuclei, based on the analysis of 10 randomly selected fields per lamella, at 400× magnification.

### 2.11. Transwell Migration and Invasion Assays

Evaluation of migration and invasion capabilities of prostate cells was carried out in 24-well plates using uncoated (SPL Life Sciences, Pocheon-si, Republic of Korea) or Matrigel-coated (Corning, New York, NY, USA) chambers, respectively, with 8 µm pore-size membranes. Briefly, one day before the assays, cells were transferred to phenol red-free RPMI 1640 culture medium supplemented with 5% CS-FBS. Cell suspensions were prepared in serum-free medium, and 1–3 × 10^5^ cells were placed in the upper transwell chamber. Culture medium with 10% FBS (750 µL) was added to the lower chamber as the chemoattractant.

Cells were maintained in an incubator at 37 °C with 5% CO_2_ for 24 h. After that, cells in the top chamber were removed, using a cotton bud moistened in serum-free medium. Migrated and invaded cells at the bottom of membranes were fixed with 4% PFA for 10 min, and the nuclei were stained with Hoechst 33342 (5 µg/mL). The number of migrated and invaded cells was counted in five randomly selected 400× magnification fields using the Axio Imager A1 microscope (Carl Zeiss). Representative images of migrated or invaded cells were acquired using the Zeiss LSM 710 laser scanning confocal microscope (Carl Zeiss).

### 2.12. Quantification of Glucose and Lactate

Glucose and lactate levels in the culture medium of control and G1-treated cells were determined according to the manufacturer’s guidelines (Spinreact, Girona, Spain) [40]. Glucose consumption and lactate production following 24 h of treatment were calculated by comparing metabolites concentrations at the end of treatment period with their corresponding baseline values measured at 0 h. The resulting absorbance was recorded (505 nm) using the xMark™ microplate Spectrophotometer (Bio-Rad).

### 2.13. Lactate Dehydrogenase (LDH) Enzymatic Activity Assay

A commercial assay kit (Spinreact) was used to determine LDH activity considering the rate of NADH consumption in 5 min [40]. The decrease in absorbance at 340 nm was recorded immediately (initial reading) and subsequently measured at 1 min intervals, using the xMark™ microplate Spectrophotometer (Bio-Rad). LDH activity was normalised to the total amount (μg) of protein in each sample being expressed as U/L/ug of protein.

### 2.14. Glutathione Peroxidase (GPX) Activity Assay

GPX activity was determined using a commercial kit (703102; Cayman Chemical, Ann Arbor, MI, USA) according to the manufacturer’s protocol. Enzymatic activity was measured indirectly through a coupled reaction involving glutathione reductase. During the reduction of hydroperoxide by GPX, oxidised glutathione is recycled to its reduced form by glutathione reductase in the presence of NADPH. This reaction leads to the oxidation of NADPH to NADP^+^, resulting in a decrease in absorbance at 340 nm. This reduction is proportional to the GPX activity if the GPX activity is rate-limiting. In brief, 10 µL of protein extract were pipetted into a 96-well microplate (containing 25 µL of assay buffer, 25 µL of co-substrate mixture and 25 µL of NADPH), and the decrease in the A340 was read at 1 min intervals for 5 min in the xMark™ Spectrophotometer (Bio-Rad). The rate of ΔA340 per minute was used to calculate GPX activity. Results were expressed as nmol/min/mL/µg total protein.

### 2.15. Superoxide Dismutase (SOD) Activity Assay

SOD activity was assessed using the commercial kit SOD Assay Kit (19160-1KT-F; Sigma-Aldrich) following the manufacturer’s instructions. Briefly, this kit uses the highly water-soluble tetrazolium salt, WST-1, that produces a water-soluble formazan dye upon reduction with the superoxide anion. The rate of formazan reduction is linearly related to the activity of xanthine oxidase and inhibited by SOD. Thus, the percentage of reaction inhibition rate indicates the amount of SOD activity, which was monitored by measurement of absorbance at 450 nm in the xMark™ Spectrophotometer (Bio-Rad). The amount of protein extract used was 5 µL into a 96-well microplate (containing 100 µL of WST working solution plus 5 µL of enzyme working solution). The reduction reaction occurred at 37 °C for 20 min. Results were expressed as a proportion of activity (percentage of inhibition) relative to the control group.

### 2.16. Total Oxidant Status (TOS) Analysis

TOS was determined using a commercial kit (RL0024, Rel Assay Diagnostics, Gaziantep, Turkey). The assay is based on the oxidation of the ferrous ion-chelator complex to ferric ions by oxidant species present in the sample. The resulting ferric ions react with a chromogen substrate in an acidic medium, resulting in a coloured complex. The colour intensity determined spectrophotometrically is proportional to the total amount of oxidant species in the sample. Briefly, 7.5 µL of sample or standard (10 µmol/L H_2_O_2_) were pipetted into a 96-well microplate and mixed with the provided buffer solution (25 mM H_2_SO_4_). The absorbance at 530 nm was read at the initial time point and after 5 min of incubation with substrate solution (25 mM H_2_SO_4_, 5 mM ferrous ion and 10 nM O-dianisidine) at 37 °C in the xMark™ Spectrophotometer (Bio-Rad). Results were expressed in μmol H_2_O_2_ Equiv./L.

### 2.17. Statistical Analysis

In the case of GPER expression in TMAs, statistical analysis was performed using IBM SPSS (v23.0 IBM Corp, New York, NY, USA). Pearson chi-square and Fisher exact tests were used to determine the correlation between GPER immunoreactivity and individual variables of clinical–pathological data.

For the in vitro experiments, statistical analyses were performed using an unpaired T-test (with appropriate correction when required) or one-way ANOVA, followed by a suitable post hoc test, using GraphPad Prism v6.01 (GraphPad Software, Inc., La Jolla, CA, USA).

Differences were considered statistically significant at *p* < 0.05. Data are presented as mean ± standard error of the mean (SEM).

## 3. Results

### 3.1. GPER Immunoreactivity Is Higher in Human PCa and Correlated with Total PSA Levels

As previous studies have produced discrepant results regarding GPER expression in benign and neoplastic human prostate tissues [26,31,32,33], and as a basis for investigating GPER-mediated modulation of PCa cell behaviour in vitro, we first assessed receptor expression pattern in human PCa samples using TMAs. GPER immunoreactivity was categorised as low (score 1) or high (score 2) scores according to the intensity of staining. GPER immunoexpression levels were significantly higher in PCa than in BPH (*p* < 0.001, Table 1). However, no significant differences in GPER immunoreactivity were observed between BPH and PIN (*p* = 0.271) or between PIN and adenocarcinoma (*p* = 0.412). Notably, 40% of the adenocarcinoma specimens exhibited high GPER immunoreactivity, compared with only 22.22% of PIN lesions, while all BPH samples displayed low GPER immunoreactivity (Table 1).

**Figure 1:**
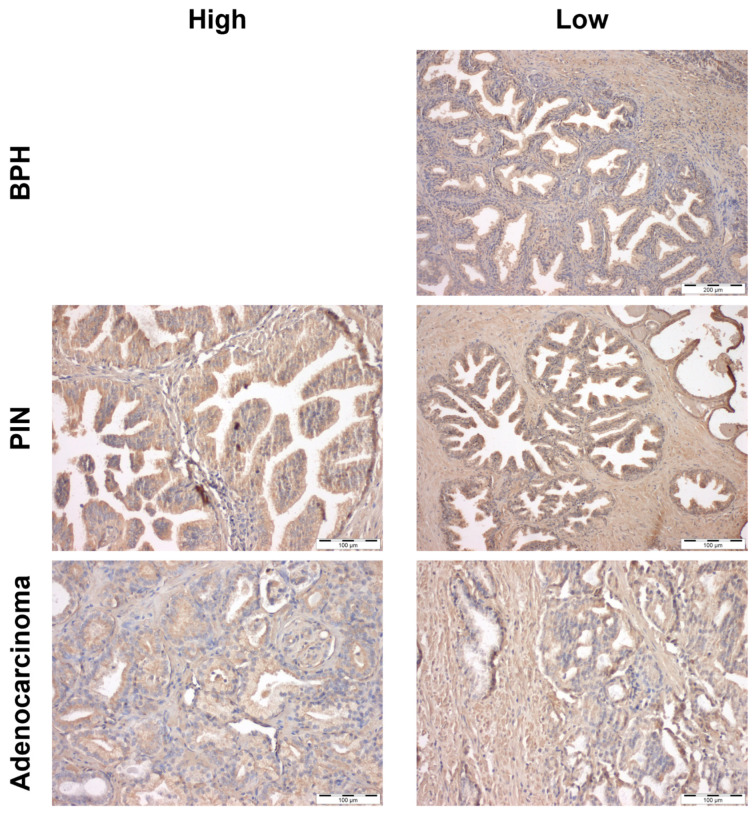
Representative images of GPER high vs. low immunoreactivity in human benign prostatic hyperplasia (BPH), prostatic intraepithelial neoplasia (PIN) lesions and prostate adenocarcinoma. Haematoxylin–eosin counterstaining magnification 40×. Scale bar 200 μm for BPH and 100 μm for PIN and adenocarcinoma.

Representative immunohistochemical images showing high GPER immunoreactivity in BPH, PIN lesions and adenocarcinoma of prostate are presented in Figure 1. BPH sections exhibited normal tissue architecture, characterised by branching glands surrounded by stroma. IN BPH samples, GPER immunoreactivity was detected in both epithelial and stromal cells (Figure 1). In PIN lesions, GPER immunoreactivity was preserved in the stromal compartment and luminal epithelial cells (Figure 1). In PCa samples, tumour cells displayed a distinct architectural arrangement, accompanied by strong GPER immunoreactivity (Figure 1).

The association between GPER immunoreactivity and clinical and histopathological data (Table 1), including age, total PSA, percentage of free PSA, Gleason score, pathologic stage and the presence of bone metastasis, was evaluated. GPER immunoreactivity was negatively associated with the total PSA levels (*p* < 0.05, Table 1). Notably, 80% of cases with PSA levels ≤ 4 ng/mL exhibited high GPER immunoreactivity, compared with only 26.32% of cases with PSA levels > 4 ng/mL (Table 1).

### 3.2. GPER Expression Pattern and Subcellular Localisation in Human Prostate Cells

**Figure 2:**
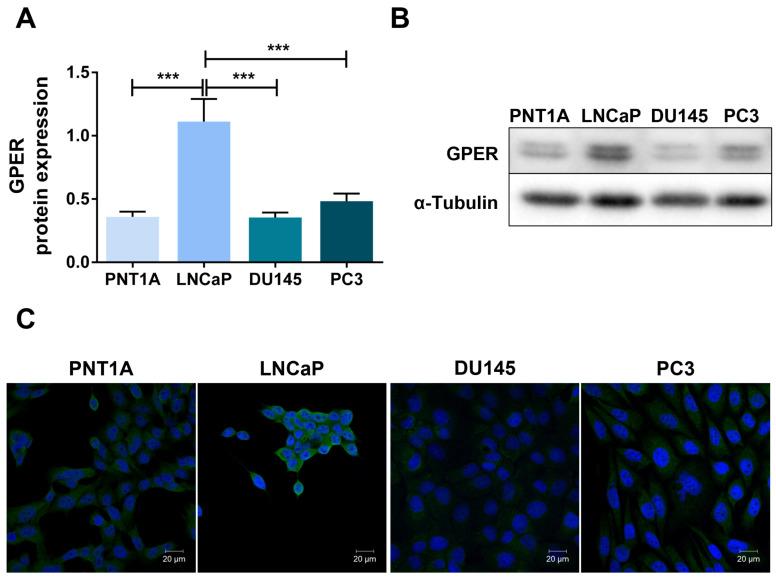
GPER expression in non-neoplastic PNT1A and neoplastic LNCaP, DU145 and PC3 human prostate cells. (**A**) Protein expression determined by WB analysis after normalisation with α-tubulin. Error bars indicate mean ± S.E.M. (n = 3) *** *p* < 0.001. (**B**) Representative immunoblots. (**C**) Representative images of GPER subcellular localisation obtained with a Zeiss LSM 710 laser scanning confocal microscope under 400× magnification. Nuclei are stained blue and GPER green. Original western blots are presented in File S1.

After confirming GPER expression in human PCa samples, and prior to conducting the in vitro studies aimed at investigating its role in modulating PCa cell behaviour, we characterised receptor expression in the prostate cell line models used in this study. Non-neoplastic (PNT1A) and neoplastic (LNCaP, DU145 and PC3) cell lines representing different stages of PCa were included. LNCaP cells are androgen-sensitive, whereas DU145 and PC3 cells are CRPC models that mimic more aggressive stages of the disease. LNCaP cells exhibited the highest GPER protein expression. GPER levels were significantly higher in LNCaP cells compared with both non-neoplastic PNT1A cells and neoplastic DU145 and PC3 cells (Figure 2A,B). In contrast, GPER expression in DU145 and PC3 cells was comparable to that observed in PNT1A (Figure 2A,B). Immunofluorescence cytochemistry further confirmed GPER expression in PNT1A, LNCaP, DU145 and PC3 cells (Figure 2C), with higher green fluorescence intensity observed in LNCaP cells, consistent with the higher protein expression levels.

**Figure 3:**
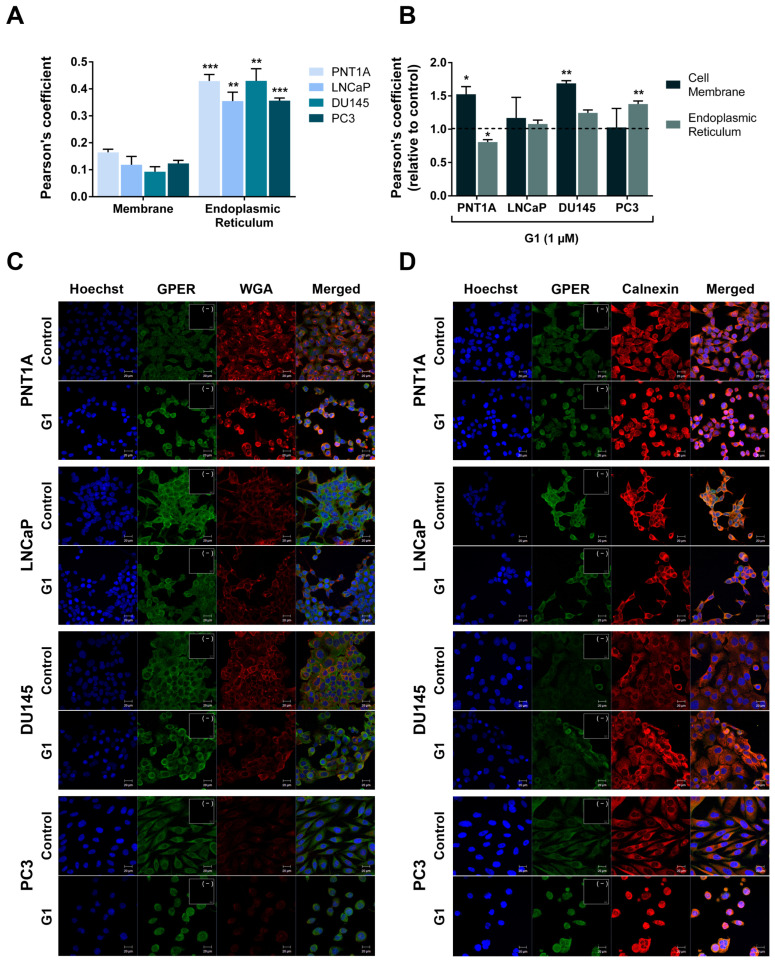
GPER subcellular localisation in non-neoplastic PNT1A and neoplastic LNCaP, DU145 and PC3 human prostate cells. (**A**) Pearson’s correlation coefficient for the GPER co-localisation with the cell membrane and endoplasmic reticulum. (**B**) Pearson’s correlation coefficient for the GPER co-localisation with the cell membrane and endoplasmic reticulum after GPER activation by G1 (1 µM). Results are expressed as fold-change relative to the control untreated group (0 μM G1, dashed line). Error bars indicate mean ± S.E.M. (n = 3) * *p* < 0.05 ** *p* < 0.01 *** *p* < 0.001. (**C**,**D**) Representative confocal microscopy images showing the co-localisation of GPER (green) with cell membrane (WGA in red), endoplasmic reticulum (calnexin in red) or nucleus (Hoechst in blue) in control and G1-treated (1 µM) cells. Images were obtained in the Zeiss LSM 710 laser scanning confocal microscope under 400× magnification. Negative controls obtained by omission of the primary antibody or WGA are provided as insert panels (−).

GPER has been reported to localise to the endoplasmic reticulum and the nucleus in different cell types [45,46]. This prompted us to investigate the GPER subcellular distribution in human prostate cells using fluorescent immunocytochemistry to assess its co-localisation with WGA, a specific marker of cell membrane, calnexin, an endoplasmic reticulum marker, and Hoechst, a nuclear staining dye. Immunofluorescence analysis revealed that GPER was predominantly localised to the endoplasmic reticulum in all prostate cells (average Pearson’s coefficient of 0.429, 0.355, 0.430 and 0.357 for PNT1A, LNCaP, DU145 and PC3 cells, respectively, Figure 3A,C). GPER was also detected at the cell membrane, although to a lesser extent (average Pearson’s coefficient of 0.165, 0.119, 0.092 and 0.123 for PNT1A, LNCaP, DU145 and PC3 cells, respectively; Figure 3A,D).

Residual GPER staining was observed in the nuclei of both non-neoplastic and neoplastic cells (Figure 3C,D).

GPER subcellular location has been reported to be modulated by oestrogen binding and receptor activation. Therefore, we tested whether G1 affected GPER distribution across cellular compartments. G1 treatment increased GPER localisation at the cell membrane in PNT1A and DU145 (1.53 ± 0.11 and 1.69 ± 0.04 fold-change relative to control, respectively), whereas no significant changes were observed in LNCaP and PC3 cells (Figure 3B,C). G1 also enhanced GPER localisation to the endoplasmic reticulum in PC3 cells (1.38 ± 0.04 fold-change relative to control, Figure 3B,D). In contrast, G1-treated PNT1A cells exhibited reduced location of GPER to the endoplasmic reticulum (0.81 ± 0.04 fold-change relative to control, Figure 3B,D). G1 treatment did not change GPER localisation to the endoplasmic reticulum localisation in LNCaP and DU145 cells (Figure 3B,D). Furthermore, no apparent alterations in nuclear GPER staining were observed in response to G1 treatment in any of the cell lines analysed (Figure 3C).

### 3.3. Androgens Modulated GPER Protein Expression Levels in Prostate Cells

**Figure 4:**
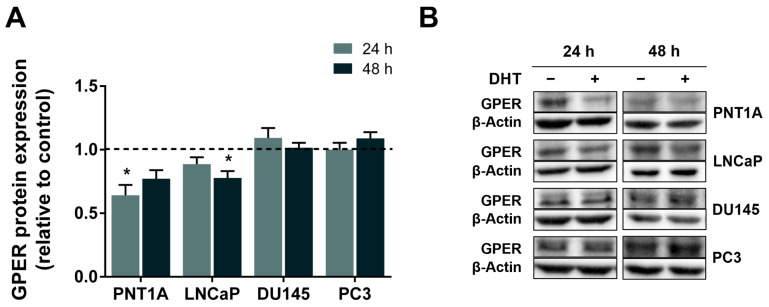
Effect of androgens in regulating GPER expression in LNCaP, DU145 and PC3 human PCa cells. (**A**) GPER protein expression after treatment with DHT (10 nM) for 24 or 48 h. Protein expression was determined by WB analysis after normalisation with β-actin. Results are expressed as fold-change relative to the control untreated group (0 nM DHT, dashed line). Error bars indicate mean ± S.E.M. (n = 5) * *p* < 0.05. (**B**) Representative immunoblots. Original western blots are presented in File S1.

The differential expression of GPER between CRPC and androgen-sensitive LNCaP cells suggests that it may be regulated by androgens. To investigate this possibility, non-neoplastic PNT1A and neoplastic LNCaP, DU145 and PC3 cells were treated with 10 nM DHT for 24 and 48 h, and GPER protein expression was analysed by WB. DHT treatment reduced GPER protein levels in PNT1A (0.64 ± 0.08 fold-change relative to control, 24 h) and, to a lesser extent, in LNCaP (0.78 ± 0.05 variation to control, 48 h) cells. No significant changes were observed in the DU145 and PC3 cells (Figure 4).

### 3.4. G1 Treatment Decreased the Viability and the Proliferative Behaviour of PCa Cells

**Figure 5:**
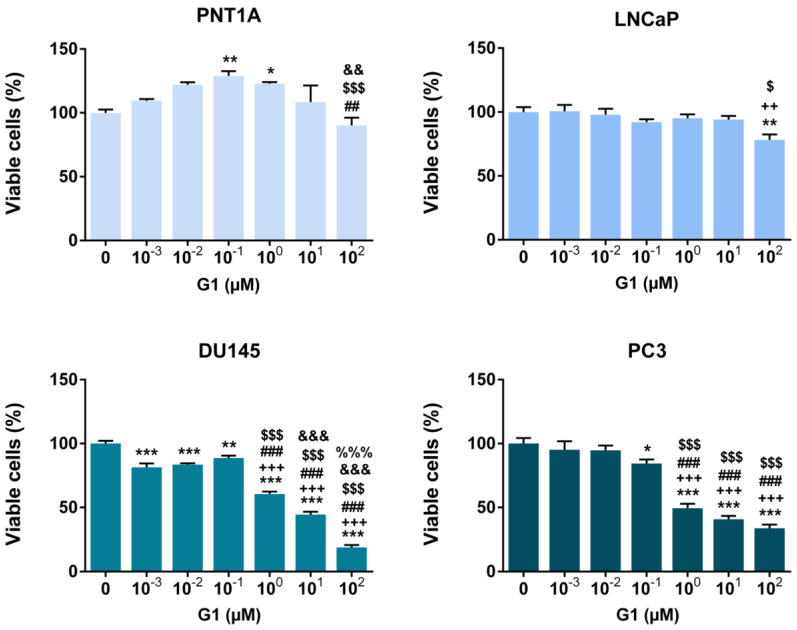
Viability of non-neoplastic PNT1A and neoplastic, LNCaP, DU145 and PC3 human prostate cells in response to G1. Cells were treated with a range of G1 concentrations (0 µM–100 µM) for 24 h. Results are expressed as % of control (0 µM G1). Error bars indicate mean ± S.E.M. (n = 5) * *p* < 0.05 ** *p* < 0.01 *** *p* < 0.001 relative to 0 µM; ^++^ *p* < 0.01 ^+++^ *p* < 0.001 relative to 0.001 µM; ^##^ *p* < 0.01 ^###^ *p* < 0.001 relative to 0.01 µM; ^$^ *p* < 0.05 ^$$$^ *p* < 0.001 relative to 0.1 µM; ^&&^ *p* < 0.01 ^&&&^ *p* < 0.001 relative to 1 µM; ^%%%^ *p* < 0.001 relative to 10 µM.

To begin evaluating the role of GPER in modulating prostate cells’ fate, PNT1A, LNCaP, DU145 and PC3 cells were exposed to a concentration range of GPER-specific agonist G1 for 24 h, and cell viability was analysed (Figure 5). The highest G1 concentration tested (100 µM) reduced cell viability by approximately 22, 66 and 81% relative to control in LNCaP, PC3 and DU145, respectively (Figure 5). In DU145 cells, G1 effects were concentration-dependent at concentrations above 0.1 µM (Figure 5).

Exposure to 100 µM G1 also reduced the viability of non-neoplastic PNT1A cells. However, lower concentrations (0.1 and 1 µM) significantly increased PNT1A cell viability compared with the control group (approximately 29 and 23% increase, respectively, Figure 5).

The IC50 values for G1 were approximately 32, 1.9 and 0.9 µM in LNCaP, DU145 and PC3 cells, respectively. An IC50 value could not be calculated for PNT1A.

A concentration of 1 µM G1 was selected for subsequent experiments, as it reduced DU145 and PC3 cell viability by approximately 50% and has been reported to exert biological effects in several cancer cell line models [27,47].

**Figure 6:**
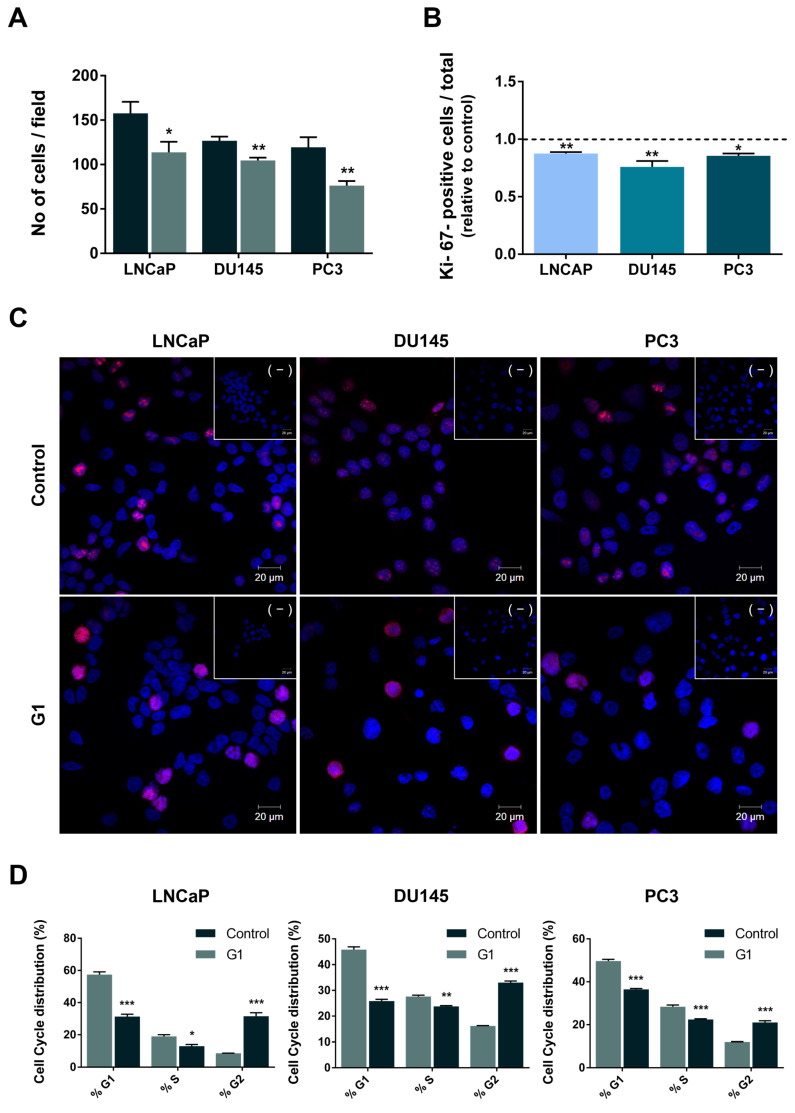

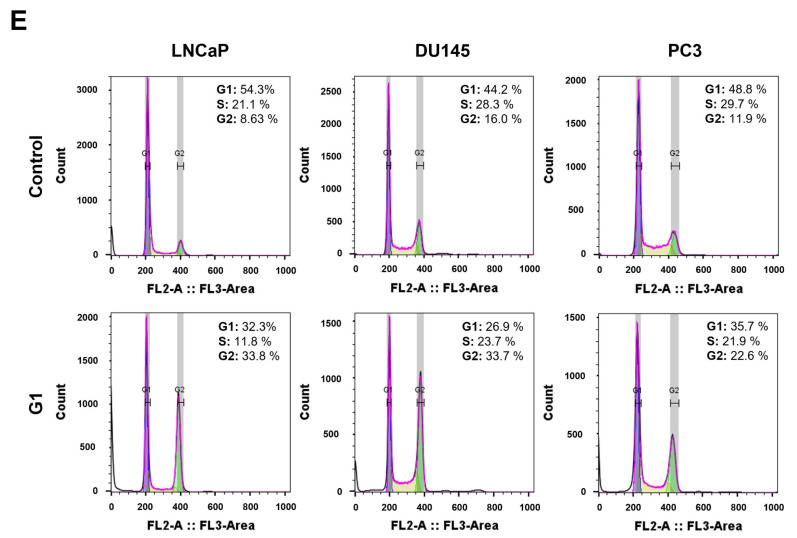
Effect of G1 on LNCaP, DU145 and PC3 human PCa cell fate. Cells were treated with 1 µM G1 for 24 h. (**A**) Cell number determined through counting Hoechst-stained nuclei. (**B**) Percentage of Ki67-positive cells relative to the total cell number (Hoechst-stained nuclei). A total of 10 400× magnification fields per slide was assessed. Results are expressed as fold-change relative to the control untreated group (0 µM G1, dashed line). Error bars indicate mean ± S.E.M. (n = 2) * *p* < 0.05 ** *p* < 0.01. (**C**) Representative confocal microscopy images showing Ki67 labelling in control and G1-treated cells. Images were obtained in the Zeiss LSM 710 laser scanning confocal microscope (Carl Zeiss, Göttingen, Germany) under 400× magnification. Nuclei are stained with Hoechst 33342 (blue) and Ki67-positive staining is red. Negative controls obtained by omission of the primary antibody are included as insert panels (−). (**D**) Percentage of LNCaP, DU145 and PC3 cells distributed in G1, S, and G2/M phases. The number of events was counted according to the DNA content by propidium iodide (PI) staining. Cell cycle analysis was processed in FlowJo. Error bars indicate mean ± S.E.M. (n = 3) * *p* < 0.05 ** *p* < 0.01 *** *p* < 0.001. (**E**) Representative flow cytometry histograms and respective percentage of cells.

Consistent with the reduction in cell viability, G1 exposure significantly decreased the number of PCa cells (Figure 6A). Accordingly, the proliferation index, determined by immunocytochemistry analysis of the Ki67 nuclear proliferation marker, was significantly reduced in G1-treated LNCaP, DU145 and PC3 cells (0.88 ± 0.01, 0.76 ± 0.05 and 0.86 ± 0.02 fold-change relative to control, Figure 6B,C). Representative images of immunofluorescence staining (Figure 6C) suggest higher Ki67 staining in some LNCaP, DU145 and PC3 cells after G1 treatment (Figure 6C).

Flow cytometry was performed to analyse cell cycle distribution in control and G1-treated cells. G1 stimulation significantly increased the percentage of PCa cells in the G2/M-phase by 23%, 17% and 9% in LNCaP, DU145 and PC3, respectively (Figure 6D,E).

**Figure 7:**
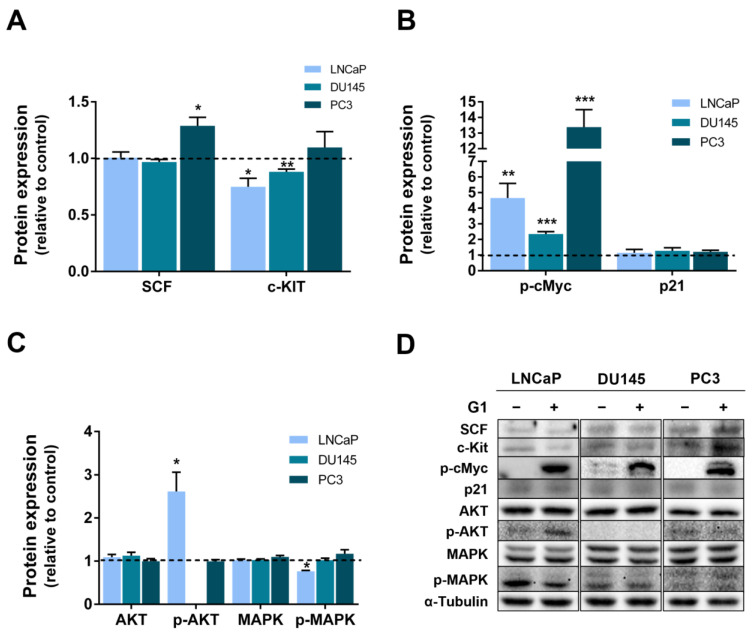
Effect of G1 on the expression of cell cycle regulators and intracellular signalling players in LNCaP, DU145 and PC3 human PCa cells. Cells were treated with 1 µM G1 for 24 h. Protein expression of (**A**) SCF ligand and receptor tyrosine kinase c-Kit, (**B**) p-cMyc and p21, and (**C**) AKT, p-AKT, MAPK and p-MAPK determined by WB analysis after normalisation with α-tubulin. Results are expressed as fold-change relative to the control untreated group (0 µM G1, dashed line). Error bars indicate mean ± S.E.M. (n = 5) * *p* < 0.05; ** *p* < 0.001, *** *p* < 0.01. (**D**) Representative immunoblots. Original western blots are presented in File S1.

The effects of G1 on PCa cell proliferation were complemented by analysing the expression of oncogenes and key regulators of cell cycle progression. We analysed the expression of the proliferation driver and oncogene receptor tyrosine kinase c-Kit and its specific ligand, the stem cell factor (SCF), as we previously showed that E_2_ downregulates c-Kit expression both in vitro and in vivo, thereby impacting proliferation and apoptosis [43]. G1 treatment reduced c-Kit expression in DU145 and LNCaP cells (0.88 ± 0.02 and 0.75 ± 0.08 fold-change relative to control, respectively, Figure 7A), whereas SCF expression remained unchanged. In PC3 cells, G1 treatment increased the expression of SCF (1.29 ± 0.08 fold-change relative to control, Figure 7A), with no significant change in c-Kit protein levels.

Regarding other cell cycle regulators and oncogenes, we focused on p21 and c-Myc, given their established relevance in PCa [48]. The phosphorylation of c-Myc regulates its stability, as it is a required step for ubiquitin-mediated degradation [49,50,51]. Therefore, we evaluated the expression of phosphorylated c-Myc (p-cMyc). Exposure to GPER agonist markedly increased p-cMyc expression in PCa cells (2.35 ± 0.15, 4.95 ± 0.94, and 13.38 ± 1.12 fold-change relative to control in DU145, LNCaP and PC3, respectively, Figure 7B). In contrast, p21 protein expression remained unchanged in all cell lines following G1 treatment (Figure 7B).

To further investigate the intracellular signalling pathways involved in the cellular response to G1, we evaluated the AKT and MAPK/ERK pathways (Figure 7C). G1 exposure increased p-AKT levels in LNCaP cells (2.62 ± 0.45 fold-change relative to control, Figure 7C), whereas p-MAPK levels were reduced (0.76 ± 0.02 fold-change relative to control, Figure 7C). No significant alterations in p-AKT and p-MAPK expression were observed in DU145 and PC3 cells following G1 treatment (Figure 7C,D).

### 3.5. GPER Activation Induced Apoptosis of PCa Cells

**Figure 8:**
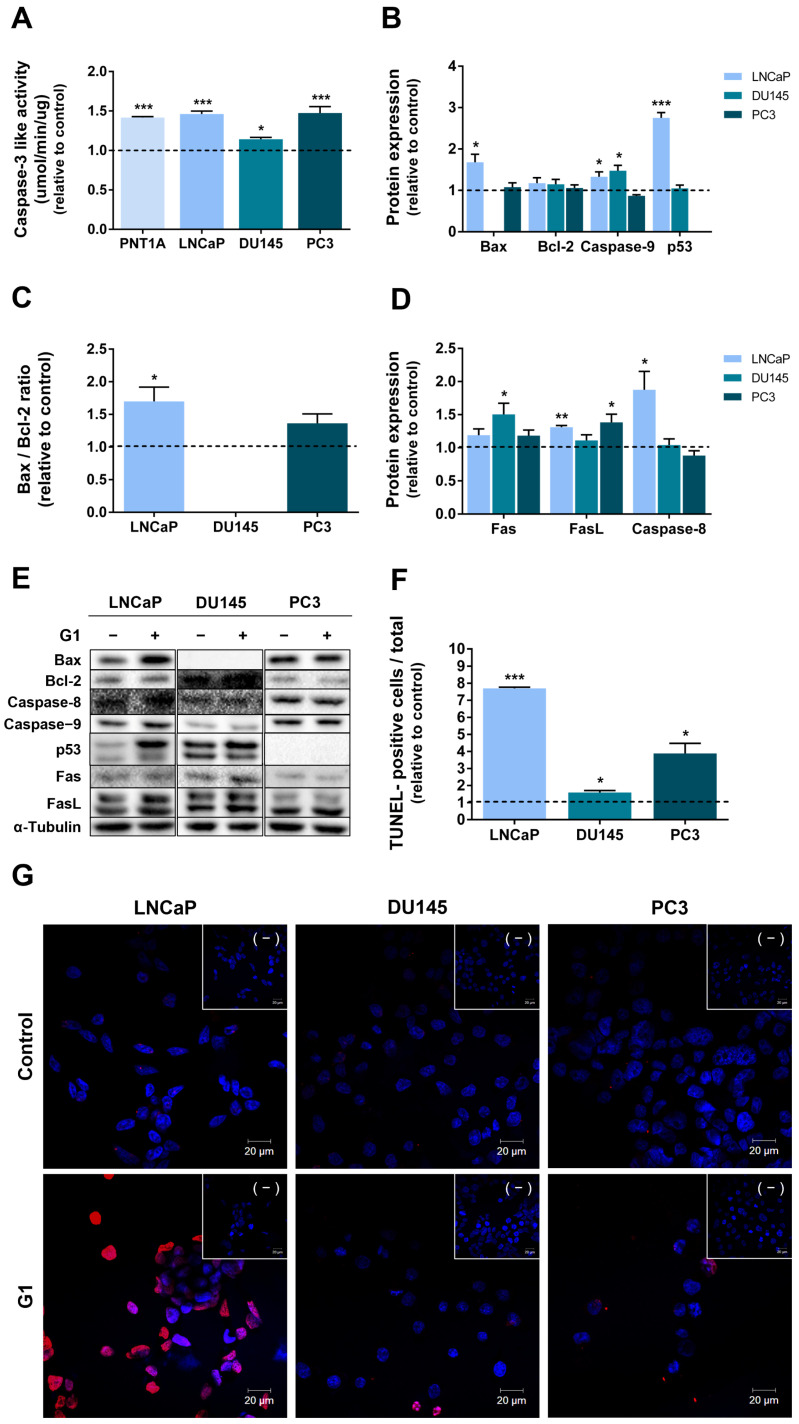
Effect of G1 in LNCaP, DU145 and PC3 human PCa cells apoptosis. Cells were treated with 1 µM G1 for 24 h. (**A**) Caspase-3-like activity determined spectrophotometrically. (**B**–**D**) Protein expression of apoptosis regulators of the (**B**,**C**) intrinsic and (**D**) extrinsic pathways of apoptosis determined by WB analysis after normalisation with α-tubulin. (**E**) Representative immunoblots. (**F**) Percentage of TUNEL-positive cells relative to the total cell number (Hoechst-stained nuclei). A total of 10 400× magnification fields per slide was assessed. Results (**A**–**D**,**F**) are expressed as fold-change relative to the control untreated group (0 µM G1, dashed line). Error bars indicate mean ± S.E.M. (n = 5) * *p* < 0.05 ** *p* < 0.01 *** *p* < 0.001. (**G**) Representative fluorescence microscopy images showing TUNEL in control and G1-treated cells. Images were acquired using a Zeiss LSM 710 laser scanning confocal microscope (Carl Zeiss, Göttingen, Germany) under 400× magnification. Nuclei are stained with Hoechst 33342 (blue) and TUNEL positive staining is red. Negative controls (panels (−).) for TUNEL were performed following the manufacturer’s instructions. Original western blots are presented in File S1.

Caspase-3-like activity was assessed in G1-treated PNT1A, LNCaP, DU145 and PC3 cells as an indicator of apoptosis. All cell lines exhibited increased caspase-3 like activity following GPER activation by G1 (1.42 ± 0.01, 1.46 ± 0.04, 1.14 ± 0.02, and 1.47 ± 0.08 fold-change relative to control in PNT1A, LNCaP, DU145 and PC3 cells, respectively, Figure 8A).

To shed light on the apoptotic pathways being activated by G1, we analysed the expression of several regulators of both the intrinsic and extrinsic pathways of apoptosis [52]. The expression of the mitochondrial pro-apoptotic protein Bax was increased in G1-treated LNCaP cells (1.68 ± 0.19 fold-change relative to control, Figure 8B,E), whereas Bcl-2 (anti-apoptotic) levels remained unchanged (Figure 8B,E). Accordingly, Bax/Bcl-2 ratio was significantly increased in G1-treated LNCaP cells compared with controls (1.7 fold-increase, Figure 8C,E). In contrast, Bax and Blc-2 expression, as well as the Bax/Bcl-2 ratio, remained unchanged in DU145 (not expressing Bax) and PC3 cells following G1 treatment (Figure 8B,C,E). Expression levels of the initiator caspase-9 precursor were increased in LNCaP and DU145 cells upon G1 exposure (1.33 ± 0.12 and 1.48 ± 0.13 fold change to control, Figure 8B). p53 expression was increased following G1 treatment exclusively in LNCaP cells (2.75 ± 0.13 fold-change relative to control, respectively, Figure 8B,E). No changes were observed in DU145 cells, and PC3 cells are known to be p53-null [53].

G1 also increased the expression of the precursor of the initiator caspase of the extrinsic apoptotic pathway, caspase-8, in LNCaP cells (1.88 ± 0.28 fold-change relative to control, Figure 8D,E). Stimulation with the GPER agonist enhanced the expression of Fas receptor in DU145 cells (1.51 ± 0.17 fold-change relative to control) and FasL ligand in LNCaP (1.31 ± 0.02 fold change relative to control) and PC3 cells (1.39 ± 0.12 fold-change relative to control, Figure 8D,E).

TUNEL assay results (Figure 8F,G) showed that DNA fragmentation was significantly increased in G1-treated cells compared with their respective controls (7.7 ± 0.07, 1.60 ± 0.12 and 3.89 ± 0.59 fold-change in LNCaP, DU145 and PC3 cells, respectively, Figure 8F). Consistently, flow cytometry confirmed that the sub-G1 population was increased following G1 treatment by approximately 11%, 9% and 8% in LNCaP, DU145 and PC3 cells, respectively (Figure 8D).

### 3.6. Migration and Invasion of PCa Cells Were Shaped by GPER

**Figure 9:**
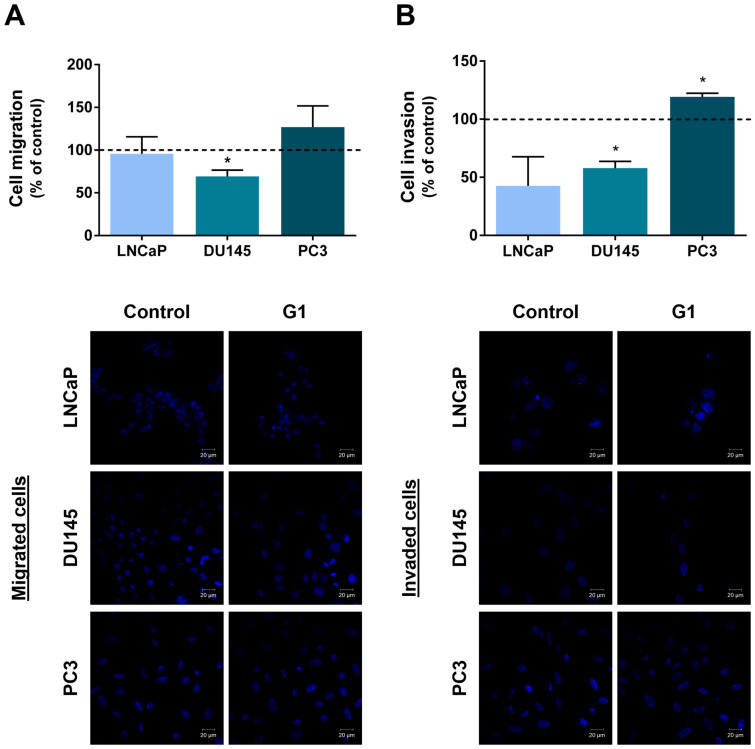
Effect of G1 in LNCaP, DU145 and PC3 human PCa cells migration and invasion. (**A**) Cell migration and (**B**) cell invasion determined by trans-wells assays in uncoated and Matrigel-coated chambers, respectively. The upper chambers contained serum-free medium and cells in the presence or absence of G1 (1 µM). FBS-complete medium in the lower chambers was used as a chemoattractant. Cells were stained, and the number of migrated or invaded cells per 400× magnification field (five fields were assessed for each experimental condition) after 24 h was counted. Results are expressed as % of the control untreated group (0 µM G1, dashed line). Error bars indicate mean ± S.E.M. (n = 3) * *p* < 0.05. Right panels show representative fluorescence microscopy images of migrated and invaded cells in control and G1-treated (1 µM) cells. Images were acquired using a Zeiss LSM 710 laser scanning confocal microscope (Carl Zeiss, Göttingen, Germany) under 400× magnification. Nuclei are stained with Hoechst 33342.

G1 treatment significantly reduced DU145 cell migration and invasion by approximately 30 and 42%, respectively, compared with control cells (Figure 9A,B). In contrast, G1-treated PC3 cells exhibited increased invasion (~19% relative to control), although no statistically significant differences were observed in migration (Figure 9A,B). LNCaP cell migration and invasion were not affected by G1 treatment (Figure 9A,B).

### 3.7. GPER Activation Modulated the Expression of Epithelial and Mesenchymal Markers in PCa Cells

Acquisition of invasive properties is closely associated with EMT, which includes, among other alterations, reduced E-cadherin expression and loss of cytokeratin 8 and 18, whereas N-cadherin and vimentin levels are increased [54,55,56]; thus, we examined the expression of these EMT-related markers.

**Figure 10:**
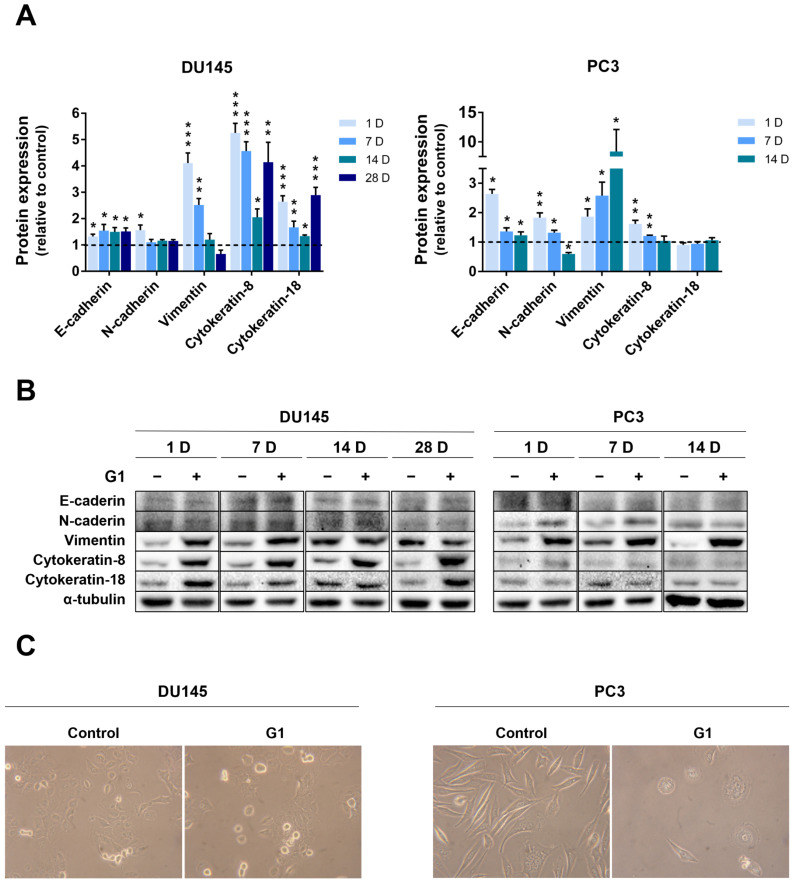
G1 effects in modulating the expression of epithelial and mesenchymal markers in DU145 and PC3 human PCa cells in long-term culture. Cells were treated with 1 µM G1 for 1 to 28 days (1–28 D). (**A**) Protein expression determined by WB analysis after normalisation with α-tubulin. Results are expressed as fold-change relative to the control untreated group (0 µM G1, dashed line). Error bars indicate mean ± S.E.M. (n = 5) * *p* < 0.05 ** *p* < 0.01 *** *p* < 0.001. (**B**) Representative immunoblots. (**C**) Representative images of DU145 and PC3 cells morphology after 28 days stimulation with G1 compared with the control. Images were obtained in optical microscope under 200× magnification. Original western blots are presented in File S1.

CRPC cell lines DU145 and PC3 were exposed long-term to 1 µM G1, and the expression of epithelial and mesenchymal markers was evaluated at 1, 7, 14 and 28 days of treatment. Prolonged G1 exposure increased E-cadherin expression from day 1 onwards in both DU145 and PC3 cells (Figure 10A,B), with a more pronounced effect observed at early time points in PC3 cells (Figure 10B). A marked increase in cytokeratin 8 expression was observed in G1-treated DU145 cells (Figure 10A), whereas a modest elevation was detected in PC3 cells up to 14 days of treatment (Figure 10B).

In PC3 cells, the G1-induced increase in cytokeratin 8 expression was no longer observed after 14 days of exposure (Figure 10B). Cytokeratin 18 also markedly upregulated by G1 stimulation in DU145 cells, whereas no significant differences were found in PC3 cell in any time point analysed (Figure 10A,B). Regarding N-cadherin, although its expression was initially increased in response to G1, no sustained alterations were observed in DU145 cells from day 7 until the end of the treatment period (Figure 10A). Similarly, in PC3 cells, N-cadherin expression was increased after 24 h of treatment and remained higher than in the control cells at day 7; however, after 14 days, its expression was reduced compared with control levels (Figure 10B). Vimentin expression was also increased in G1-treated DU145 cells compared with controls at days 1 and 7. After 14 days, no significant differences were detected, and at 28 days, vimentin expression tended to decrease following treatment (Figure 10A). In contrast, in PC3 cells, vimentin expression markedly increased over time in response to G1 treatment (Figure 10B). Data for PC3 cells at 28 days were not included because insufficient protein was obtained for analysis due to the substantial reduction in cell number in the treated group. Additionally, long-term G1 stimulation induced noticeable morphological changes, as shown in Figure 10C.

### 3.8. GPER Modulated the Metabolic Profile of PCa Cells

**Figure 11:**
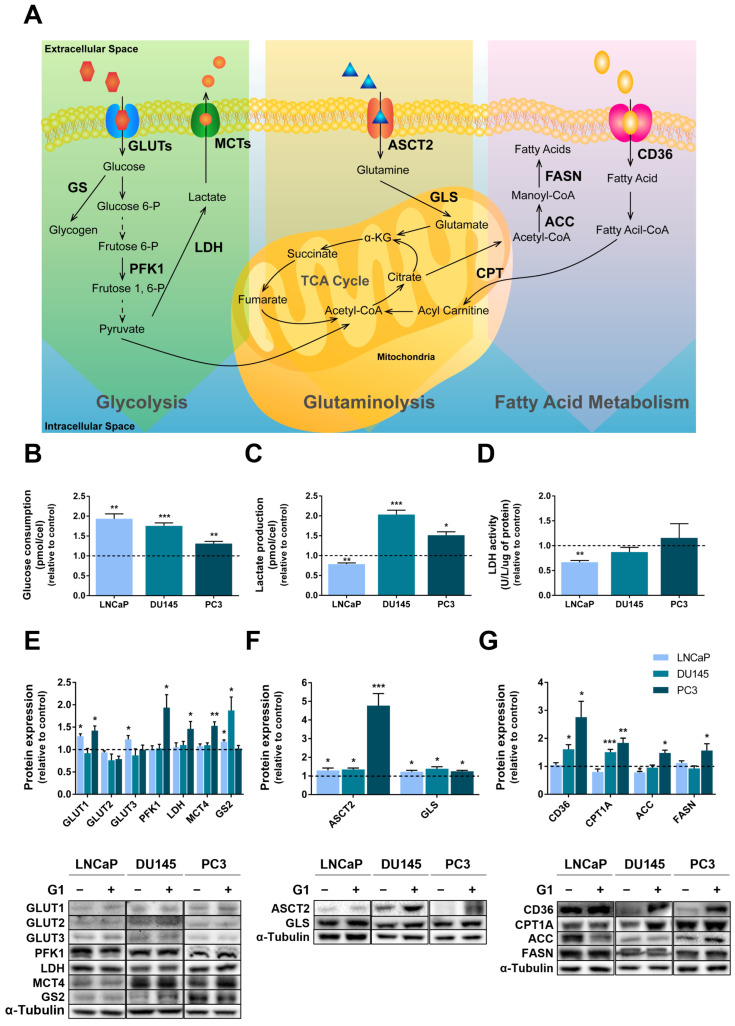
G1 actions in modulating the metabolic profile of LNCaP, DU145 and PC3 human PCa cells. Cells were treated with 1 µM G1 for 24 h. (**A**) Schematic representation of glycolysis, glutaminolysis and fatty acid metabolism. Glucose enters the cell through the activity of GLUTs, being metabolised through glycolytic enzymes, namely PFK1. Pyruvate, the end product of glycolysis, can be driven to mitochondria or converted into lactate by LDH. MCT4 at the cell membrane exports lactate to the extracellular space. Alternatively, glucose can be converted into glycogen through GS. Glutamine is incorporated into the cell by the membrane transporter ASCT2 and is converted to glutamate by the mitochondrial GLS. CD36 transporter is responsible for the uptake of fatty acids that can be incorporated into mitochondria by CPT1A, undergoing β-oxidation. ACC and FASN are the target players in fatty acid de novo synthesis from citrate. (**B**) Glucose consumption and (**C**) lactate production determined by spectrophotometric measurement of glucose and lactate content in the extracellular medium. (**D**) LDH activity determined spectrophotometrically. (**E**–**G**) Protein expression of target players in (**E**) glycolytic metabolism (**F**) glutamine metabolism, and (**G**) lipid handling determined by WB analysis after normalisation with α-tubulin. Results are expressed as fold-change relative to the control untreated group (0 µM G1, dashed line). Error bars indicate mean ± S.E.M. (n = 5) * *p* < 0.05 ** *p* < 0.01 *** *p* < 0.001. Representative immunoblots are shown as bottom panels. Original western blots are presented in File S1.

Considering the importance of glucose and metabolic reprogramming in shaping cancer cell fate [57] and the reported actions of GPER as a metabolic regulator [35], we decided to investigate the influence of GPER in PCa cell metabolism. The effects of G1 treatment on glucose, amino acids and lipid metabolism were evaluated (Figure 11A). G1 stimulation significantly increased glucose consumption in PCa cells (1.93 ± 0.12, 1.76 ± 0.07 and 1.31 ± 0.06 fold-change relative to control in LNCaP, DU145 and PC3 cells, respectively, Figure 11B). The increase in glucose consumption was accompanied by elevated lactate production in G1-treated CRPC cells (2.04 ± 0.11 and 1.51 ± 0.09 fold-change relative to control, respectively, for DU145 and PC3 cells), whereas a reduction was observed in LNCaP cells (0.79 ± 0.03 fold-change relative to control, Figure 11C). LDH, the enzyme responsible for the conversion of pyruvate, derived from glycolysis, into lactate [58], facilitates lactate production, which is subsequently exported to the extracellular space by the MCT4 isoform of MCTs family [58,59] (Figure 11A). G1-treated LNCaP cells displayed reduced LDH activity compared with controls (0.60 ± 0.03 fold-change), whereas no significant differences were observed in the CRPC cell lines (Figure 11D). At protein level, G1 treatment increased LDH and MCT4 expression in PC3 cells (1.46 ± 0.16 and 1.53 ± 0.09 fold-change to control, respectively) but had no effect on their expression in LNCaP and DU145 cells (Figure 11E).

Enhanced glucose consumption in LNCaP and PC3 cells was underpinned by the altered expression of GLUTs (Figure 11E). GPER activation increased GLUT1, and GLUT3 expression was increased with in LNCaP cells (1.30 ± 0.05 and 1.23 ± 0.08 fold-change relative to control), whereas PC3 cells displayed enhanced protein levels of GLUT1 (1.42 ± 0.10 fold-change relative to control, Figure 11E).

PFK1 catalyses the phosphorylation of fructose-6-phosphate (F-6-P) into fructose 1,6-biphosphate (F-1,6-P), which is a limiting step of glycolysis [60] and regulates the glycolytic flux (Figure 11A). G1 treatment significantly increased PFK1 expression in PC3 cells (1.94 ± 0.29 fold-change to control), whereas no significant differences were observed in the other cell lines (Figure 11E).

If glucose is not channelled into glycolysis, it may be stored as glycogen via glycogen synthase (GS, Figure 11A) [61]. Both LNCaP and DU145 G1-treated cells displayed increased expression of GS isoform 2 (GS2) (1.18 ± 0.04 and 1.87 ± 0.30 fold-change relative to control, respectively, Figure 11E).

Regarding amino acid metabolism, we evaluated glutamine handling (Figure 11A). Glutamine uptake is mediated by the glutamine transporter ASCT2 and subsequently converted into glutamate by GLS [62] (Figure 11A). G1 treatment increased ASCT2 and GLS expression in all cell lines compared with their respective controls (LNCaP: 1.39 ± 0.12 and 1.23 ± 0.07 fold-change, respectively; DU145: 1.36 ± 0.07 and 1.39 ± 0.10 fold-change, respectively; PC3: 4.77 ± 0.65 and 1.25 ± 0.05 fold-change, respectively, Figure 11F).

GPER activation also modulated the expression of several targets involved in lipid metabolism, including, CD36, CPT1A, ACC and FASN (Figure 11A). G1 treatment increased CD36 expression in the CRPC cell lines DU145 and PC3 (approximately 2- and 3-fold change relative to control, respectively) (Figure 11G). Similarly, CPT1A, which is required for FA transport into mitochondria for β-oxidation [63] (Figure 11A), was upregulated by G1 treatment in these cell lines (1.51 ± 0.10 and 1.84 ± 0.17 fold-change relative to control in DU145 and PC3 cells, respectively, Figure 11G). ACC and FASN, key lipogenic enzymes involved in de novo lipid synthesis [64,65,66] (Figure 11A), were also increased in PC3 cells following G1 treatment (1.48 ± 0.10 and 1.57 ± 0.24 fold-change relative to control; Figure 11G). In contrast, in LNCaP cells, only ACC expression was altered upon G1 treatment, showing an approximate 20% reduction compared with control (Figure 11G).

### 3.9. Oxidative Stress Was a Target of GPER in PCa Cells

**Figure 12:**
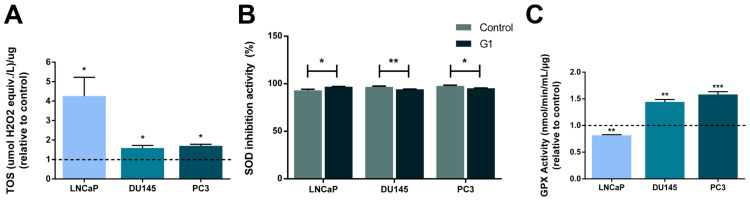
Effect of G1 in modulating the oxidant and antioxidant status of LNCaP, DU145 and PC3 human PCa cells. Cells were treated with 1 µM G1 for 24 h. (**A**) Total oxidant status, (**B**) SOD activity determined spectrophotometrically, and (**C**) GPX activity determined spectrophotometrically. Results are expressed as fold-change relative to the control untreated group (0 µM G1, dashed line). Error bars indicate mean ± S.E.M. (n = 5) * *p* < 0.05 ** *p* < 0.01 *** *p* < 0.001.

Chronic oxidative stress with elevated levels of reactive oxygen species (ROS) is linked to apoptotic and metabolic alterations [67,68] and has been implicated in the onset of PCa [69,70], prompting us to investigate this biological process upon GPER activation. The effects of G1 on the oxidative status of PCa cells were evaluated. G1 stimulation increased TOS in all PCa cells (4.27 ± 0.96, 1.59 ± 0.13 and 1.70 ± 0.08 fold-change to control in LNCaP, DU145 and PC3 cells, respectively; Figure 12A). SOD inhibition activity was slightly increased in LNCaP following G1 treatment (3.8%; Figure 12B), whereas a slight reduction was observed in DU145 and PC3 cells (2.7% and 2.5%, respectively; Figure 12B). Furthermore, G1 stimulation reduced GPx activity in LNCaP cells (0.82 ± 0.01 fold-change relative to control; Figure 12C), whereas it was increased in DU145 and PC3 cells (1.44 ± 0.05 and 1.58 ± 0.05 fold-change relative to control; Figure 12C).

## 4. Discussion

This study demonstrates that GPER modulates multiple hallmarks of PCa, including cell proliferation, apoptosis, migration, invasion, EMT, metabolic reprograming, and oxidative stress. It was also shown that GPER immunoreactivity was higher in human PCa than in BPH, with histological analysis confirming GPER expression in epithelial and stromal cells, both in BPH and PIN, which follows previous studies [26,31,32,33,71] and supports the existence of oestrogen signalling mediated by GPER in the prostate stromal and epithelial compartments.

As a novelty, this study first reports an inverse correlation between GPER expression and PSA levels in PCa specimens, suggesting that higher GPER levels may be associated with less aggressive PCa stages and that GPER may exert anti-proliferative effects that indirectly limit tumour burden and PSA production. However, mechanistic studies are required to confirm this association. Moreover, this inverse correlation raises curiosity about the potential role of GPER as a predictive biomarker of biochemical recurrence after radical prostatectomy, together with PSA.

Consistent with immunoreactivity results, we found that all cell lines express GPER, with higher levels detected in androgen-sensitive LNCaP cells than in non-neoplastic PNT1A or CRPC cells. The reduced levels of GPER in CRPC cell lines follow the findings reported in human PCa cases, indicating a correlation between decreased GPER expression and disease aggressiveness and progression from moderately to poorly differentiated PCa [33]. The limited number of TMA samples across different disease stages and the absence of information regarding the CRPC versus androgen-responsive stage of disease do not allow the confirmation of the stage-dependent changes in GPER expression. Despite this limitation, the increased GPER expression in early lesions and LNCaP cells comparatively to PNT1A may suggest a protective or compensatory mechanism that becomes lost during tumour progression.

Our experimental design, and treatment with DHT, the biologically active androgen in the prostate produced locally from testosterone by 5α-reductase [72], underscores the differential expression observed between androgen-sensitive and CRPC cell lines. DHT downregulated GPER protein expression in PNT1A (24 h) and LNCaP (48 h) cells but not in AR-null DU145 and PC3 cells. Lam et al. [28] also observed that DHT treatment (0.1 and 1 nM for 4 days) reduced the GPR30 mRNA levels in LNCaP cells, with the effect being abolished in the presence of the androgen receptor antagonist bicalutamide, or by siRNA silencing of the receptor. Overall, these data support GPER as a DHT-regulated target.

Although GPER is classically described as a cell membrane receptor, it has also been detected in the endoplasmic reticulum, Golgi apparatus, and nucleus [33,73,74,75,76,77,78,79,80]. However, no reports exist quantifying the subcellular distribution of GPER in PCa cells. Herein, we demonstrated the GPER localisation at the cell membrane and endoplasmic reticulum, with residual nuclear staining, and endoplasmic reticulum localisation markedly higher than that at the cell membrane, as reported in MDA-MB231 and HEC50 cells [81]. Moreover, the selective GPER agonist increased the receptor localisation at the cell membrane in the non-neoplastic PNT1A and neoplastic DU145 cells, without affecting nuclear staining. This represents the first evidence that G1 modulates GPER membrane location, which may have functional relevance given that membrane-associated GPER mediates G protein activation, cAMP production, and intracellular calcium mobilisation [13,82,83,84]. G1 also reduced GPER association with the endoplasmic reticulum in PNT1A cells, whereas it was increased in PC3, suggesting that G1 modulates GPER subcellular localisation involving cell type- and context-dependent mechanisms and may play a role in endoplasmic reticulum GPER localisation, linked to receptor synthesis and cellular trafficking (endoplasmic reticulum—Golgi before its translocation to the cell membrane (93, 94); constitutive retrograde transport from the plasma membrane to the endosomal compartment (104–106) or to active intracellular calcium signalling [84,85]).

Considering the effects of G1/GPER in regulating prostate cell behaviour, low G1 concentrations increased viability in non-neoplastic PNT1A cells, whereas the highest G1 concentration reduced viability, consistent with the biphasic effects reported for E_2_ and other estrogenic compounds in prostate models [86,87]. In contrast, GPER activation markedly reduced viability in CRPC cells, which were more sensitive to G1 than LNCaP cells. LNCaP, DU145 and PC3 cells also displayed reduced proliferation index in response to G1, corroborating previous findings that G1 inhibits PCa cell growth [26]. These results support GPER as a potential therapeutic target in PCa, in line with a panoply of studies describing its anti-proliferative effects in other cancer types [16,19,21,88,89,90,91].

Despite the effect of G1 in diminishing the number of Ki-67 positive cells, the expression of this proliferation marker seemed to be higher in the nuclei of treated cells. Cell cycle analysis clarified this finding, demonstrating that G1 induced G2/M arrest in PCa cells, which follows a previous report on LNCaP and PC3 cells [26]. As Ki-67 expression varies across the cell cycle and is elevated during G2 and mitosis [92,93], this likely explains the apparent increase in nuclear staining.

G1 effects in suppressing the viability and proliferation of CRPC cells were underpinned by the altered expression of key cell cycle and cell survival regulators. G1 upregulated SCF in PC3 cells, whereas full-length c-Kit expression was reduced in LNCaP and DU145 cells, concomitant with decreased proliferation. Although G1 also decreased the proliferation of PC3 cells, no changes were found in the expression of the full-length c-Kit.

Regarding the oncogene c-Myc, our study showed that p-cMyc levels were markedly increased in all neoplastic cell lines following G1 treatment, in line with reduced proliferation. Repression of p21 expression is one of the mechanisms by which c-Myc promotes cell proliferation and cell cycle progression [94,95,96]. However, no changes in p21 expression in PCa cells treated with the GPER agonist were detected, suggesting the involvement of another molecular target downstream p-cMyc.

GPER-mediated regulation of proliferation and cancer growth have been described in various cell types, involving several molecular targets and signalling pathways, namely phosphatidylinositol 3-kinase (PI3K), serine/threonine protein kinase AKT and mitogen-activated protein kinase (MAPK)/extracellular signal-regulated kinase (ERK)1/2 [14,19,89,97,98,99,100,101]. Herein, despite the reduced proliferative ability of PCa cells treated with G1, no changes were observed in the expression of AKT and MAPK. The increased expression of p-AKT in the androgen-sensitive LNCaP cells would suggest the activation of this pathway and enhanced survival. However, no stimulatory effect by G1 was observed in LNCaP cell growth. There is no definitive explanation for this fact at the moment, which is likely related to the observed downregulated expression of p-MAPK. Similar findings were obtained with the selective oestrogen receptor modulator raloxifene. Modulation of GPER activity by raloxifene increased p-AKT expression and reduced p-ERK, suppressing LNCaP cells’ viability [102].

G1 induced apoptosis in all the PCa cells, as indicated by increased caspase-3-like activity, a key endpoint of intrinsic and extrinsic apoptotic signalling pathways [103]. These findings were supported by the increase in DNA fragmentation in G1-treated LNCaP, DU145 and PC3 cells.

G1 enhanced the expression of proapoptotic Bax protein and caspase-9 in LNCaP and DU145 cells (only caspase-9), supporting activation of the intrinsic apoptosis pathway. In LNCaP cells, apoptosis was further reinforced by an increased Bax/Bcl-2 protein ratio, recognised as a powerful marker of cell apoptosis linked to the activation of the intrinsic pathway [104,105]. The Bax/Bcl-2 ratio could not be determined in DU145 cells, as these cells do not express the Bax protein, which was also reported by others [106,107,108]. In G1-treated LNCaP cells, the enhanced apoptotic rate is also supported by the increased expression of the tumour suppressor p53, which is widely known to control the intrinsic pathway [109]. p53 acts as a transcription factor controlling the expression of several apoptotic regulators, namely inducing Bax and repressing Bcl-2 transcription, thereby establishing an increased proapoptotic Bax/Bcl-2 protein ratio [110]. In addition, p53 promotes the transcription of Bcl-2 antagonists [111], and the cytoplasmic p53 binds to proapoptotic Bcl-2-family proteins, antagonising Bcl-2 function or leading to permeabilization of mitochondria and the release of cytochrome C [111]. Altogether, results suggest that G1 induces apoptosis of LNCaP cells, likely by activating the intrinsic pathway. Indeed, GPER’s pro-apoptotic actions have been widely reported in distinct types of cancer, including prostate, breast, ovarian and Leydig cell tumours [21,22,26,91,112], and are mainly related to the mitochondrial pathway and altered expression or activity of Bcl-2 protein family members [103]. However, no changes were detected in Bax and Bcl-2 expression in PC3 cells, which can likely be explained by the lack of p53 expression [53]. The same may happen in DU145 cells that also have a mutated non-functional p53 [113]. Although no alterations were observed in caspase-8, a trigger of the extrinsic pathway of apoptosis, increased expression of FasL, was detected in PC3 cells, suggesting that this would be involved in GPER-induced apoptosis. It is also predictable that this pathway is activated in LNCaP and DU145 cells, in line with the increased expression of caspase-8, FasL, and Fas receptor, respectively.

The pro-apoptotic effects of oestrogens have been frequently associated with the activation of the extrinsic pathway of apoptosis [114,115,116], and other studies have also reported enhanced apoptosis of androgen-sensitive LNCaP and CRPC PC3 cells upon GPER activation [26]. However, our results indicate that the GPER agonist G1 can activate apoptotic signalling by both the intrinsic and extrinsic pathways.

Beyond its effects on proliferation and apoptosis, GPER activation also influenced other cancer hallmarks, namely migration and invasion.

G1 treatment decreased the migration and invasion of DU145 cells, consistent with previous findings linking GPER activation to impaired cytoskeletal organisation and motility [27]. However, G1 had no effect on LNCaP cells and increased invasion in PC3 cells. There is no definitive explanation for this fact at this stage. However, it may be attributed to the distinct molecular backgrounds of these models, as PC3 cells represent an AR-negative, PTEN-deficient and highly aggressive CRPC phenotype [117], which may drive the distinct observed responses.

In addition to the morphological changes, long-term G1 stimulation induced marked alterations in the expression of epithelial and mesenchymal markers. In DU145 cells, increased expression of the cell–cell adhesion protein E-cadherin over N-cadherin, as well as the epithelial cellular intermediate filament proteins, cytokeratin 8 and 18, throughout the entire experimental time frame suggests that sustained GPER activation may partially reinforce the epithelial phenotype and counteract EMT. Evidence regarding GPER involvement in EMT in PCa is scarce. However, similar EMT-suppressive effects of GPER have been reported in breast and pancreatic cancer models [118,119]. Nevertheless, the early concomitant increase in both E- and N-cadherin (day 1), together with fluctuations in the expression of cytokeratin 8 and 18, may also reflect transient epithelial plasticity during dynamic phenotypic adaptation. A study on PC3 cells also showed that GPER activation reduced vimentin and increased E-cadherin expression [119]. Herein, our data indicated that long-term G1 exposure (14 days) increased E-cadherin and reduced N-cadherin levels in PC3 cells, yet vimentin expression remained markedly elevated. The different patterns in the invasiveness capacity of DU145 and PC3 cells in response to G1 may be underpinned by the observed differences in the expression of EMT markers.

Metabolic reprogramming is a recognised hallmark of cancer [57,120], and emerging evidence indicates that GPER is a metabolic regulator, particularly in glucose homeostasis [35]. GPER knockout mice exhibit hyperglycaemia and glucose intolerance [121], highlighting GPER’s relevant actions in glycolytic metabolism. Accordingly, our results showed that G1 treatment altered the metabolic profile of androgen-sensitive LNCaP cells and CRPC cells, increasing glucose consumption, as evidenced by the increased expression of GLUT1 (LNCaP and PC3) and GLUT3 (LNCaP), the GLUT isoforms mostly associated with cancer [122]. Despite the increased glucose consumption, GLUT1 and GLUT3 expression remained unchanged in G1-treated DU145 cells, indicating that another GLUT isoform may be involved. A likely candidate is GLUT12, which has been detected in PCa cases and is suggested as one of the main glucose suppliers for glycolytic metabolism [123,124,125]. These novel findings linking GPER with the regulation of glycolytic metabolism in PCa cells align with recent reports linking GPER to GLUTs regulation in other cell types [126,127,128].

After entering the cells, glucose follows the glycolytic pathway, being converted into pyruvate, the end product of glycolysis [60]. PFK1 catalyses a limiting step of glycolysis, the conversion of fructose 6-phosphate into fructose 1,6-bisphosphate, and has been considered to indicate the rate of glycolytic flux [60]. GPER activation increased PFK1 expression in PC3 cells, which were already described as being more glycolytic than in LNCaP cells [129]. Indeed, in G1-treated PC3 cells, the increased glucose consumption was accompanied by increased lactate production, supported by the increased expression levels of LDH, the enzyme responsible for the conversion of pyruvate into lactate [58], and MCT4, the transporter responsible for lactate export to the extracellular space [58,59]. In DU145, the increased lactate production was not followed by changes in the expression of PFK, LDH or MCT4, suggesting alternative metabolic sources for lactate [130,131]. Moreover, the expression of GS2, the enzyme that converts glucose into glycogen [61], was increased in G1-treated DU145 cells, suggesting that glucose is being routed for storage.

In the less-glycolytic LNCaP cells [129], despite the increased glucose consumption in response to G1 stimulation, lactate production was reduced, in accordance with the diminished LDH activity and the unaltered expression of LDH and MCT4. Similarly to what was found in DU145, G1 also increased the expression of GS2 in LNCaP cells. The implications of putative glucose storage for PCa cell fate have not been investigated, but previous studies reported that glycogen accumulation is associated with androgen-responsiveness [132].

Interestingly, GPER activation could also modulate glutamine metabolism, as G1 increased the expression of ASCT2 and GLS, the membrane glutamine transporter and the enzyme that convert glutamine into glutamate, respectively. Glutaminolysis has been implicated in the onset and progression of PCa [133], and our previous study showed that a restriction on glutamine decreased cell viability and the inhibition of glutamine metabolism by inhibiting GLS activity, which increased apoptosis and reduced PCa cell migration [134]. Further studies are needed to clarify the relevance of G1/GPER modulation of glutaminolysis in PCa proliferative activity.

Lipids are another energy source relevant for sustaining the high proliferative rates of cancer cells [135], and several players involved in lipid metabolism have been shown to be upregulated in PCa cells, namely CD36, FASN and CPT1A [66,136,137,138,139,140]. Although no studies have been directed to investigate GPER as a metabolic regulator in PCa cells, its involvement in lipid handling has been reported in other cell types. For example, GPER activation was able to increase the expression of low-density lipoprotein (LDL) receptor and to induce the uptake of LDL in liver cells (176, 177). Additionally, GPER appears to promote adipogenesis, which led to obesity in female mice (178). Our results show that G1 upregulated the expression of lipogenic enzymes involved in de novo lipid synthesis (ACC and FASN) [64,65,66], fatty acid transporter CD36, and CPT1A, the enzyme required for the transport of fatty acids to the mitochondria for β-oxidation [63]; this indicates that GPER activation in PCa cells seems to drive lipid utilisation by favouring its uptake, synthesis and β-oxidation.

Fatty acid β-oxidation has been reported as an important bioenergetic pathway supporting PCa cell proliferation and tumour growth [139]. However, the acceleration of energy metabolism by GPER, i.e., glucose, glutamine and lipid usage, was not underpinned by increased cell viability and proliferation. On the contrary, G1 suppressed proliferation and promoted apoptosis, suggesting that processes superimpose metabolic plasticity, driving the alterations in PCa cell fate. Nevertheless, this is the first study demonstrating GPER-mediated modulation of PCa cell metabolism, opening new perspectives to explain changes in tumour cell behaviour under oestrogen actions. It will be interesting in the future to investigate the causal relationship between G1 treatment, PCa cell fate and metabolic alterations specifically.

Chronic oxidative stress, resulting from an imbalance between ROS production and the efficiency of antioxidant defences [141], has been implicated in the onset of PCa [69,70]. However, increased oxidative stress above some threshold can trigger programmed cell death [142]. Herein, GPER activation increased TOS in PCa cells, supporting a pro-oxidant shift that may contribute to the pro-apoptotic effects of G1. Other studies also reported the involvement of oestrogens and their receptors in the regulation of oxidative stress [143]. Antioxidant enzymes such as SOD and GPx, which chelate superoxide, preventing and repairing ROS-induced damage, are known targets of oestradiol and G1 [144,145,146], with significantly reduced expression in PCa [147,148,149,150]. SOD catalyses the dismutation of superoxide anion radical into hydrogen peroxide (H_2_O_2_), which is then transformed into water and oxygen by GPx [151,152]. We found a slight increase in SOD activity and a reduction in GPx activity in the androgen-sensitive LNCaP cells, suggesting that increased TOS in LNCaP cells may occur because GPx is not processing the H_2_O_2_ resultant from SOD activity. On the other hand, CRPC cells presented reduced SOD activity and increased activity of GPx, suggesting that the increase in TOS in these cells is motivated by the reduction in SOD activity. Although variations in SOD activity were modest, our findings are supported by the reports in lung cancer cells showing that G1 increased oxidant levels and the activity of antioxidant enzymes SOD and GPx [144].

As mentioned, enhanced oxidative stress can drive PCa cell apoptosis, which we cannot exclude as being related to the enhanced metabolic activity observed. It is well known that the metabolic processes lead to the formation of oxidant species, changing the cell oxidant status [153]. Moreover, it has been shown that high glucose induces oxidative stress and causes activation of several proteins involved in apoptotic cell death, including members of the caspase and Bcl-2 families [154].

## 5. Conclusions

The present results confirm higher GPER expression in PCa cases and its inverse correlation with PSA levels. GPER protein levels were elevated in neoplastic cells, although they were reduced in CRPC cells compared with androgen-responsive cells, with GPER protein levels being modulated by androgens. GPER activation by G1 exerted multiple anti-cancer effects in PCa models, including reduced cell viability and proliferation, G2/M cell cycle arrest, increased apoptosis, and modulation of migration, invasion, and EMT in a cell context-dependent manner. Moreover, GPER activation increased energetic metabolism and oxidative stress in the PCa cells. To the best of our knowledge, this is the first comprehensive report integrating GPER effects across a broad range of cancer hallmarks, including metabolic reprogramming, in PCa. Overall, the obtained findings support an anti-cancer role for GPER and highlight its potential as a therapeutic target. However, the metabolic adaptations induced by GPER activation warrant further investigation, particularly in relation to disease stage and tumour aggressiveness prior to considering GPER as a treatment target in PCa.

## Figures and Tables

**Table 1:** Association of GPER expression with clinical and histopathological data in benign prostate hyperplasia and prostate cancer cases.

Clinical and Histopathological Data	GPER Immunoreactivity		*p* Value ^a^
	Low (Score 1)	High (Score 2)	
Age			
<55	77.78% (7/9)	22.22% (2/9)	0.618
55–65	71.43% (25/35)	28.57% (10/35)	
>65	81.40% (35/43)	18.60% (8/43)	
Diagnosis			
BPH	100.00% (34/34)	0.00% (0/34)	0.000244
PIN	77.78% (7/9)	22.22% (2/9)	
Adenocarcinoma	60.00% (27/45)	40.00% (18/45)	
Total PSA (ng/mL)			
≤4	20.00% (1/5)	80.00% (4/5)	0.047
>4	73.68% (14/19)	26.32% (5/19)	
Free PSA (%)			
≤10	44.44% (4/9)	55.56% (5/9)	0.637
>10	66.67% (6/9)	33.33% (3/9)	
Gleason			
<7	66.67% (8/12)	33.33% (4/12)	0.735
≥7	57.58% (19/33)	42.42% (14/33)	
TNM			
T2	65.00% (13/20)	35.00% (7/20)	1.000
T3	57.14% (4/7)	42.85% (3/7)	
Bone metastasis			
Absent	68.75% (11/16)	31.25% (5/16)	1.000
Present	50.00% (1/2)	50.00% (1/2)	

^a^ Pearson Chi-square correlation; TNM—tumour, node and metastasis (TNM) staging.

## Data Availability

The original contributions presented in this study are included in the article/Supplementary Material. Further inquiries can be directed to the corresponding authors.

## Supplementary Materials

The following supporting information can be downloaded at: https://www.mdpi.com/article/10.3390/cancers18071137/s1, Table S1: Primary antibodies and working dilutions used in the WB analysis; File S1: Original western blots.
