# Supplementary material for: G Protein-Coupled Oestrogen Receptor Actions Targeting the Hallmarks of Cancer in Human Prostate Cells: From Cell Fate to Metabolic Reprogramming
Source: Cancers (Basel). 2026 Apr 1;18(7):1137. doi: 10.3390/cancers18071137 (PMC13072317; doi:10.3390/cancers18071137)
Supplement: Supplementary file 1 [file cancers-18-01137-s001.zip › cancers-4157962-File S1.pdf]

cancers

MDPI

Article

G protein-coupled oestrogen receptor actions targeting the hallmarks of cancer in human prostate cells: from cell fate to metabolic reprogramming

Marília I. Figueira[1,*], Henrique J. Cardoso[1], Lara R.S. Fonseca[2], Tiago M.A. Carvalho[1], Sara Correia[1], Patrícia Arinto[3,4], Rui Henrique[5,6], Adriana O. Santos[1], Cláudio J. Maia[1], Sílvia Socorro[1,*]

[1] RISE-Health, Department of Medical Sciences, Faculty of Health Sciences, University of Beira Interior, Covilhã
[2] RISE-Health, Department of Chemistry, Faculty of Sciences, University of Beira Interior, Covilhã, Portugal
[3] Cancer Genetics Group, IPO Porto Research Center (CI-IPOP)/RISE@CI-IPOP (Health Research Network), Portuguese Oncology Institute of Porto (IPO Porto)/Porto Comprehensive Cancer Center, Porto, Portugal.
[4] Department of Laboratory Genetics, Portuguese Oncology Institute of Porto (IPO Porto)/Porto Comprehensive Cancer Center, Porto, Portugal.
[5] Department of Pathology and Cancer Biology and Epigenetics Group – Research Center, Portuguese Oncology Institute of Porto / Porto Comprehensive Cancer Center Raquel Seruca (Porto.CCC), Porto, Portugal
[6] Department of Pathology and Molecular Immunology, ICBAS-School of Medicine and Biomedical Sciences, University of Porto, Portugal
* Correspondence: SS:ssocorro@fcsaude.ubi.pt and MIF: marilia.figueira@fcsaude.ubi.pt

Original Blots

Below in Figure S1 are provided the original images of the representative blots shown in the manuscript **Figure 2.**

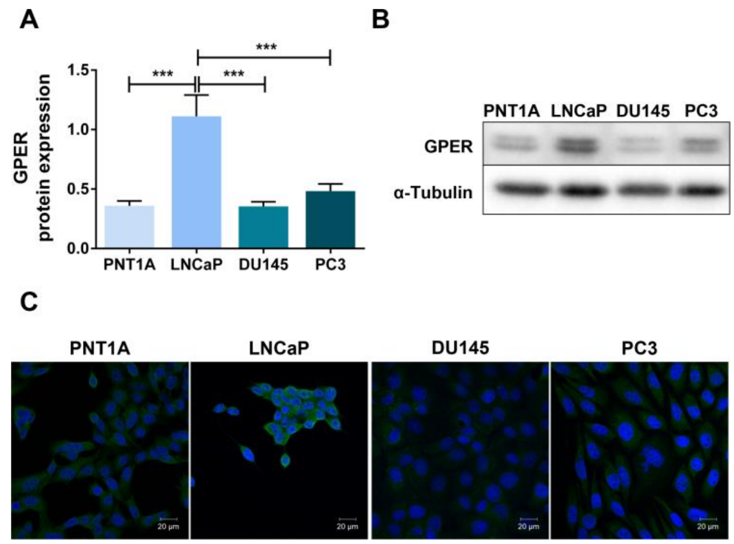

Figure 2. GPER expression in non-neoplastic PNT1A and neoplastic LNCaP, DU145 and PC3 hu-man prostate cells. (A) Protein expression determined by WB analysis after normalisation with α-tubulin. Error bars indicate mean ± S.E.M. (n=3) ***P < 0.001; **(B)** Representative immunoblots; **(C)** Representative images of GPER subcellular localisation obtained with a Zeiss LSM 710 laser scan-ning confocal microscope under 400 X magnification. Nuclei are stained blue and GPER green.

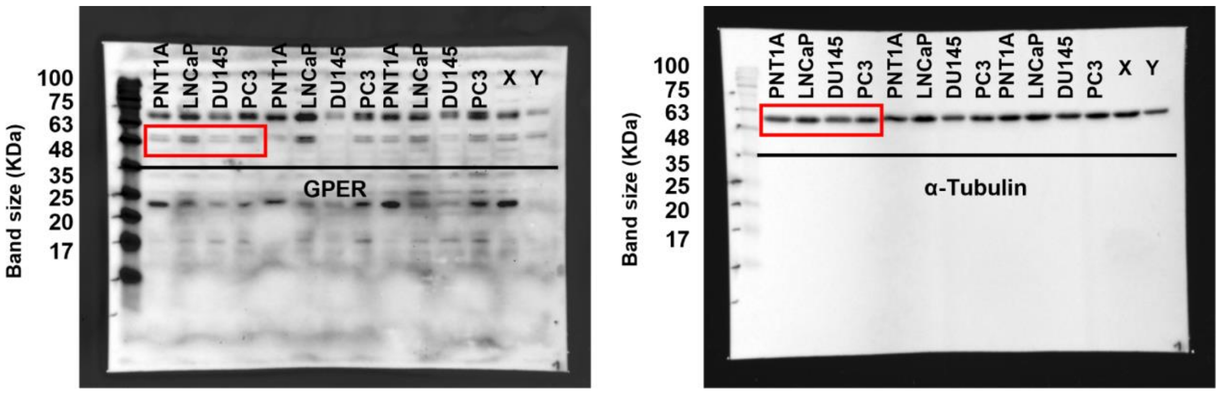

Figure S1. Original Western blots images after GPER and α-tubulin immunodetection in PNT1A, LNCaP, DU145 and PC3 cells protein. Legend: X, Y– other samples analyzed. Red boxes represent the B pairs selected as representative to be shown in Figure 2.

Methodological notes:

To remove the unspecific bands around 56 KDa, a soft stripping protocol was performed after GEPER detection and before incubating the membrane with the anti-α-tubulin antibody.

Below in Figure S2 are provided the original images of the representative blots shown in the manuscript **Figure 4.**

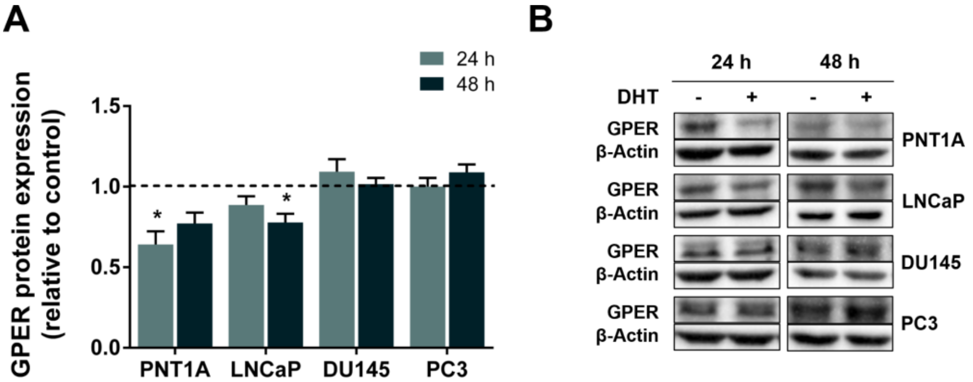

Figure 4. Effect of androgens in regulating GPER expression in LNCaP, DU145 and PC3 human PCa cells. (A) GPER protein expression after treatment with DHT (10 nM) for 24 or 48 h. Protein expression was determined by WB analysis after normalization with β-actin. Results are expressed as fold-change relative to the control untreated group (0 nM DHT, dashed line). Error bars indicate mean ± S.E.M. (n=5) **P* < 0.05. **(B)** Representative immunoblots.

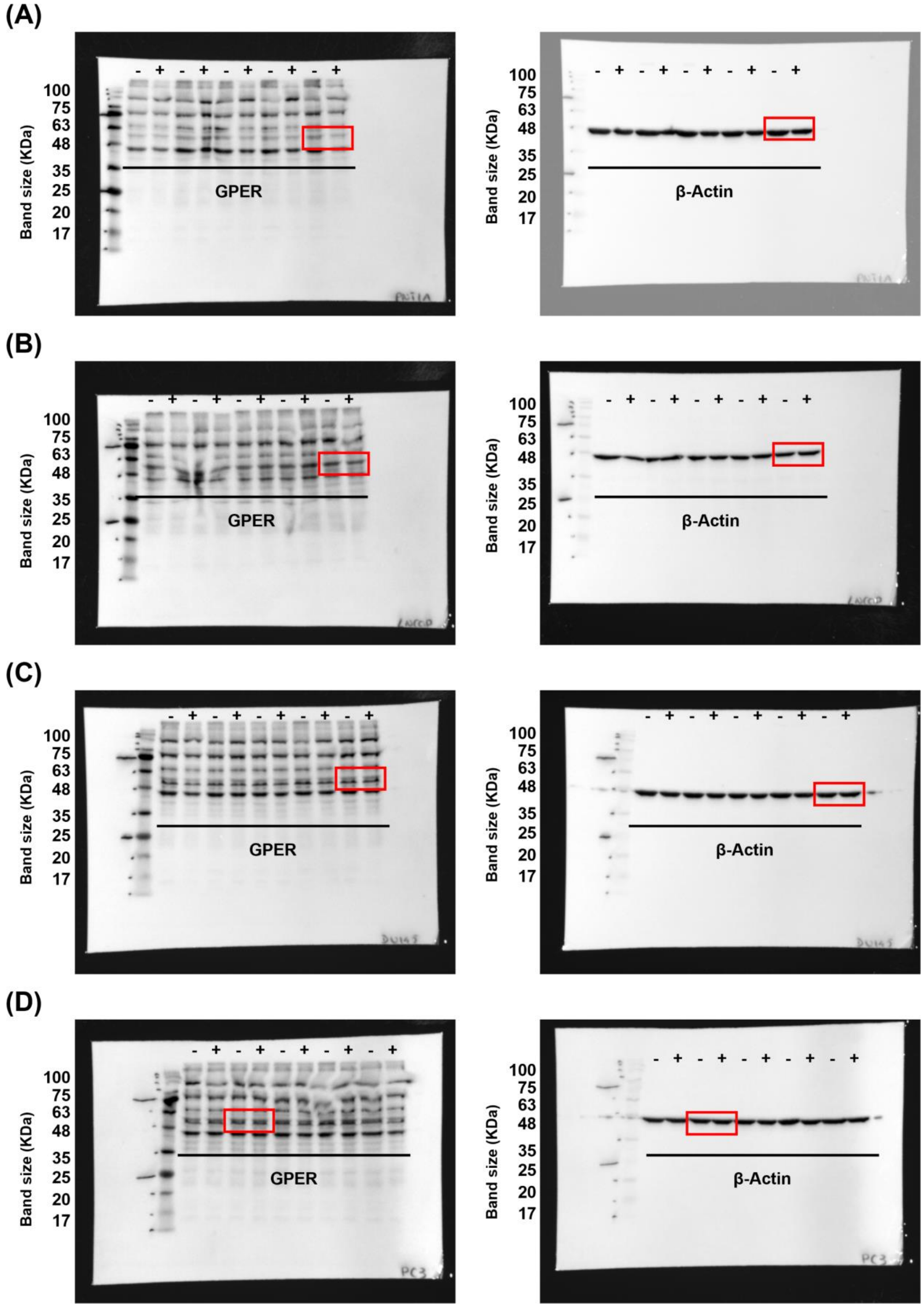
(A)
(B)
(C)
(D)
Band size (KDa)
100
75
63
48
35
25
20
17
- + - + - + - + - +
GPER
β-Actin
PNT1A
LNCaP
DU145
PC3

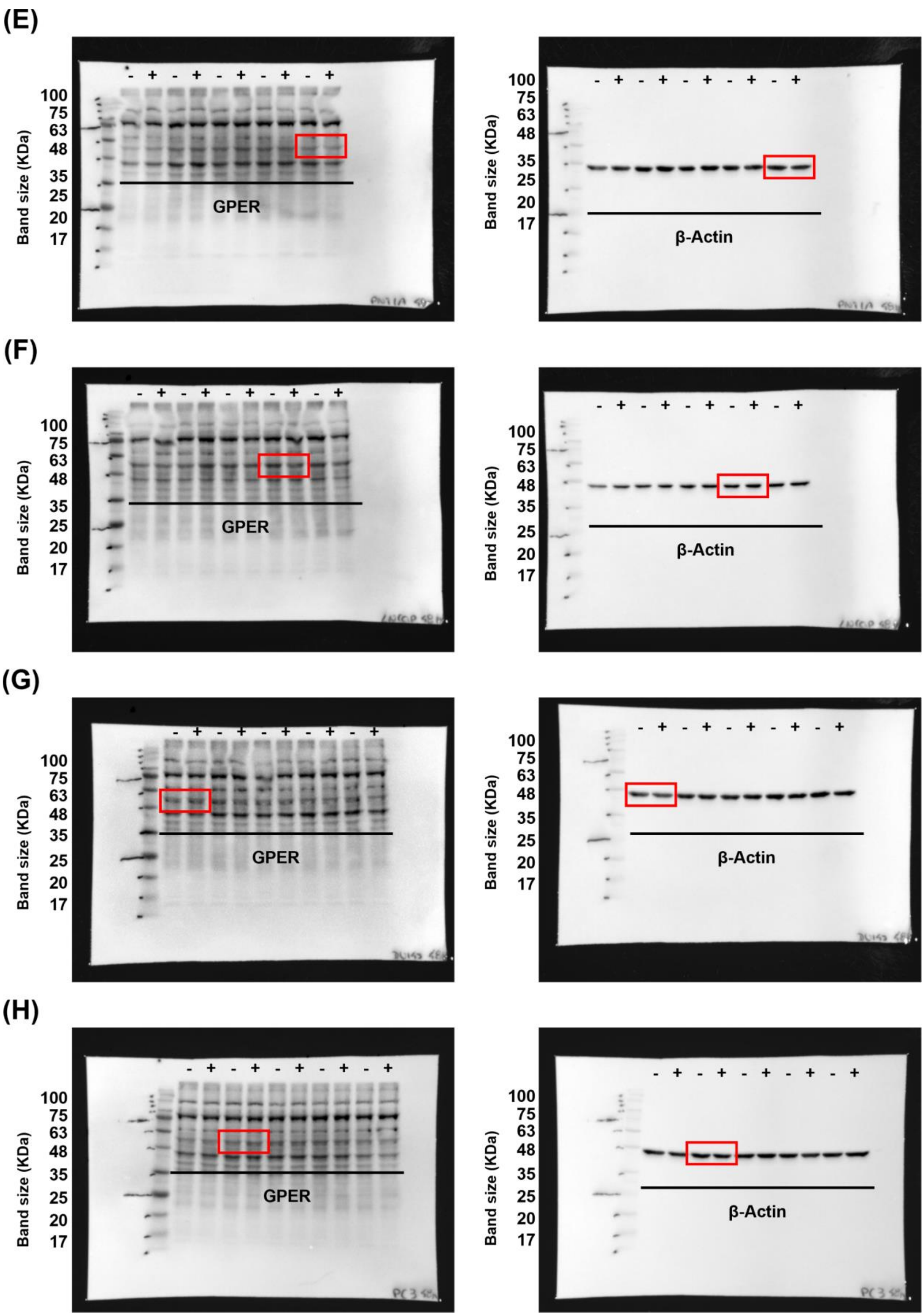

Figure S2. Original Western blots images after GPER and β-actin immunodetection after treatment with DHT for 24 h and 48 h in (A and E) PNT1A, (B and F) LNCaP, (C and G) DU145 and (D and H) PC3 cells protein extracts. Legend: - – control group; + - DHT-treated group. Red boxes represent the B pairs selected as representative to be shown in Figure 4.

Methodological notes:

To remove the unspecific bands around 48 KDa, a soft stripping protocol was performed after GPER detection and before incubating the membrane with the anti-β-actin antibody.

Below in Figure S3 are provided the original images of the representative blots shown in the manuscript **Figure 7.**

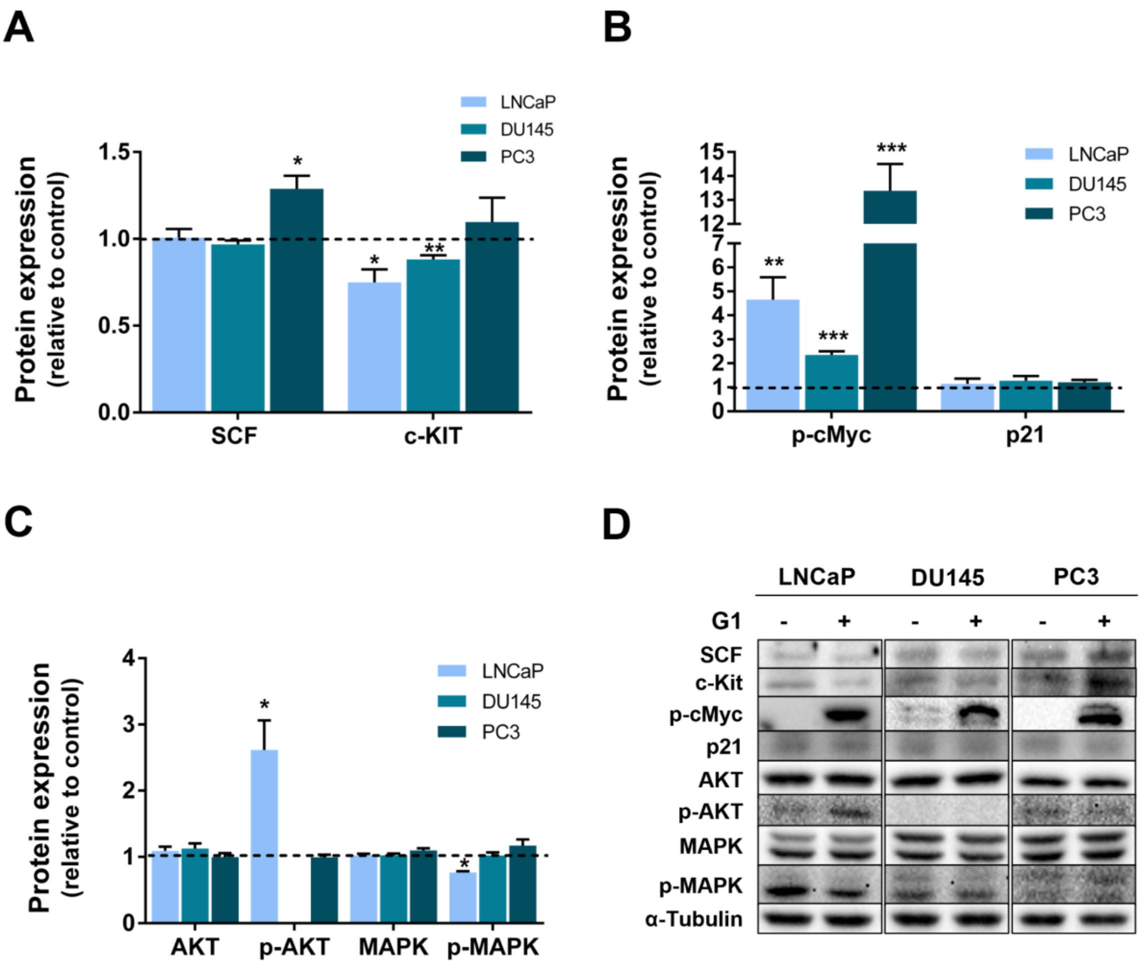

Figure 7. Effect of G1 on the expression of cell cycle regulators and intracellular signalling players in LNCaP, DU145 and PC3 human PCa cells. Cells were treated with 1 μM G1 for 24 hours. Protein expression of **(A)** SCF ligand and receptor tyrosine kinase c-Kit, **(B)** p-cMyc and p21, and **(C)** AKT, p-AKT, MAPK and p-MAPK determined by WB analysis after normalisation with α-tubulin. Results are expressed as fold-change relative to the control untreated group (0 μM G1, dashed line). Error bars indicate mean ± S.E.M. (n=5) *$P < 0.05$; **$P < 0.001$ ***$P < 0.01$. **(D)** Representative immunoblots.

(A)

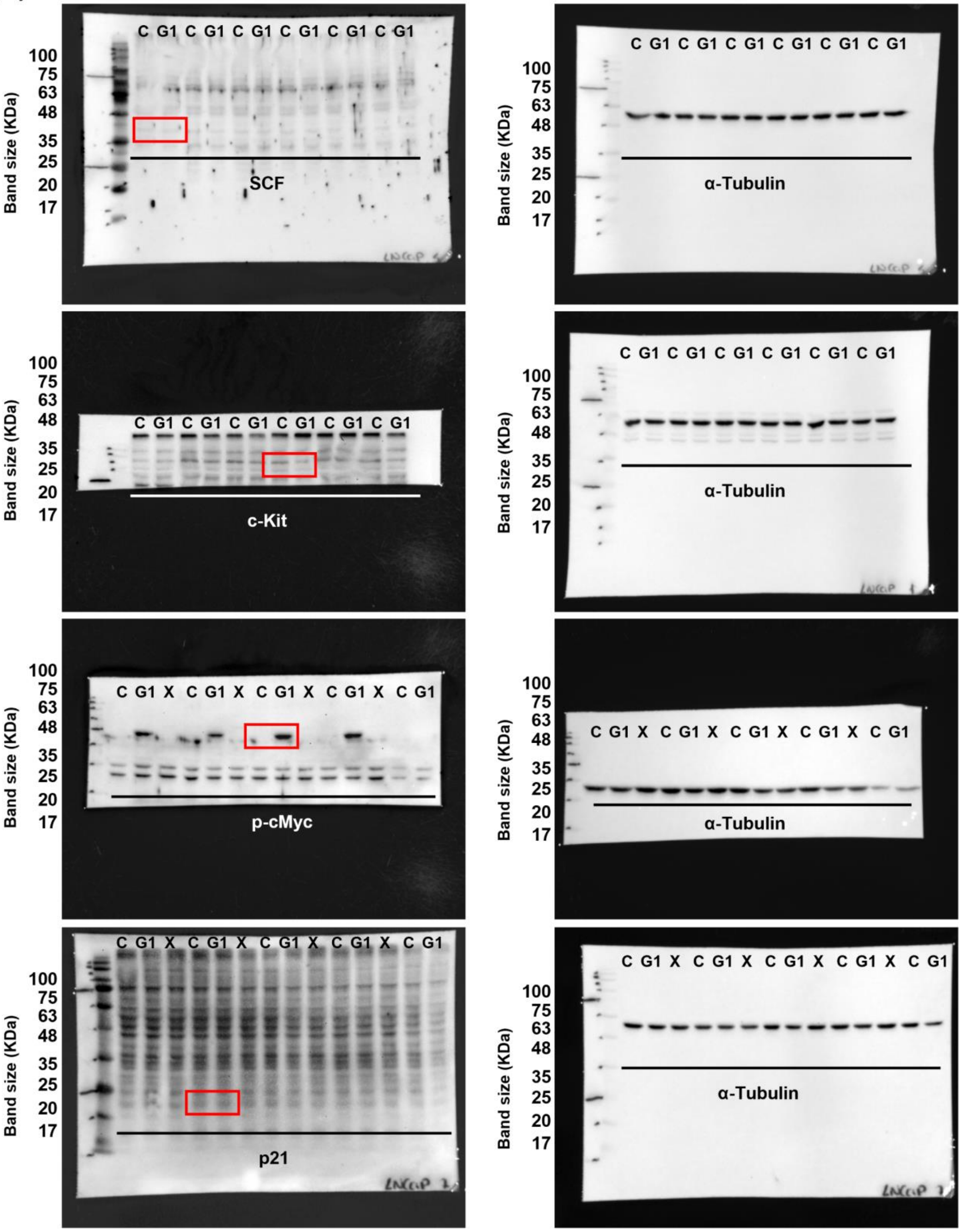

Band size (KDa)
100
75
63
48
35
25
20
17
C G1 C G1 C G1 C G1 C G1 C G1
SCF
α-Tubulin
c-Kit
C G1 X C G1 X C G1 X C G1 X C G1
p-cMyc
p21

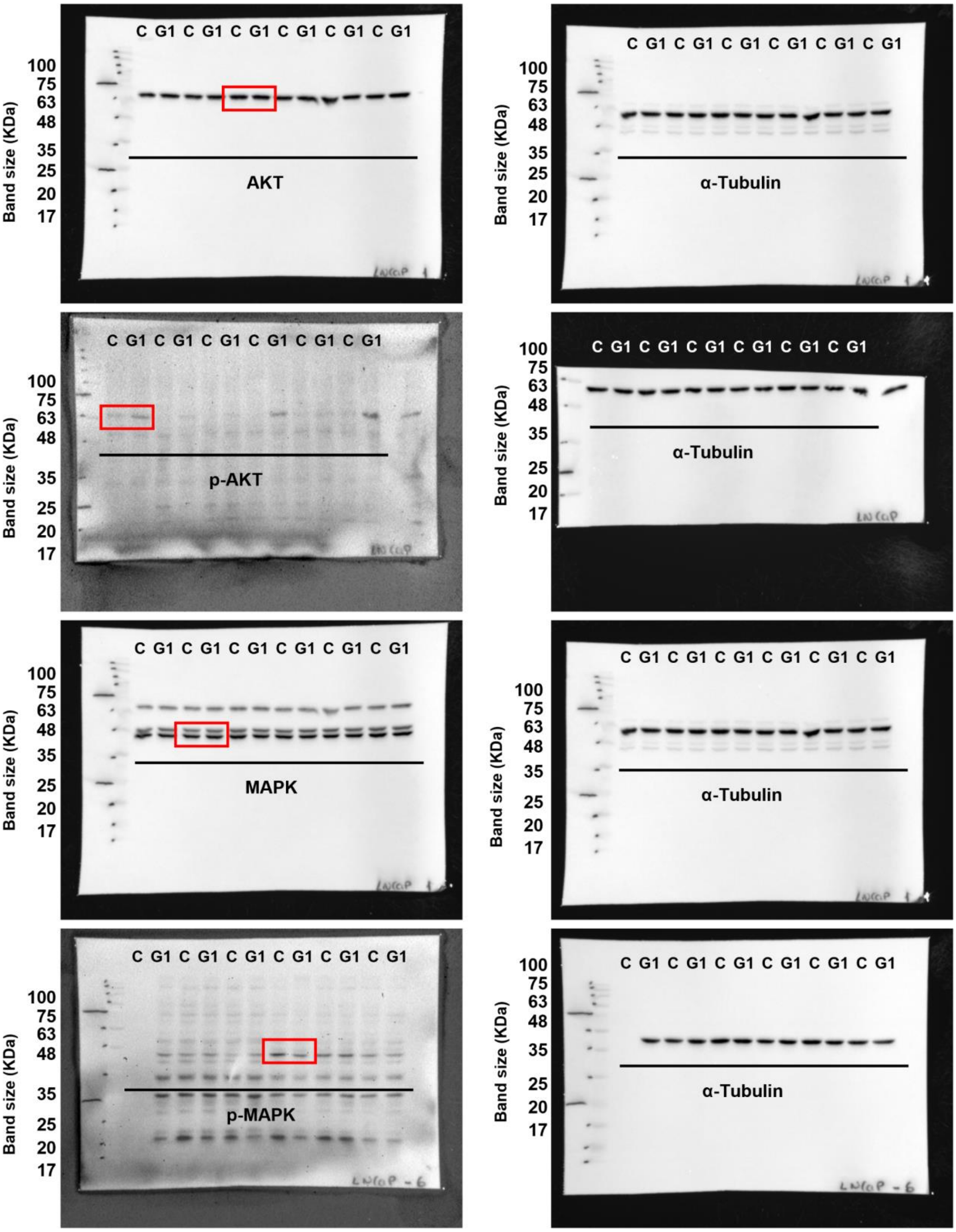
C G1 C G1 C G1 C G1 C G1 C G1
Band size (KDa)
100
75
63
48
35
25
20
17
AKT
α-Tubulin
p-AKT
α-Tubulin
MAPK
α-Tubulin
p-MAPK
α-Tubulin

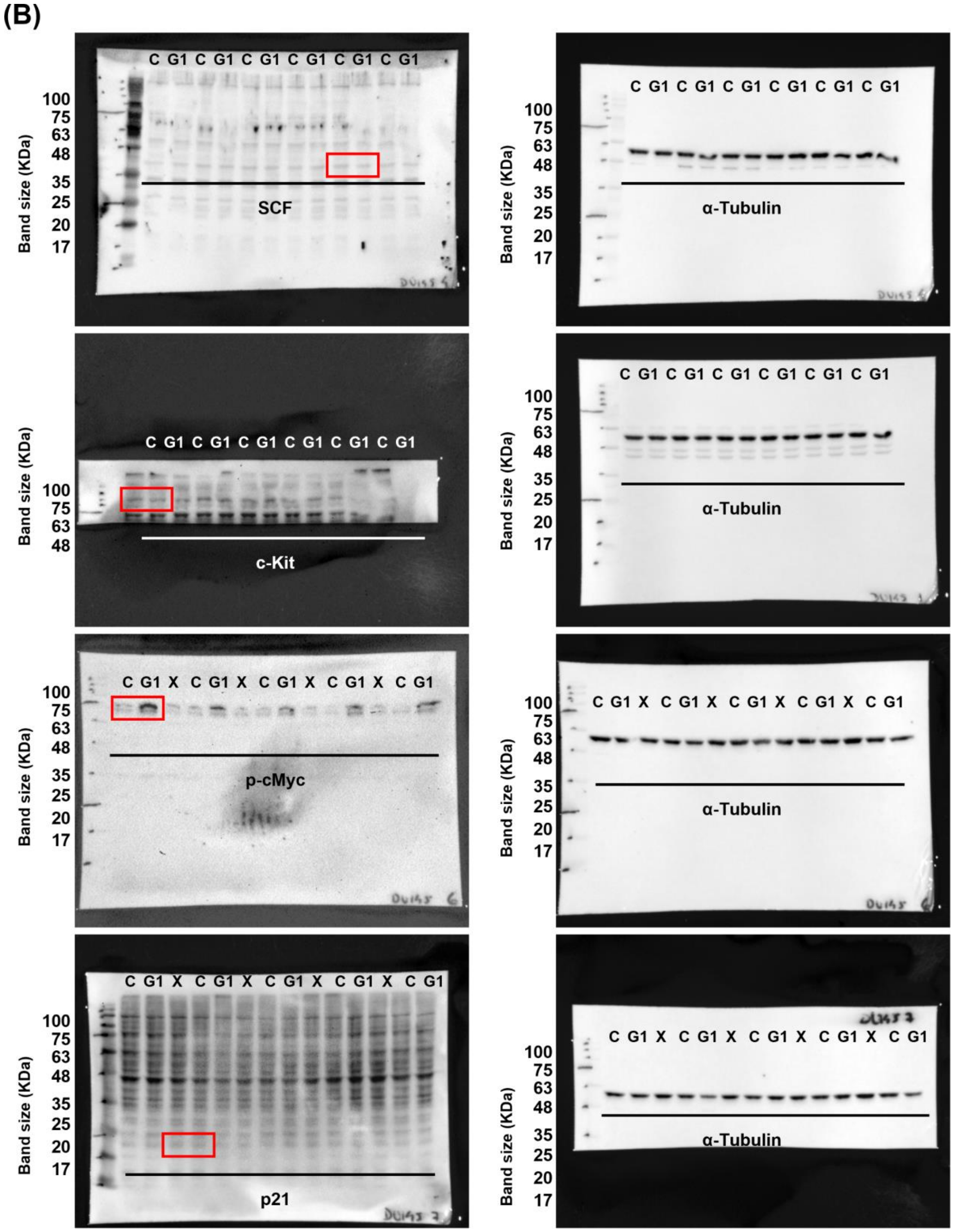
(B)
C G1 C G1 C G1 C G1 C G1 C G1
Band size (KDa)
100
75
63
48
35
25
20
17
SCF
α-Tubulin
c-Kit
C G1 X C G1 X C G1 X C G1 X C G1
p-cMyc
p21

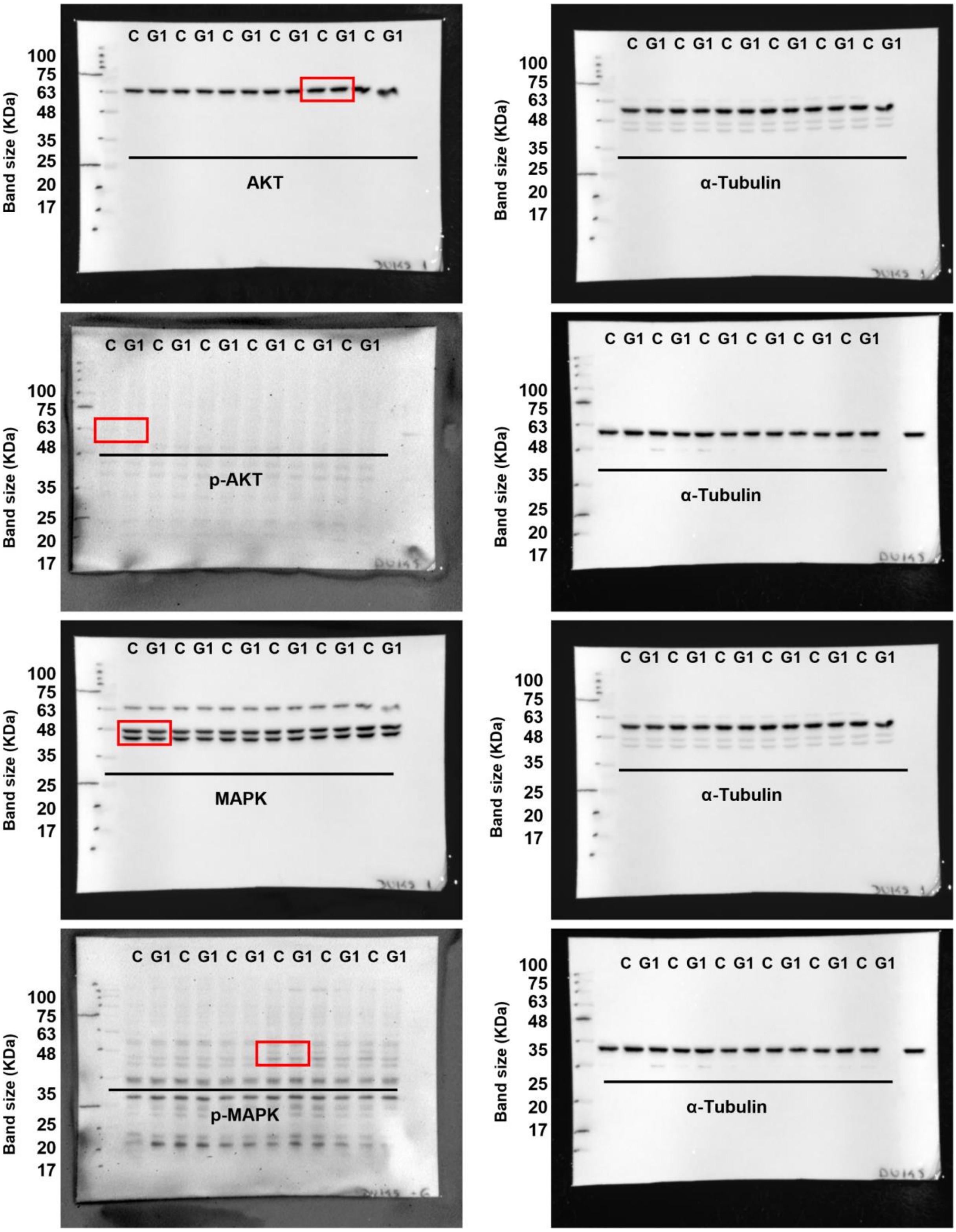
C G1 C G1 C G1 C G1 C G1 C G1
Band size (KDa)
100
75
63
48
35
25
20
17
AKT
α-Tubulin
p-AKT
MAPK
p-MAPK

(C)

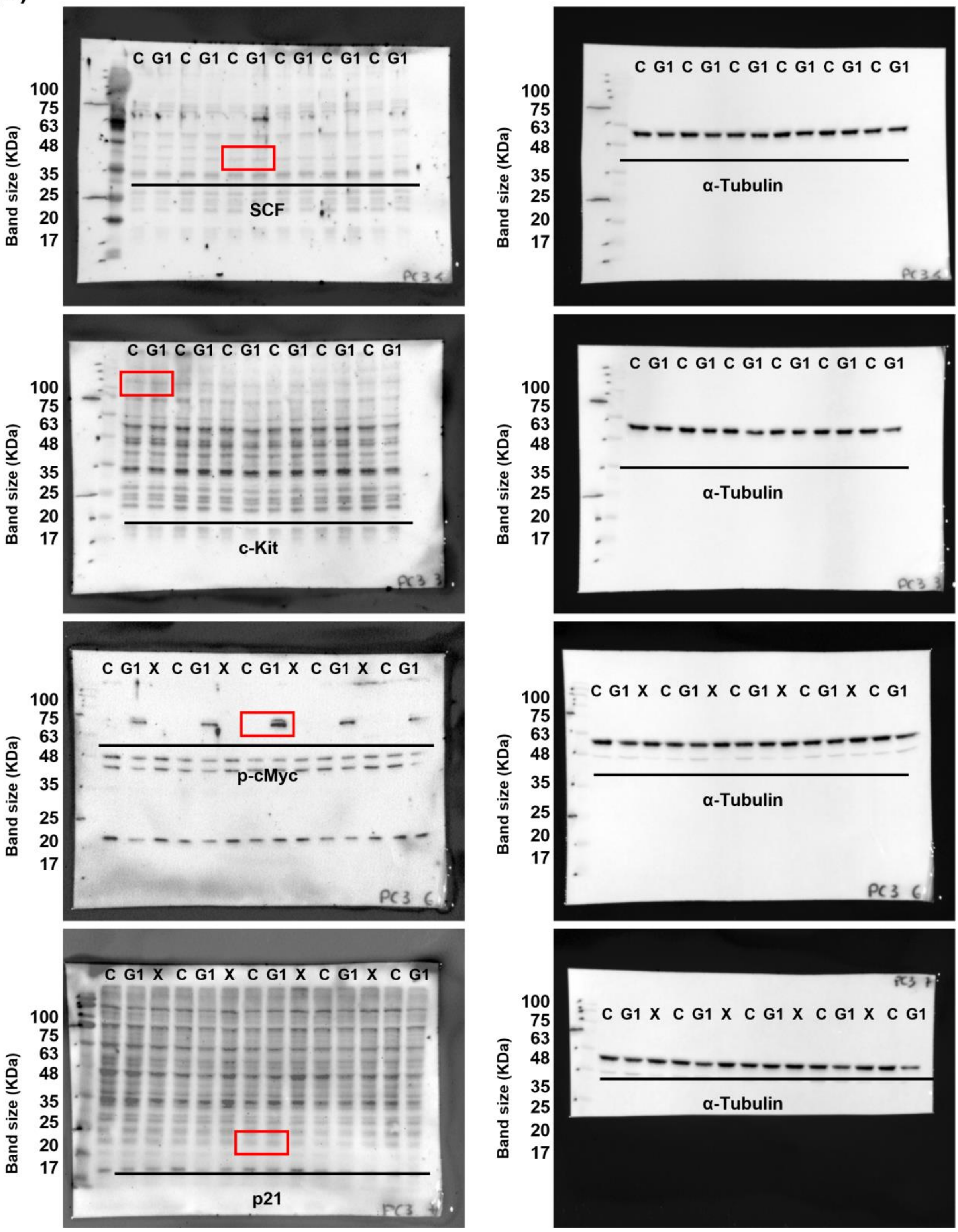

C G1 C G1 C G1 C G1 C G1 C G1
Band size (KDa)
100 75 63 48 35 25 20 17
SCF
α-Tubulin
c-Kit
C G1 X C G1 X C G1 X C G1 X C G1
p-cMyc
p21

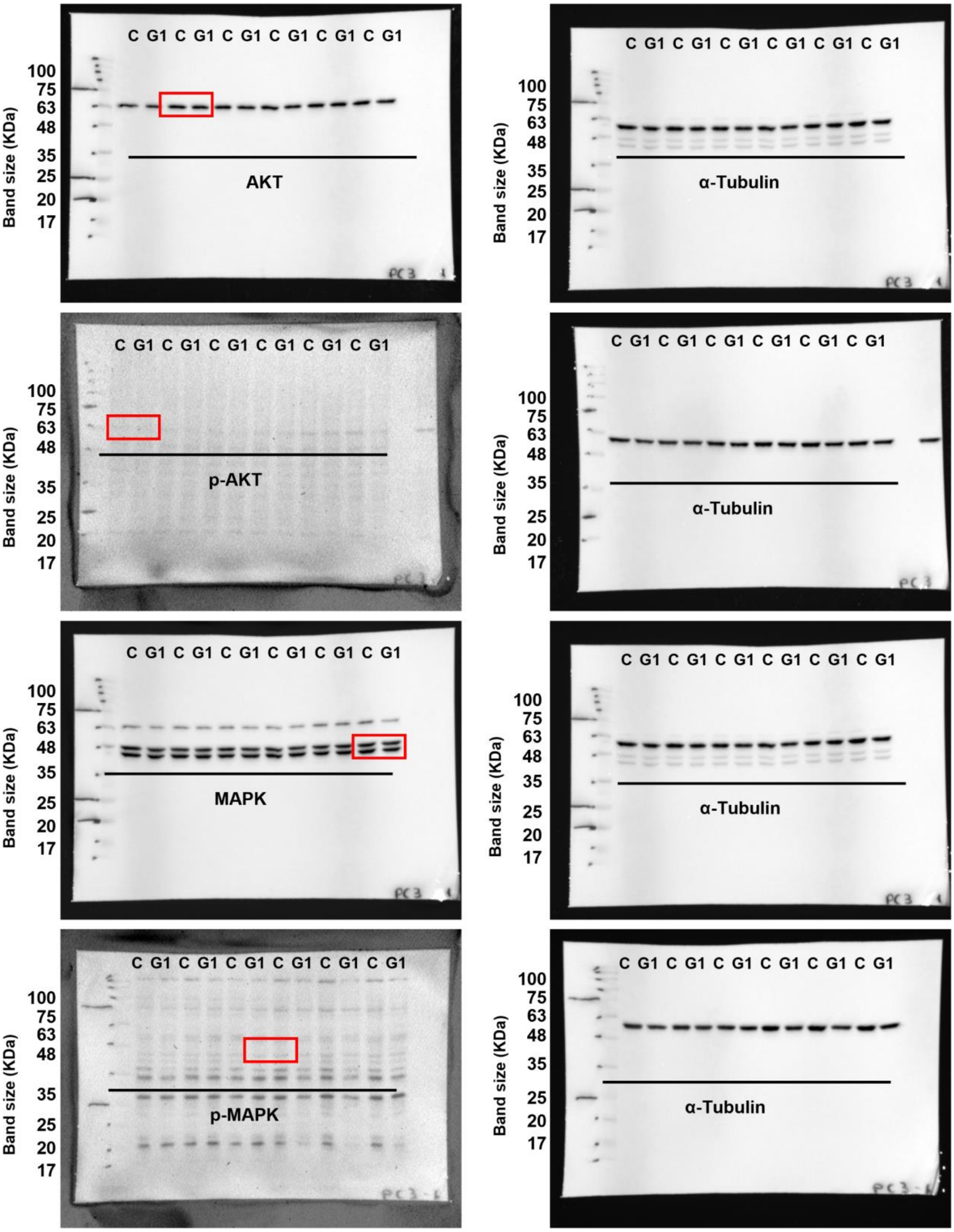

Figure S3. Original Western blots images after SCF, c-Kit, phospho c-Myc, p21, AKT, p-AKT, MAPK, p-MAPK and α-tubulin immunodetection in (A) LNCaP, (B) DU145 and (C) PC3 cells protein extracts after treatment with G1 for 24 h. Legend: C – control group; G1- G1-treated group; X – other samples analyzed. Red boxes represent the D pairs selected as representative to be shown in Figure 7.

Methodological notes:

Due to the presence of unspecific staining, to acquire the immunoblot images, some membranes were cut. Moreover, to remove the unspecific bands around 56 KDa, a soft stripping protocol was performed after SCF, c-Kit, phospho c-Myc, p21, p-AKT and p-MAPK detection and before incubating the membrane with the anti-α-tubulin antibody.

All the proteins blots represented were grouped by cell line and only one membrane of α-tubulin was shown in the manuscript.

Below in Figure S4 are provided the original images of the representative blots shown in the manuscript **Figure 8.**

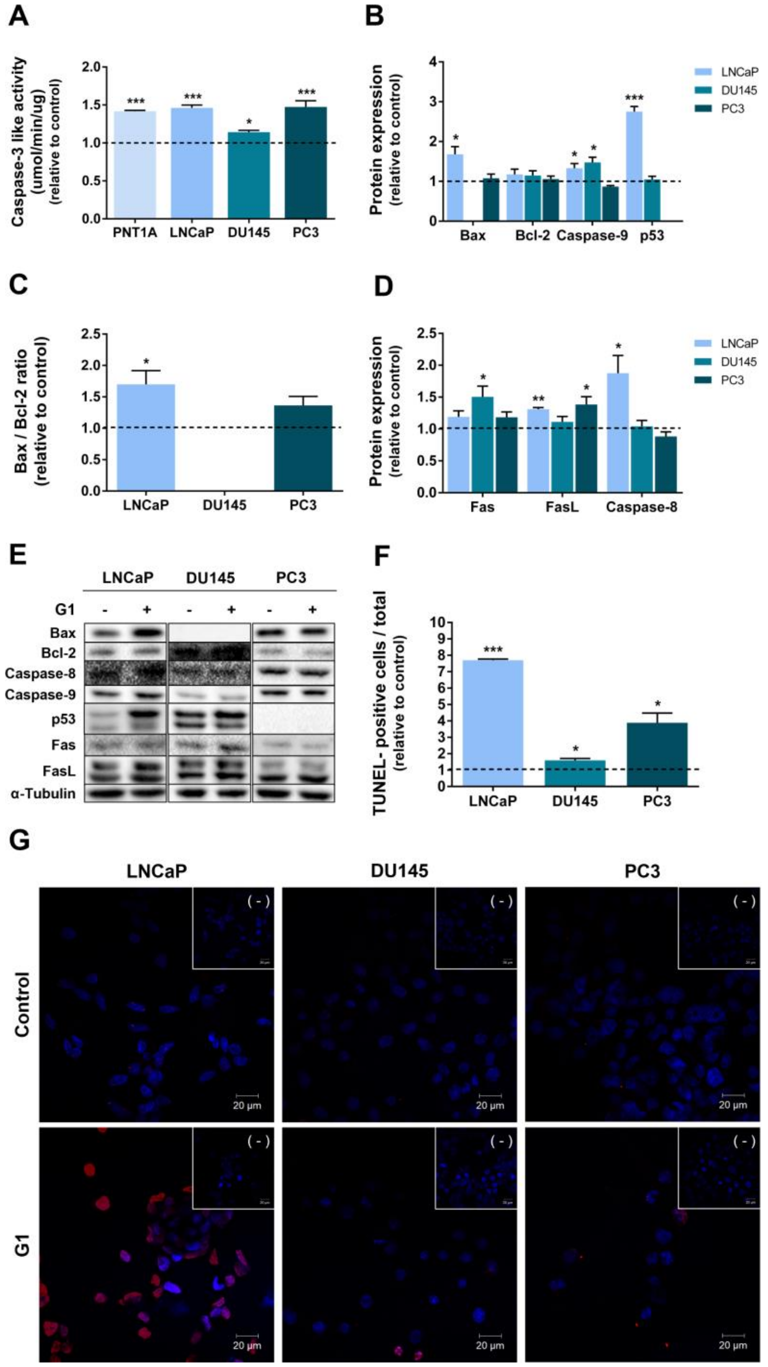

Figure 8. Effect of G1 in LNCaP, DU145 and PC3 human PCa cells apoptosis. Cells were treated with 1 µM G1 for 24 hours. **(A)** Caspase-3-like activity determined spectrophotometrically. **(B-D)** Protein expression of apoptosis regulators of the **(B, C)** intrinsic

and **(D)** extrinsic pathways of apoptosis determined by WB analysis after normalisation with α-tubulin. **(E)** Representative immunoblots. **(F)** Percentage of TUNEL-positive cells relative to the total cell number (Hoechst-stained nuclei). A total of 10 400 X magnification fields per slide was assessed. Results (**A-D, F)** are expressed as fold-change relative to the control untreated group (0 μM G1, dashed line). Error bars indicate mean ± S.E.M. (n=5) $*P < 0.05$ $**P < 0.01$ $***P < 0.001$. **(G)** Representative fluorescence microscopy images showing TUNEL labelling in control and G1-treated cells. Images were acquired using a Zeiss LSM 710 laser scanning confocal microscope (Carl Zeiss, Göttingen, Germany) under 400 X magnification. Nuclei are stained with Hoechst 33342 (blue) and TUNEL positive staining is red. Negative controls for TUNEL were performed following the manufacturer's instructions.

(A)

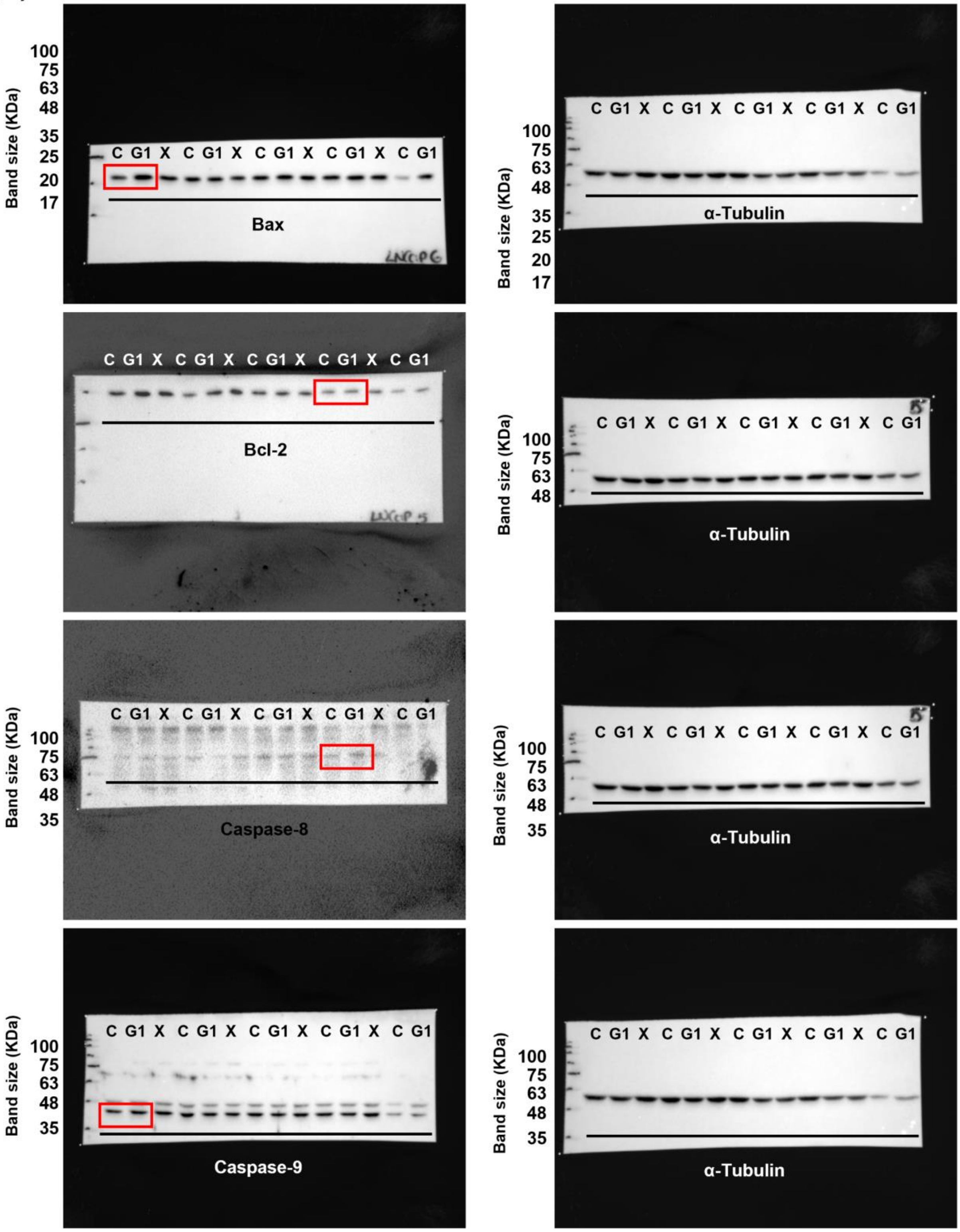
Band size (KDa)
100
75
63
48
35
25
20
17
C G1 X C G1 X C G1 X C G1 X C G1
Bax
α-Tubulin
Bcl-2
Caspase-8
Caspase-9

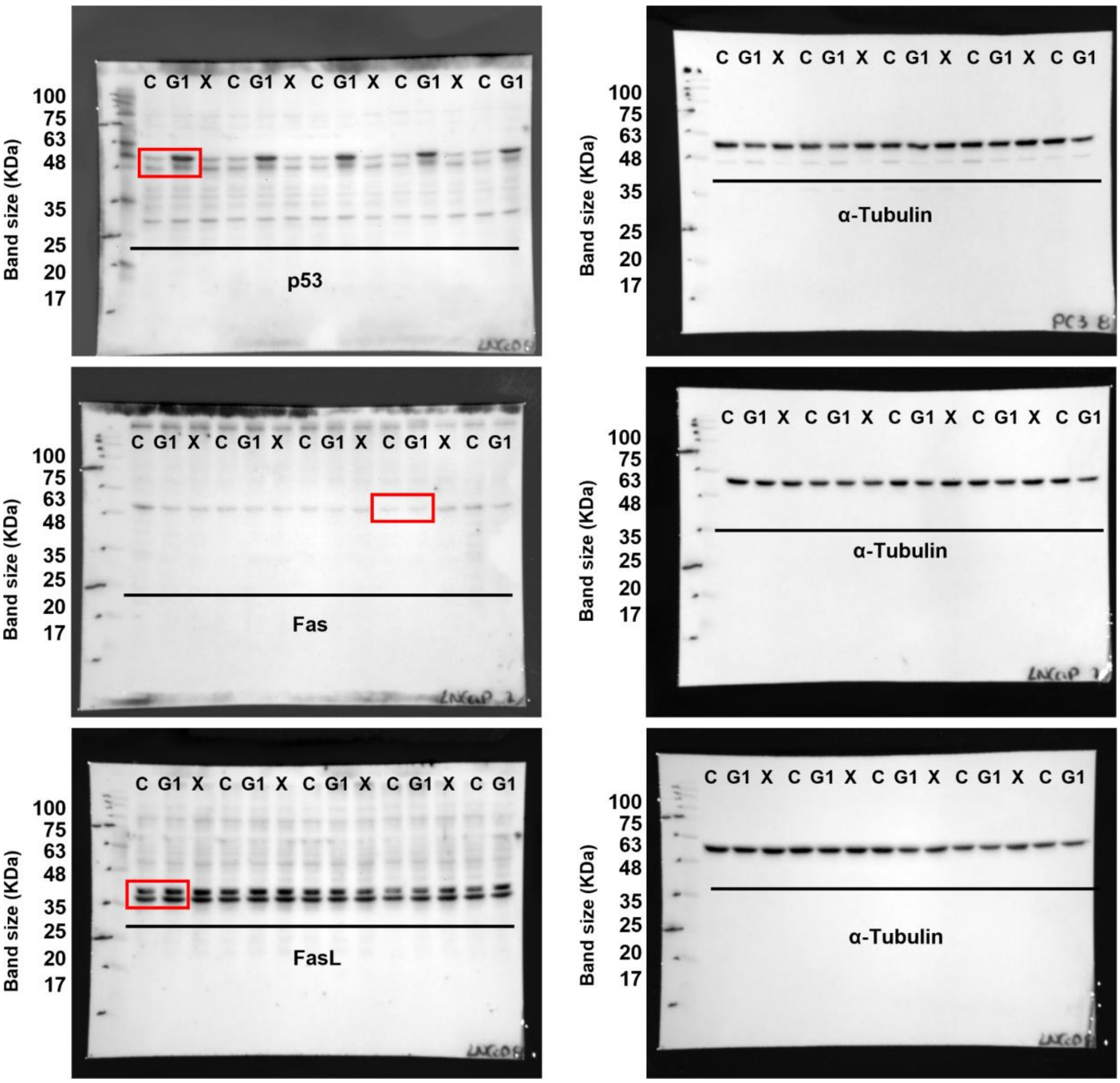
C G1 X C G1 X C G1 X C G1 X C G1
Band size (KDa)
100
75
63
48
35
25
20
17
p53
α-Tubulin
Fas
FasL

(B)

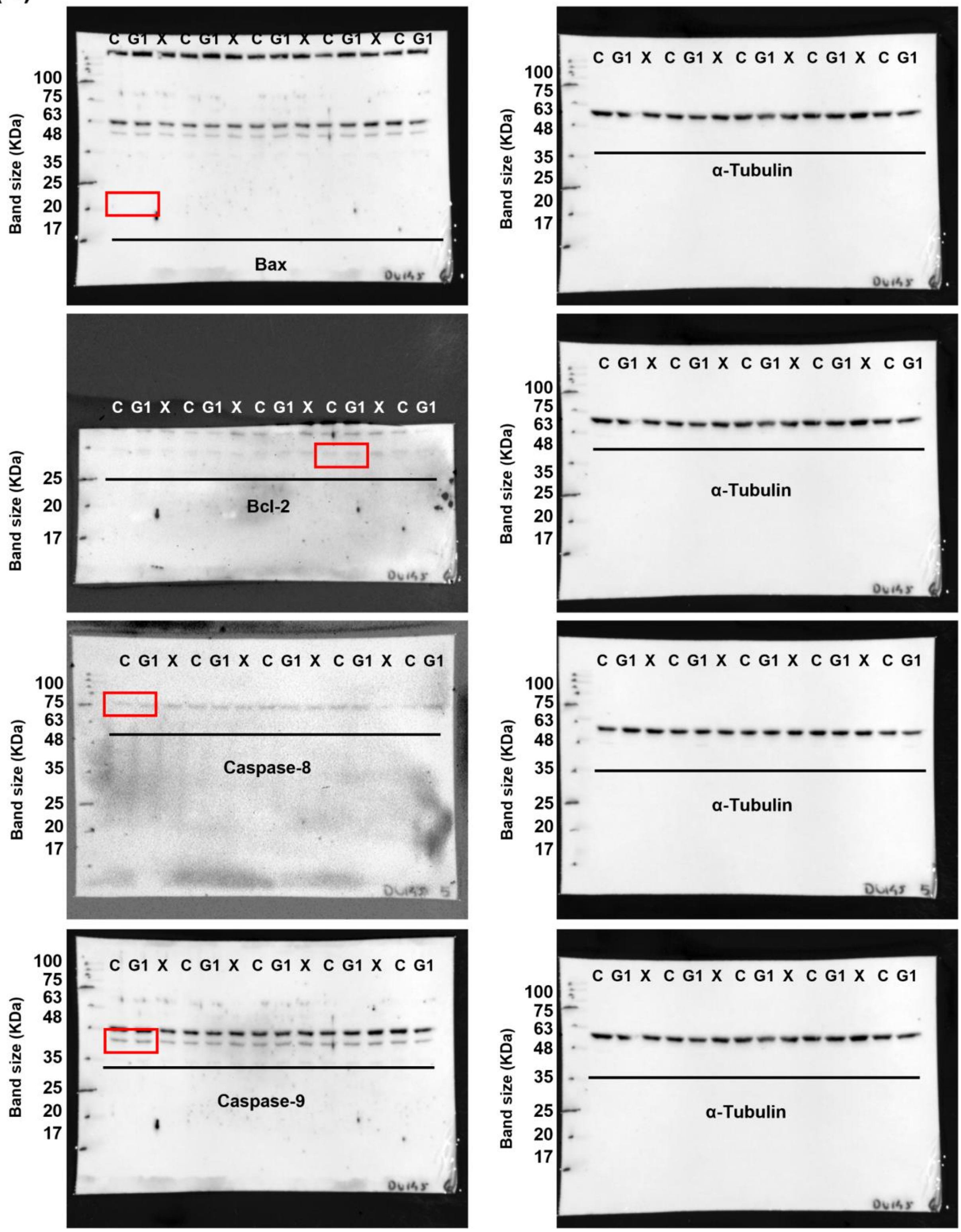
Band size (KDa)
100
75
63
48
35
25
20
17
C G1 X C G1 X C G1 X C G1 X C G1
Bax
α-Tubulin
Bcl-2
Caspase-8
Caspase-9

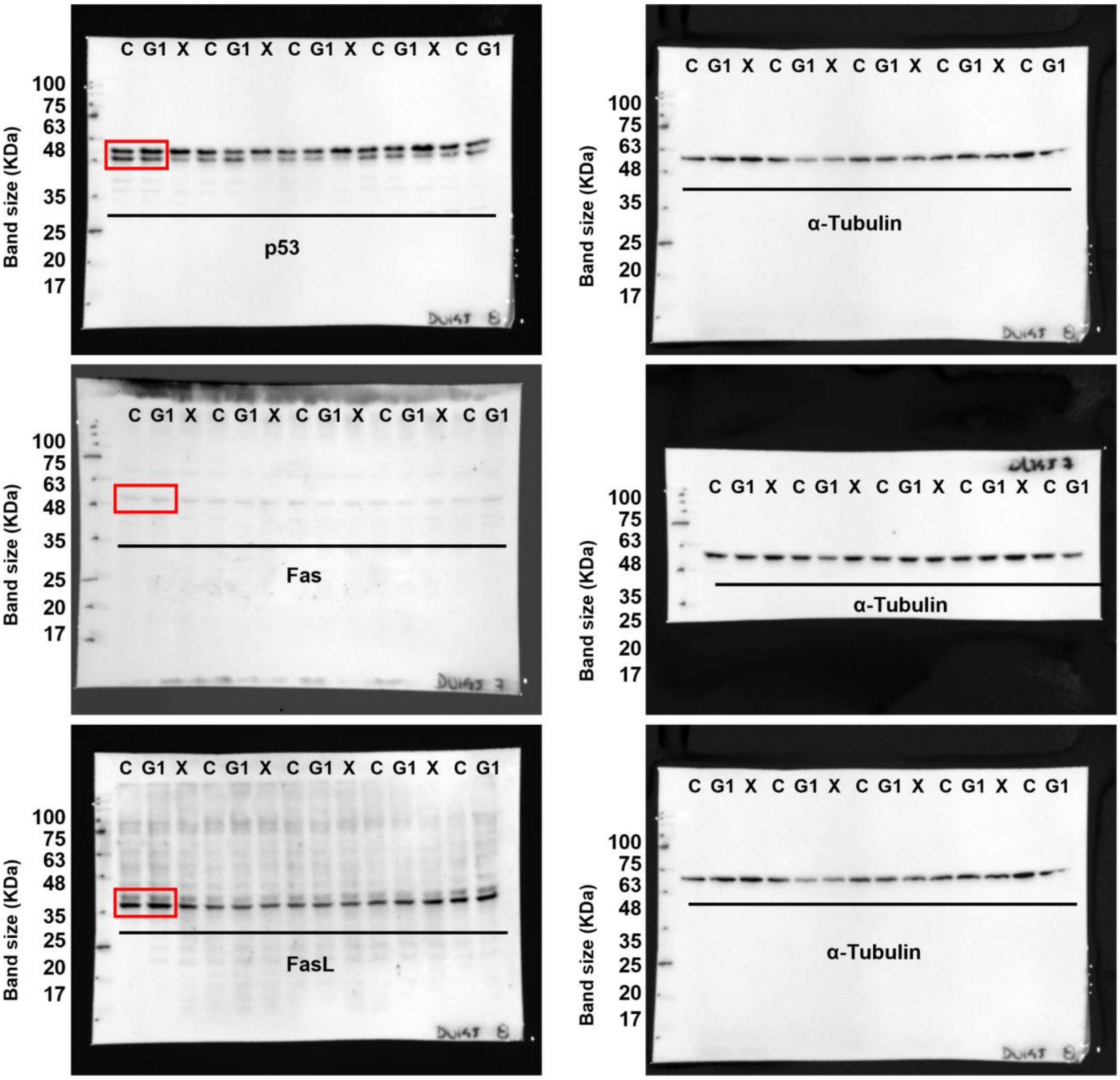
C G1 X C G1 X C G1 X C G1 X C G1
Band size (KDa)
100
75
63
48
35
25
20
17
p53
α-Tubulin
Fas
FasL
DU145 8
DU145 7

(C)

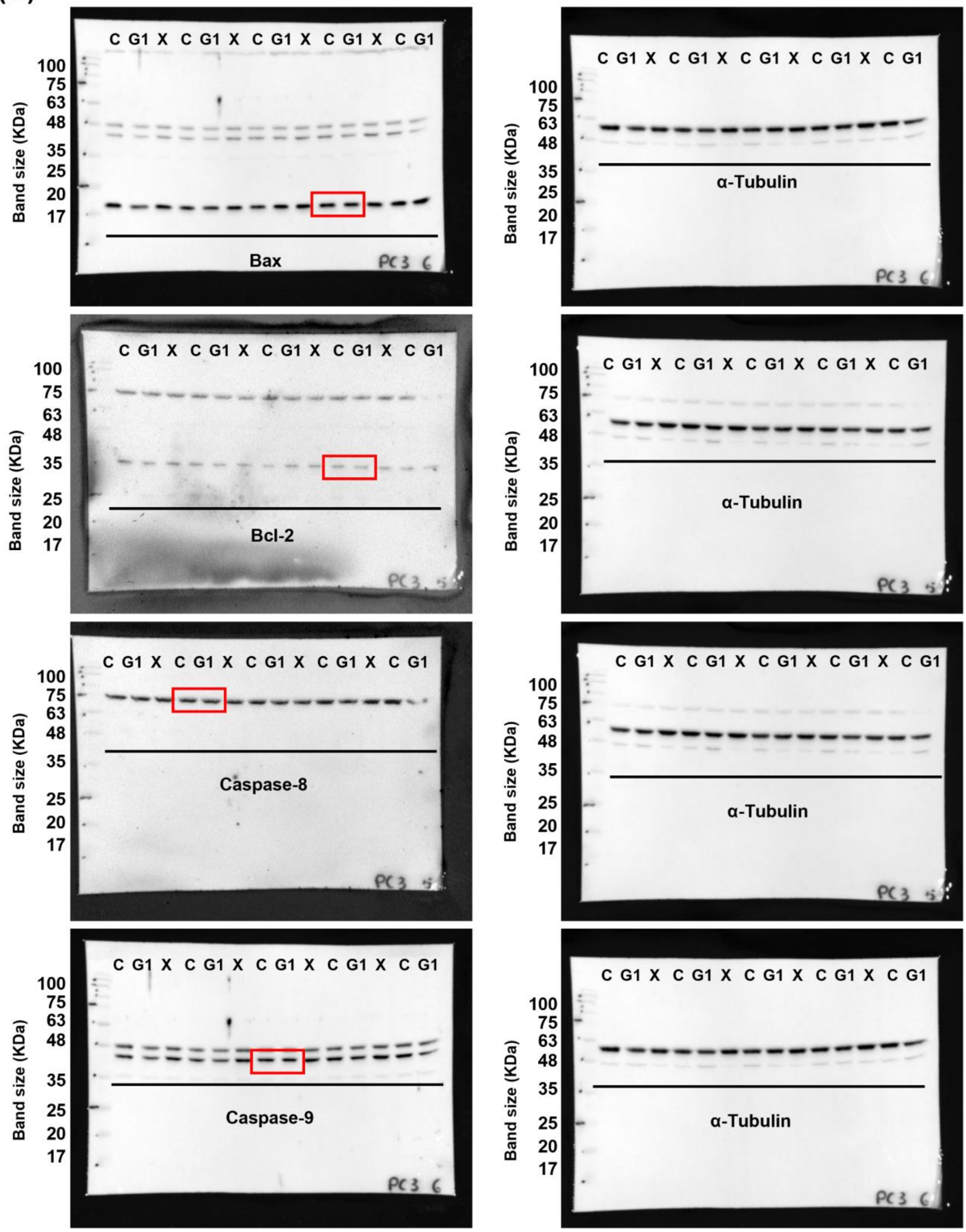
C G1 X C G1 X C G1 X C G1 X C G1
Band size (KDa)
100
75
63
48
35
25
20
17
Bax
Bcl-2
Caspase-8
Caspase-9
α-Tubulin
PC3 6
PC3 5

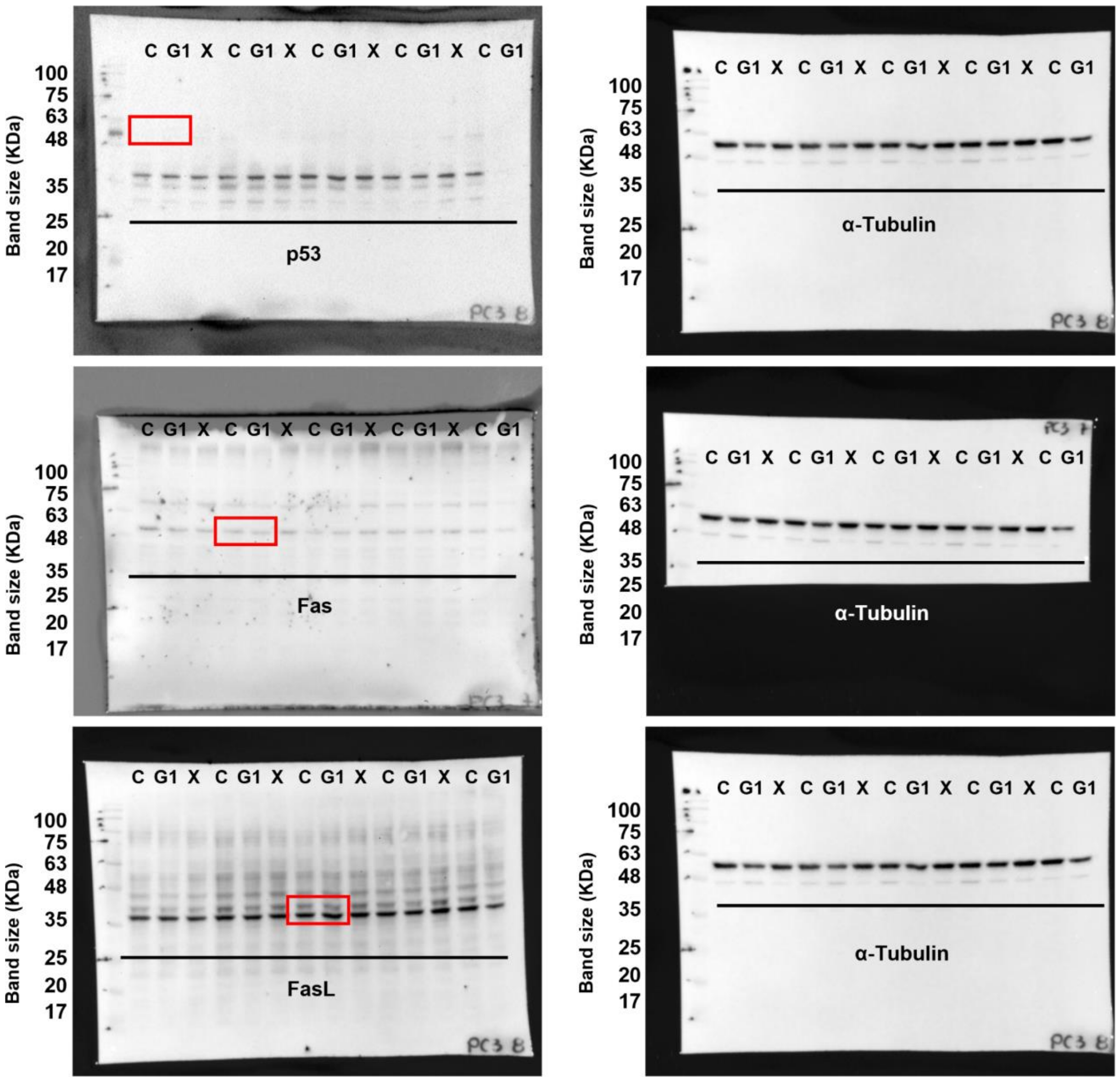

Figure S4. Original Western blots images after Bax, Bcl-2, Caspase-8, Caspae-9, p53, Fas, FasL and α-tubulin immunodetection in (A) LNCaP, (B) DU145 and (C) PC3 cells protein extracts after treatment with G1 for 24 h. Legend: C – control group; G1- G1-treated group; X – other samples analyzed. Red boxes represent the E pairs selected as representative to be shown in Figure 8.

Methodological notes:

Due to the presence of unspecific staining, to acquire the immunoblot images, some membranes were cut. Moreover, to remove the unspecific bands around 56 KDa, a soft stripping protocol was performed after p53, Fas and FasL detection and before incubating the membrane with the anti-α-tubulin antibody.

All the proteins blots represented were grouped by cell line and only one membrane of α-tubulin was shown in the manuscript.

Below in Figure S5 are provided the original images of the representative blots shown in the manuscript **Figure 10.**

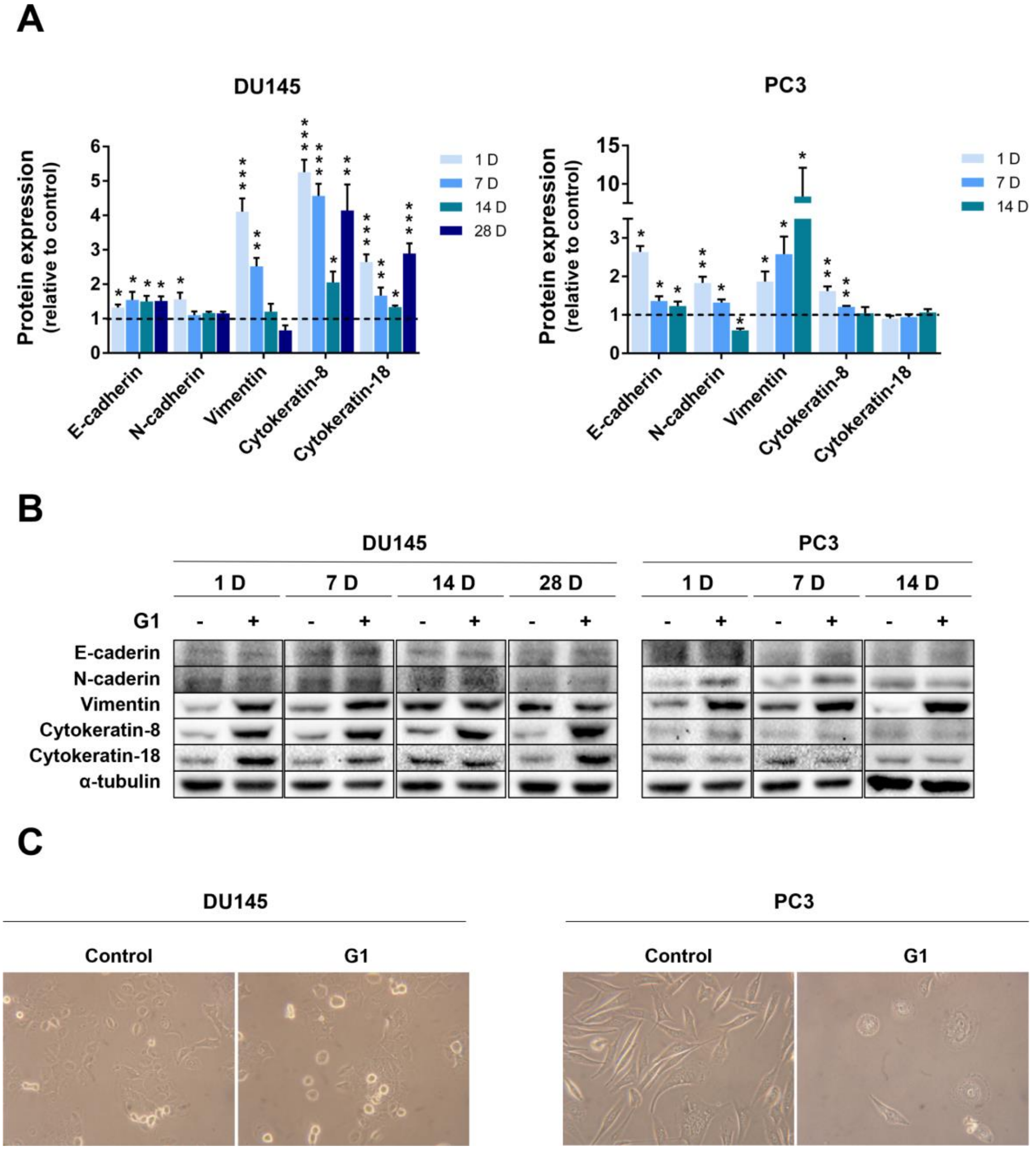

Figure 10. G1 effects in modulating the expression of epithelial and mesenchymal markers in DU145 and PC3 human PCa cells in long-term culture. Cells were treated with 1 μM G1 for 1 to 28 days (1-28 D). **(A)** Protein expression determined by WB analysis after normalization with α-tubulin. Results are expressed as fold-change relative to the control untreated group (0 μM G1, dashed line). Error bars indicate mean ± S.E.M. (n=5) *$P < 0.05$ **$P < 0.01$ ***$P < 0.001$. **(B)** Representative immunoblots. **(C)** Representative images of DU145 and PC3 cells morphology after 28 D stimulation with G1 compared with the control. Images were obtained in optical microscope under 200 X magnification.

(A)

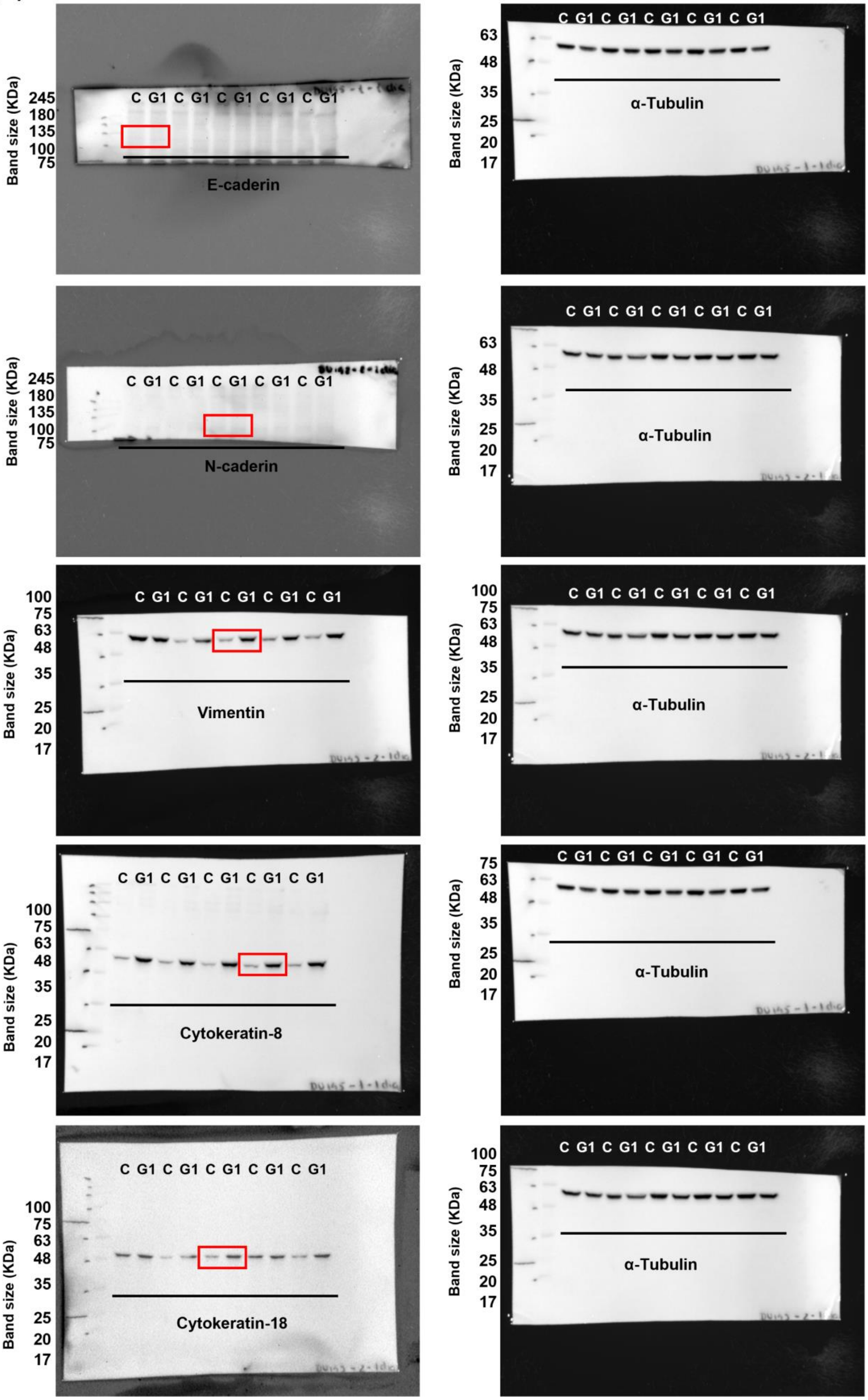
Band size (KDa)
245
180
135
100
75
C G1 C G1 C G1 C G1 C G1
E-caderin
63
48
35
25
20
17
α-Tubulin
N-caderin
α-Tubulin
100
75
Vimentin
α-Tubulin
Cytokeratin-8
α-Tubulin
Cytokeratin-18
α-Tubulin

(B)

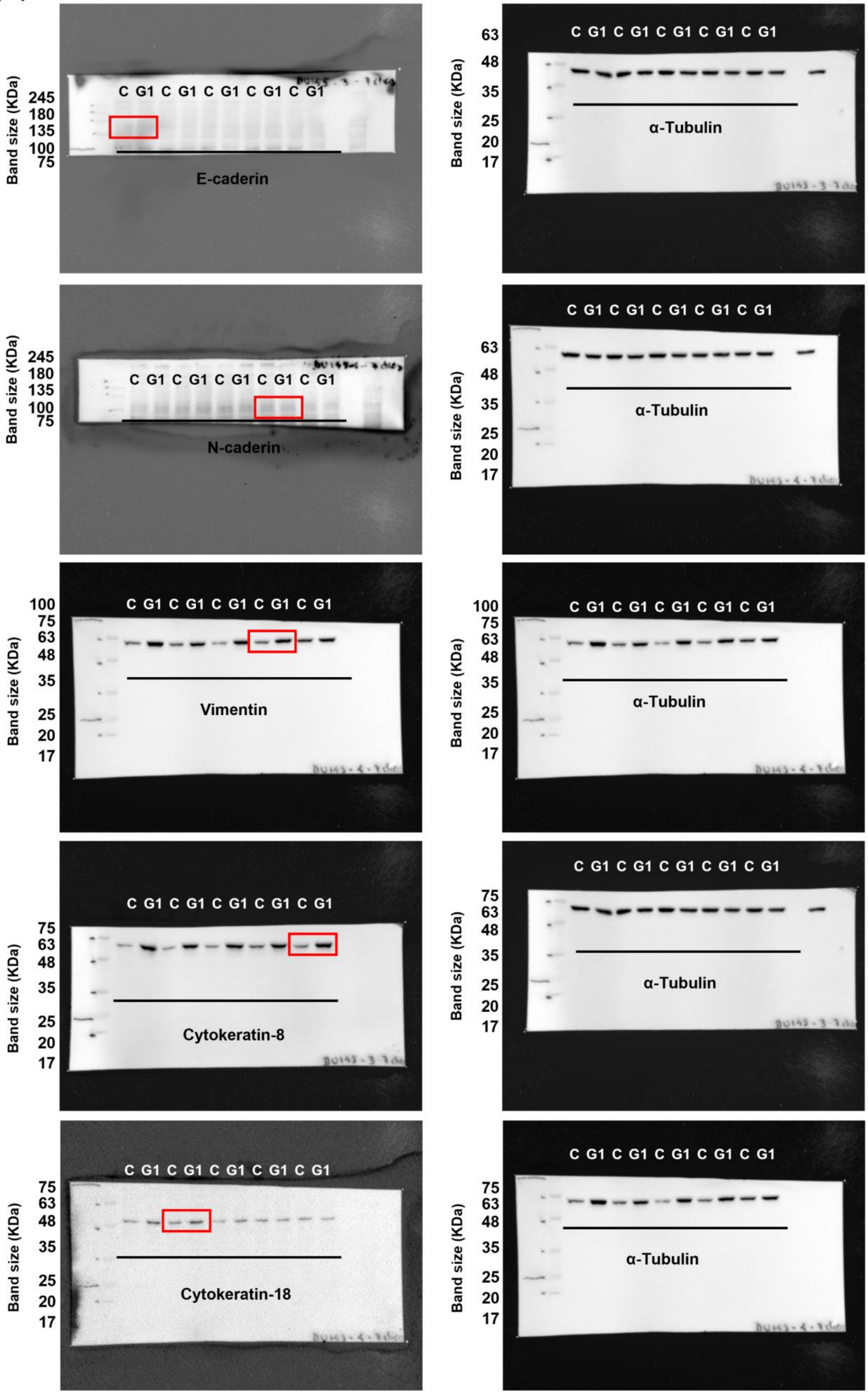
C G1 C G1 C G1 C G1 C G1
Band size (KDa)
245
180
135
100
75
E-caderin
63
48
35
25
20
17
α-Tubulin
N-caderin
Vimentin
Cytokeratin-8
Cytokeratin-18

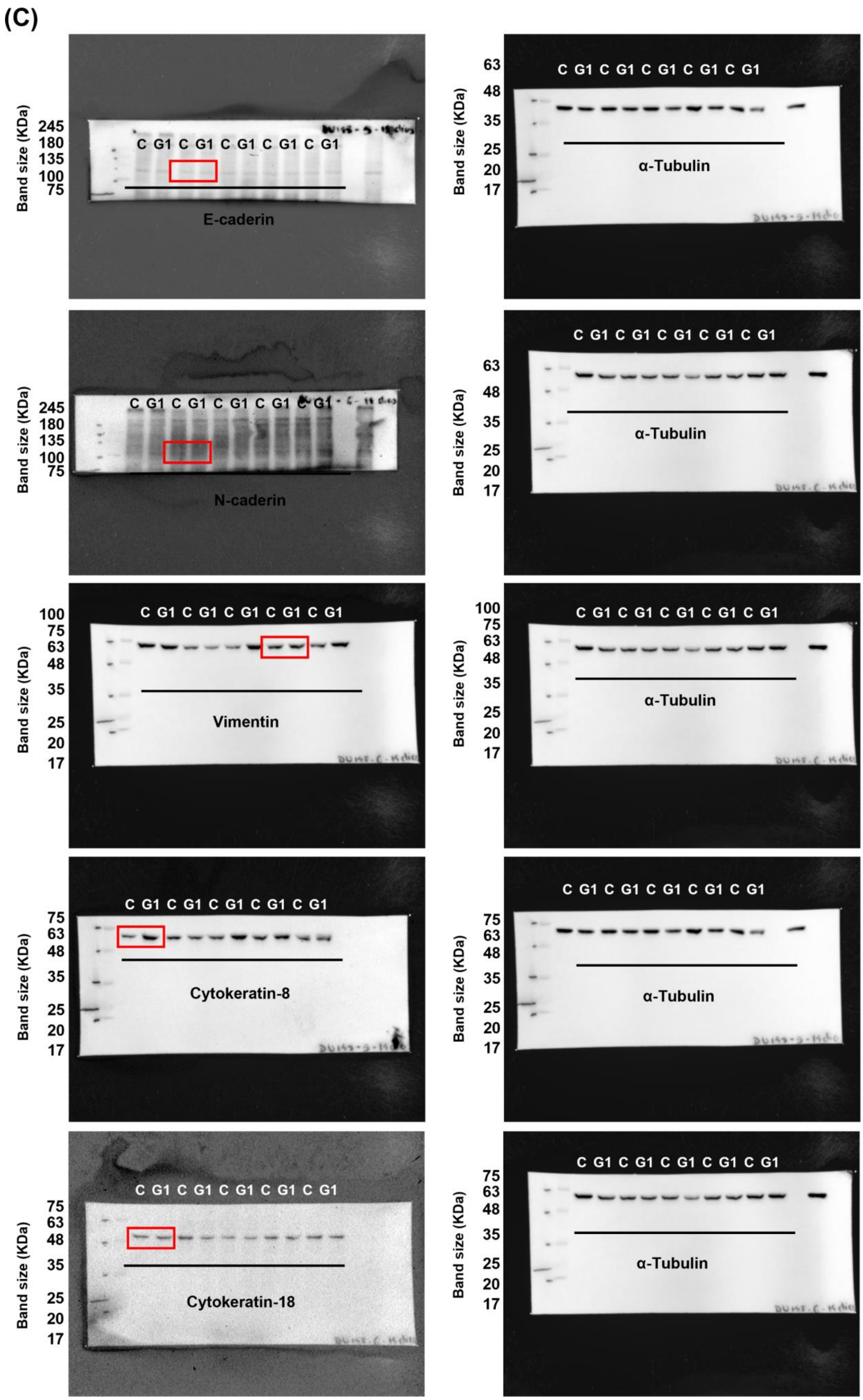
(C)
Band size (KDa)
245
180
135
100
75
C G1 C G1 C G1 C G1 C G1
E-caderin
63
48
35
25
20
17
α-Tubulin
N-caderin
Vimentin
Cytokeratin-8
Cytokeratin-18

(D)

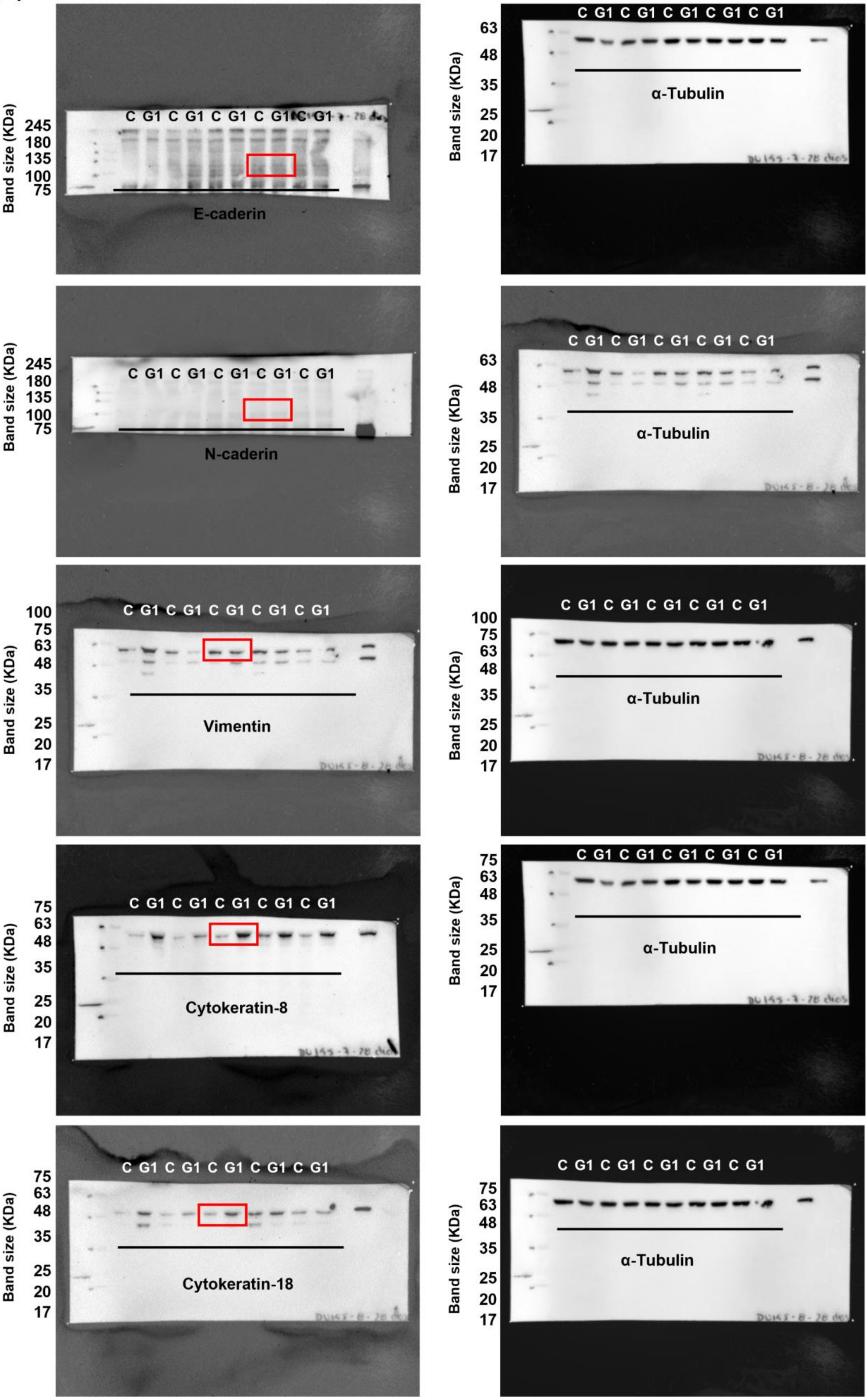
C G1 C G1 C G1 C G1 C G1
Band size (KDa)
245
180
135
100
75
E-caderin
α-Tubulin
N-caderin
Vimentin
Cytokeratin-8
Cytokeratin-18
63
48
35
25
20
17

(E)

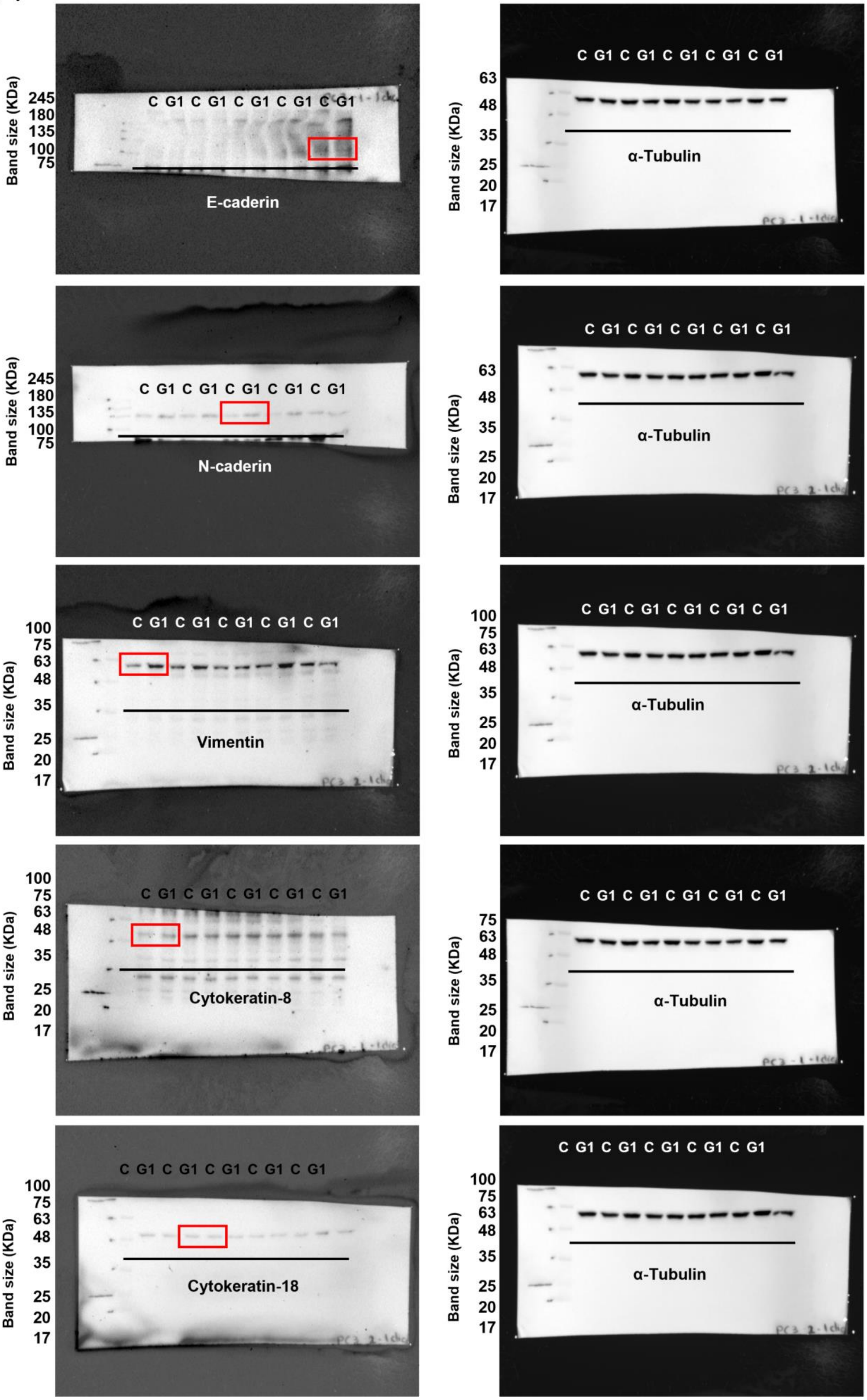

Band size (KDa)
245
180
135
100
75
C G1 C G1 C G1 C G1 C G1
E-caderin
63
48
35
25
20
17
α-Tubulin
N-caderin
Vimentin
Cytokeratin-8
Cytokeratin-18

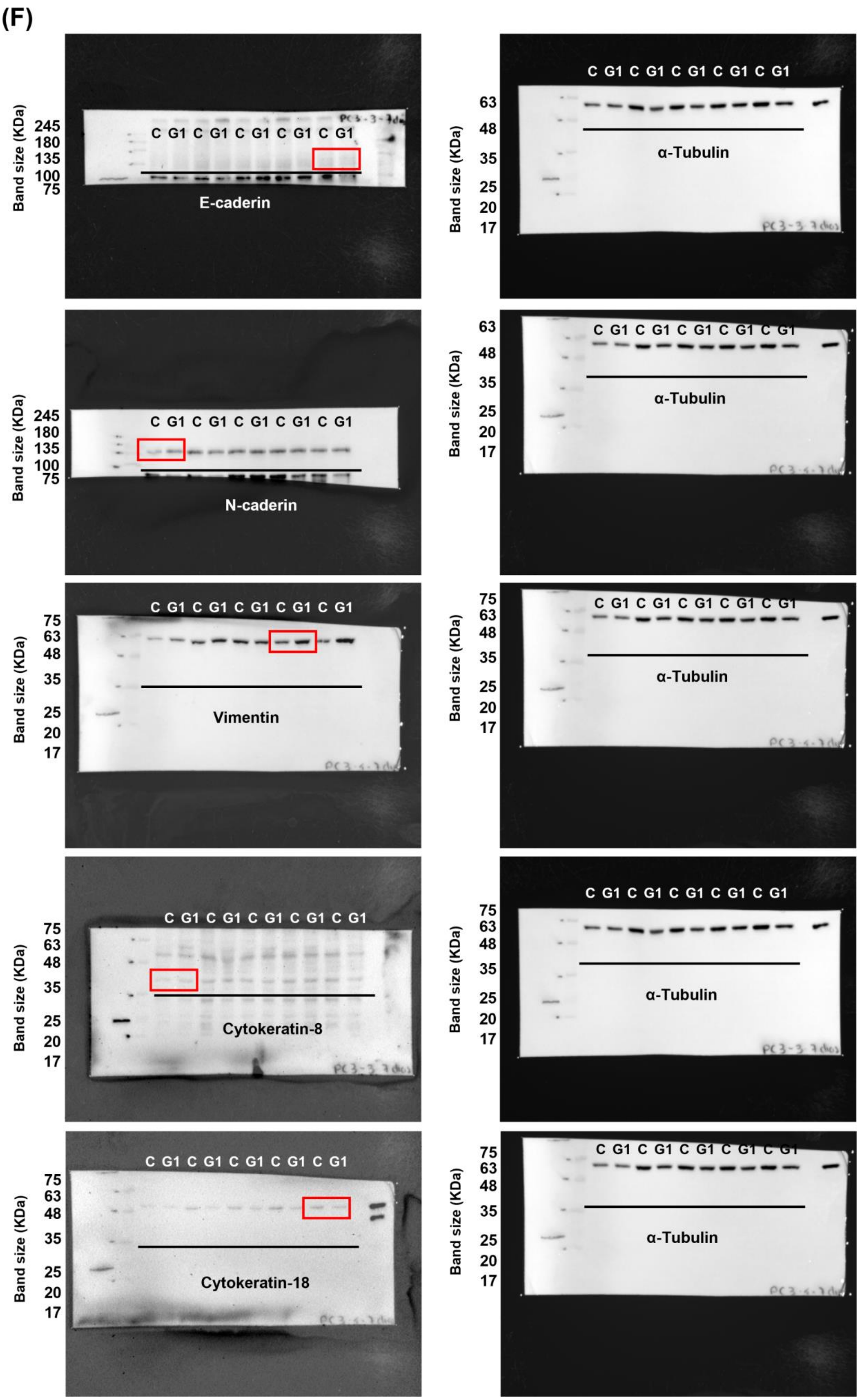
(F)
Band size (KDa)
245
180
135
100
75
C G1 C G1 C G1 C G1 C G1
E-caderin
63
48
35
25
20
17
α-Tubulin
N-caderin
Vimentin
Cytokeratin-8
Cytokeratin-18

(G)

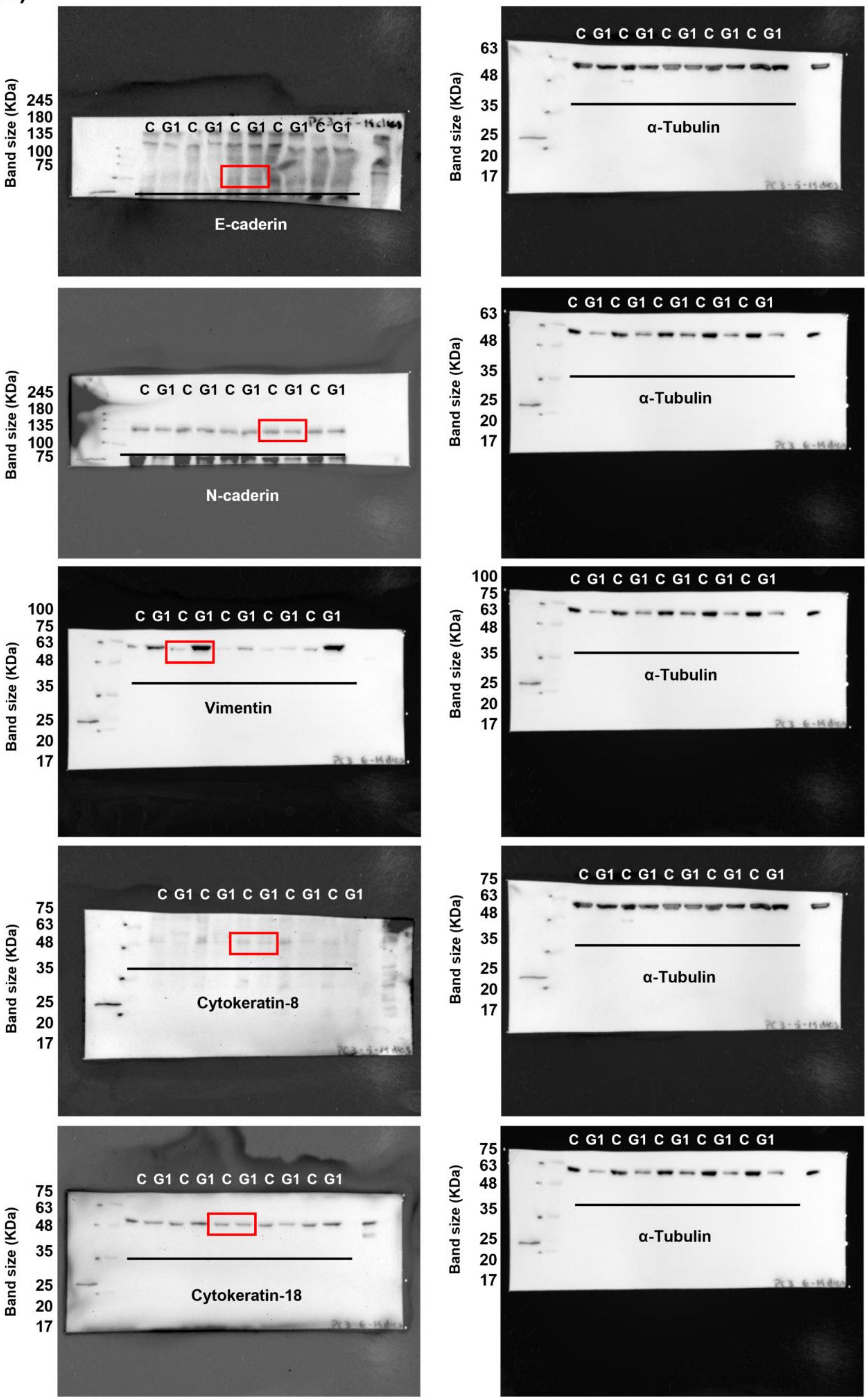

C G1 C G1 C G1 C G1 C G1
Band size (KDa)
245
180
135
100
75
E-caderin
63
48
35
25
20
17
α-Tubulin
N-caderin
Vimentin
Cytokeratin-8
Cytokeratin-18

Figure S5. Original Western blots images after E-caderin, N-caderin, Vimentin, Cytokeratin-8, Cytokeratin-18 and α-tubulin immuno-detection after treatment with G1 for (A) 1, (B) 7, (C) 14 and (D) 28 days in DU145 and for (E) 1, (F) 7 and (G) 14 days in PC3 cells protein extracts. Legend: C – control group; G1- G1-treated group. Red boxes represent the B pairs selected as representative to be shown in Figure 10.

Methodological notes:

In Figure S5, as seen in the right panel by the α-tubulin labelling, the amount of protein loaded in the gel was not always the same, which occurred due to limitations in the amount of protein extract.

Due to the presence of unspecific staining, to acquire the immunoblot images, some membranes were cut. Moreover, to remove the unspecific bands around 56 KDa, a soft stripping protocol was performed after Vimentin, Cytokeratin-8 and Cytokeratin-18 detection and before incubating the membrane with the anti-α-tubulin antibody.

All the proteins blots represented were grouped by cell line and only one membrane of α-tubulin was shown in the manuscript.

Below in Figure S6 are provided the original images of the representative blots shown in the manuscript **Figure 11.**

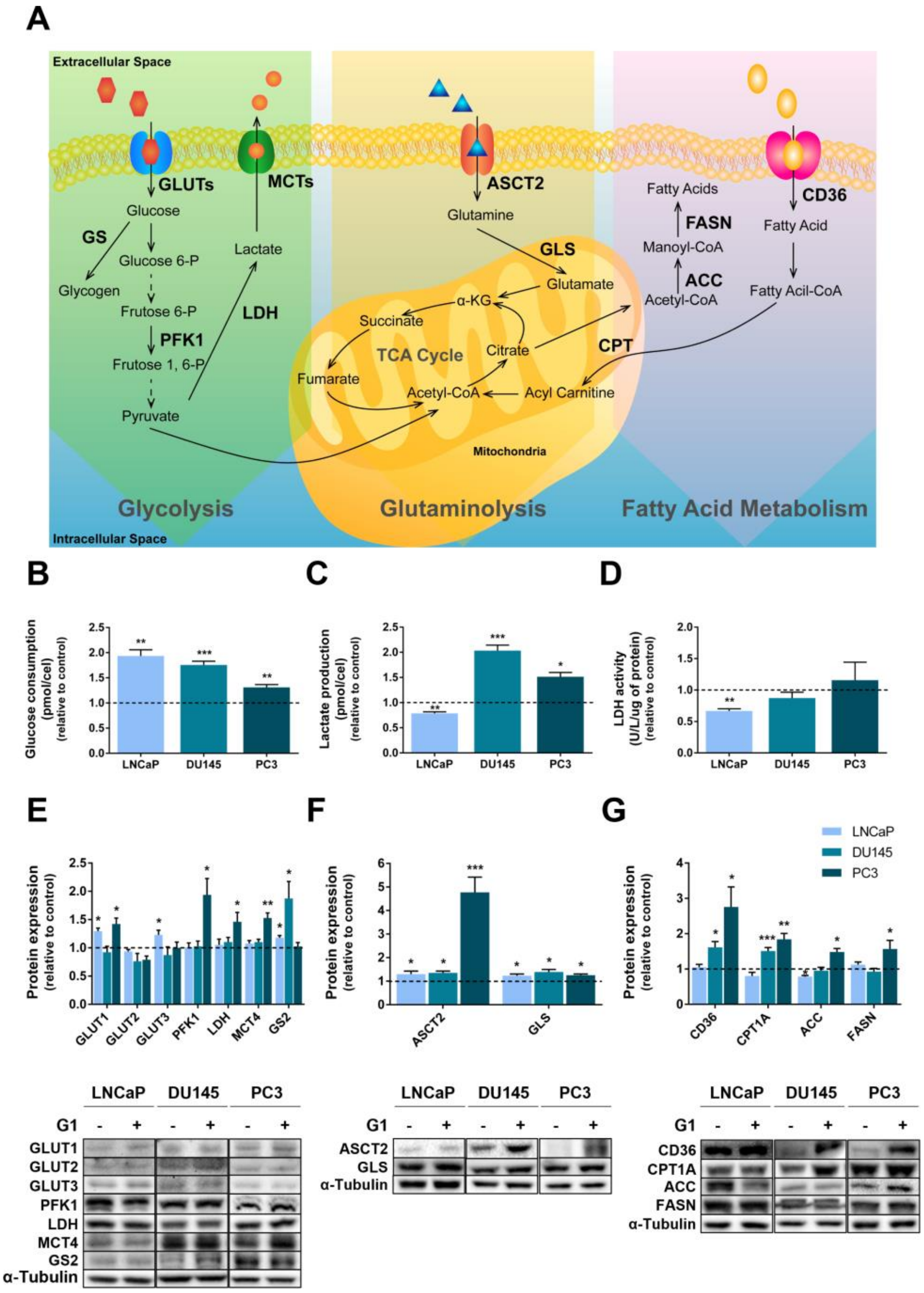

Figure 11 G1 actions in modulating the metabolic profile of LNCaP, DU145 and PC3 human PCa cells. Cells were treated with 1 μM G1 for 24 hours. **(A)** Schematic representation of glycolysis, glutaminolysis and fatty acid metabolism. Glucose enters the cell through the activity of GLUTs, being metabolized through glycolytic enzymes, namely PFK1. Pyruvate, the end product of glycolysis, can be driven to mitochondria or converted into lactate by LDH. MCT4 at the cell membrane exports lactate to the extracellular space. Alternatively, glucose can be converted into glycogen through GS. Glutamine is incorporated into the cell by the membrane transporter ASCT2 and is converted to glutamate by the mitochondrial GLS. CD36 transporter is responsible for the uptake of fatty acids that can be incorporated into mitochondria by CPT1A, undergoing β-oxidation. ACC and FASN are the target players in fatty acid *de novo* synthesis from citrate. **(B)** Glucose consumption and **(C)** Lactate production determined by spectrophotometric measurement of glucose and lactate content in the extracellular medium. **(D)** LDH activity determined spectrophotometrically. **(E-G)** Protein expression of target players in **(E)** glycolytic metabolism **(F)** glutamine metabolism, and **(G)** lipid handling determined by WB analysis after normalization with α-tubulin. Results are expressed as fold-change relative to the control untreated group (0 μM G1, dashed line). Error bars indicate mean ± S.E.M. (n=5) *$P < 0.05$ **$P < 0.01$ ***$P < 0.001$. Representative immunoblots are shown as bottom panels.

(A)

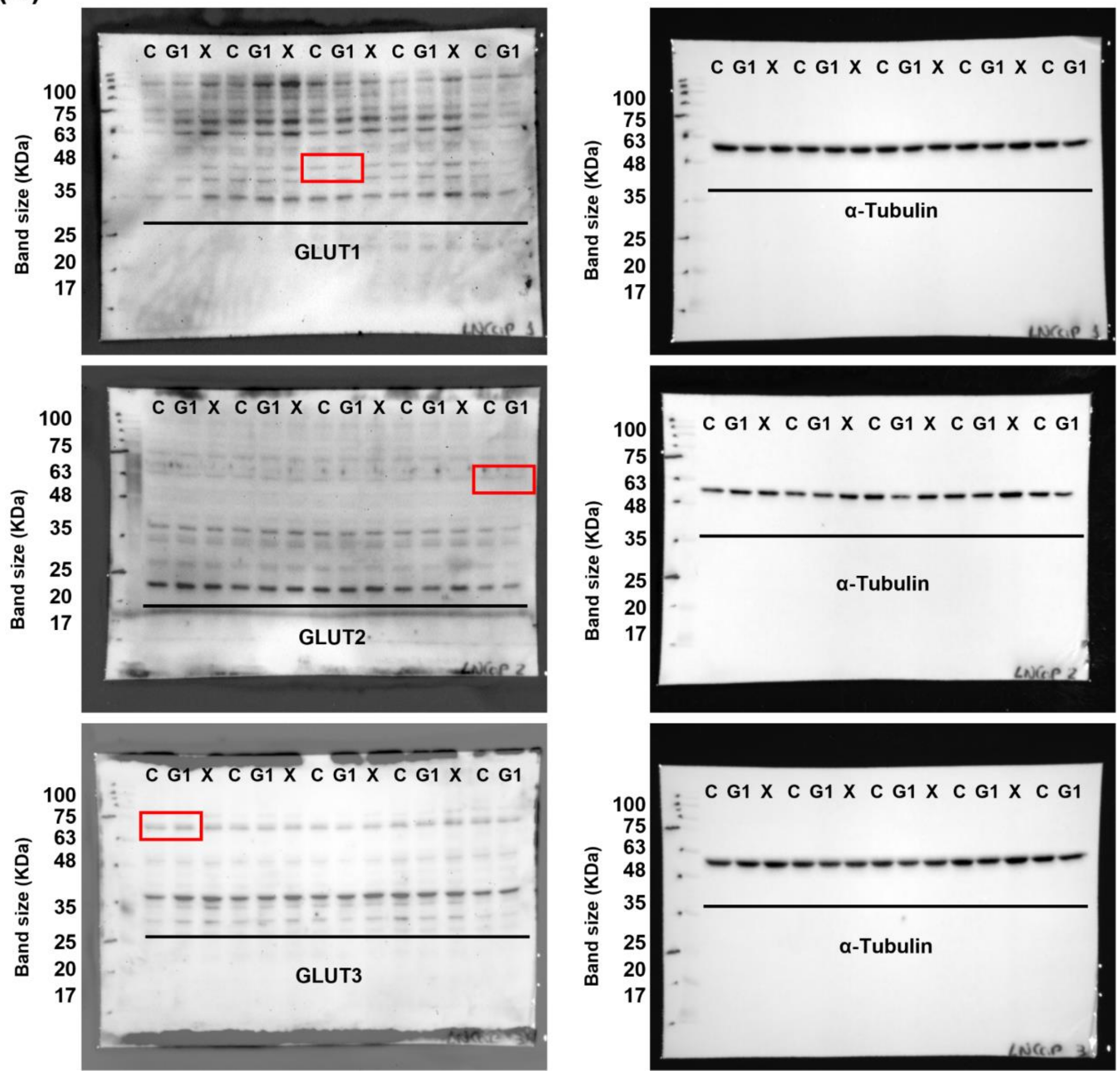
C G1 X C G1 X C G1 X C G1 X C G1
Band size (KDa)
100
75
63
48
35
25
20
17
GLUT1
α-Tubulin
GLUT2
GLUT3

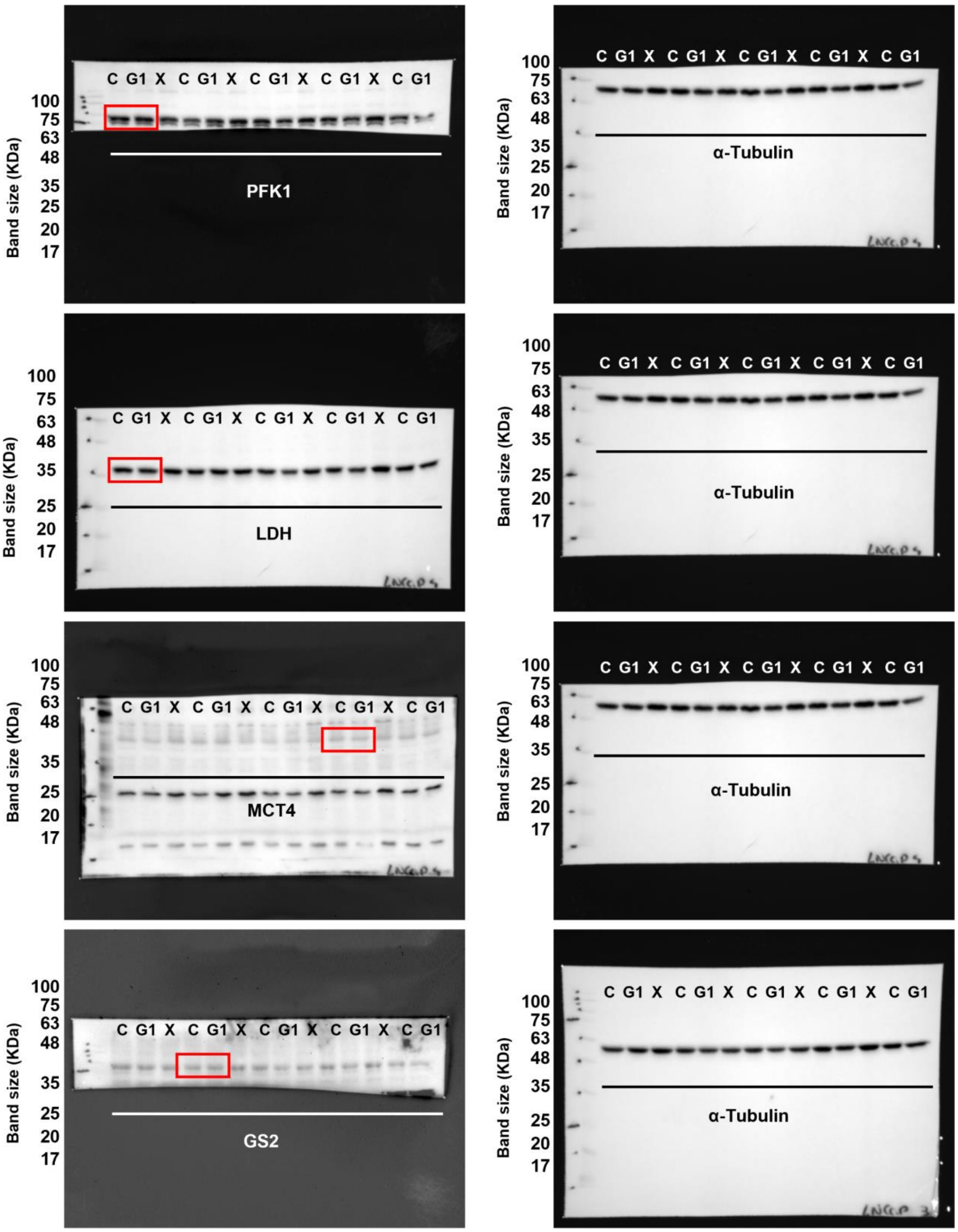
PFK1
LDH
MCT4
GS2
α-Tubulin
α-Tubulin
α-Tubulin
α-Tubulin
Band size (KDa)
100
75
63
48
35
25
20
17
C G1 X C G1 X C G1 X C G1 X C G1

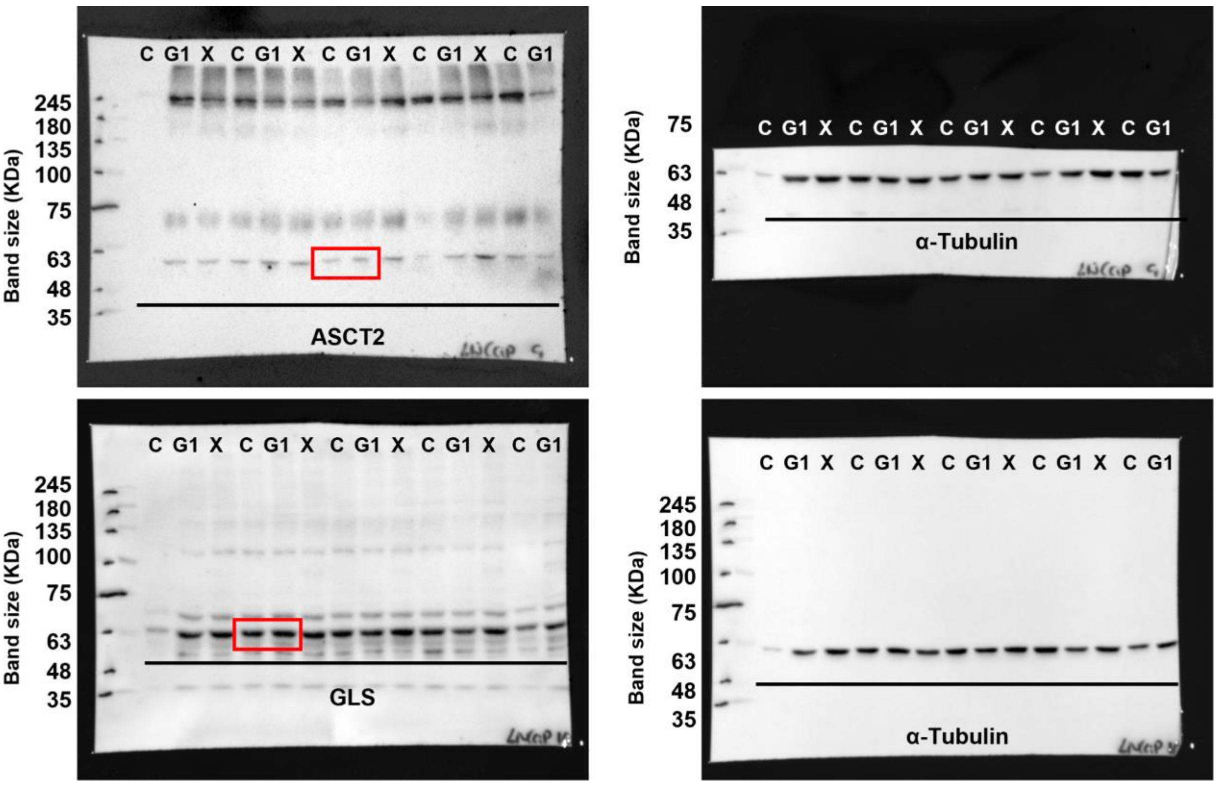

C G1 X C G1 X C G1 X C G1 X C G1
245
180
135
100
75
63
48
35
Band size (KDa)
ASCT2
C G1 X C G1 X C G1 X C G1 X C G1
75
63
48
35
Band size (KDa)
α-Tubulin
C G1 X C G1 X C G1 X C G1 X C G1
245
180
135
100
75
63
48
35
Band size (KDa)
GLS
C G1 X C G1 X C G1 X C G1 X C G1
245
180
135
100
75
63
48
35
Band size (KDa)
α-Tubulin

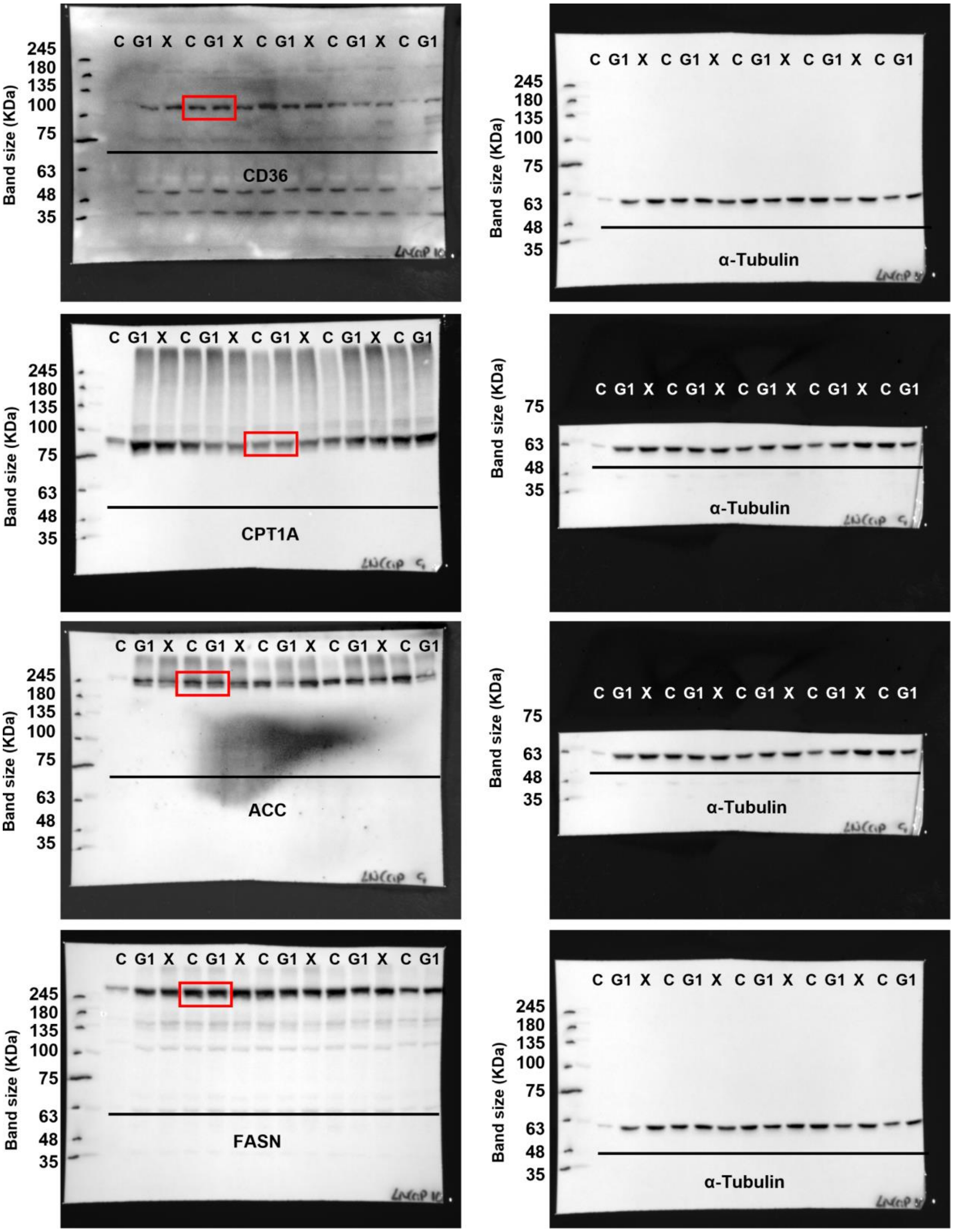
C G1 X C G1 X C G1 X C G1 X C G1
Band size (KDa)
245
180
135
100
75
63
48
35
CD36
α-Tubulin
CPT1A
ACC
FASN

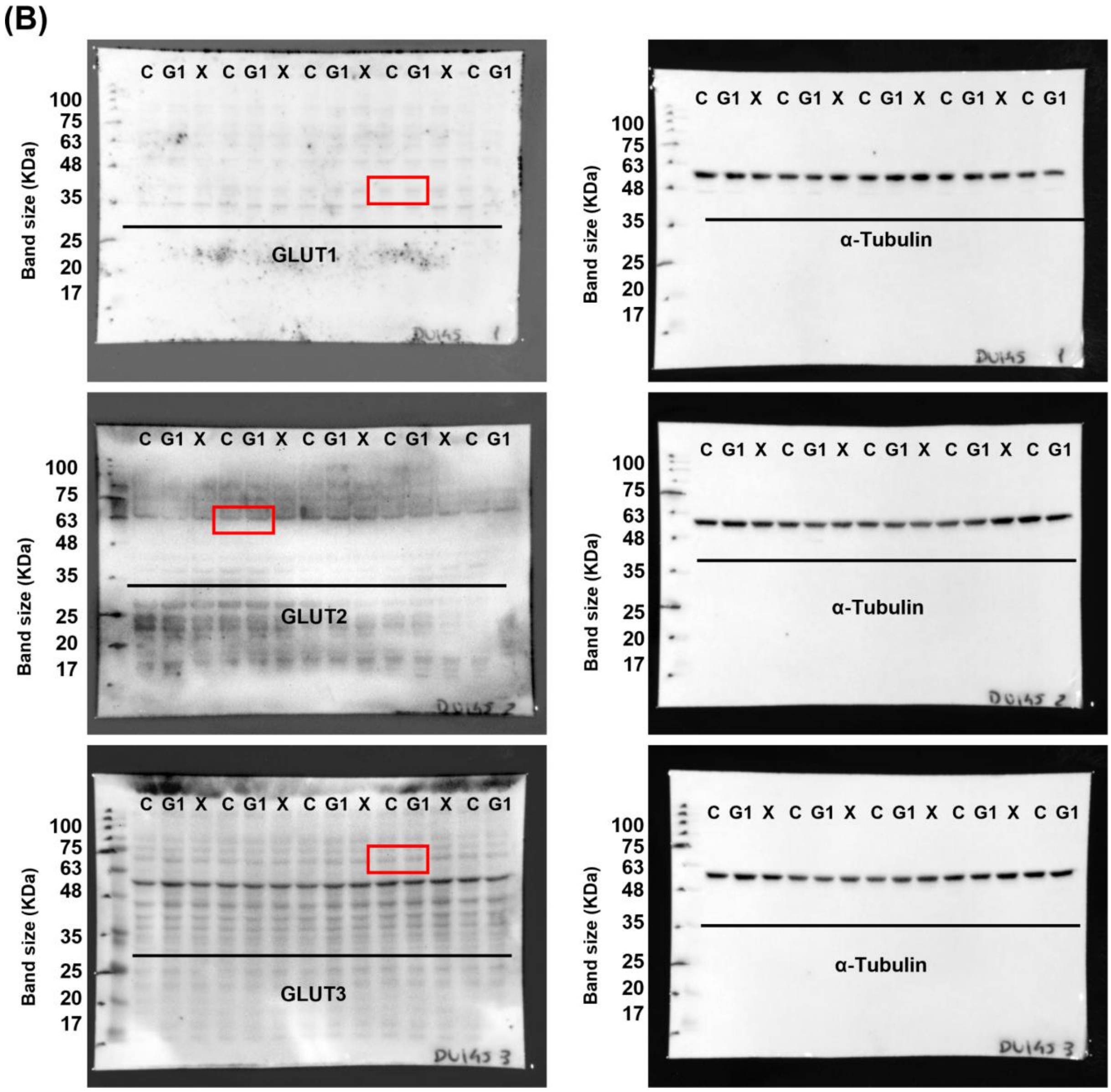
(B)
C G1 X C G1 X C G1 X C G1 X C G1
100
75
63
48
35
25
20
17
Band size (KDa)
GLUT1
α-Tubulin
GLUT2
GLUT3
DU145 1
DU145 2
DU145 3

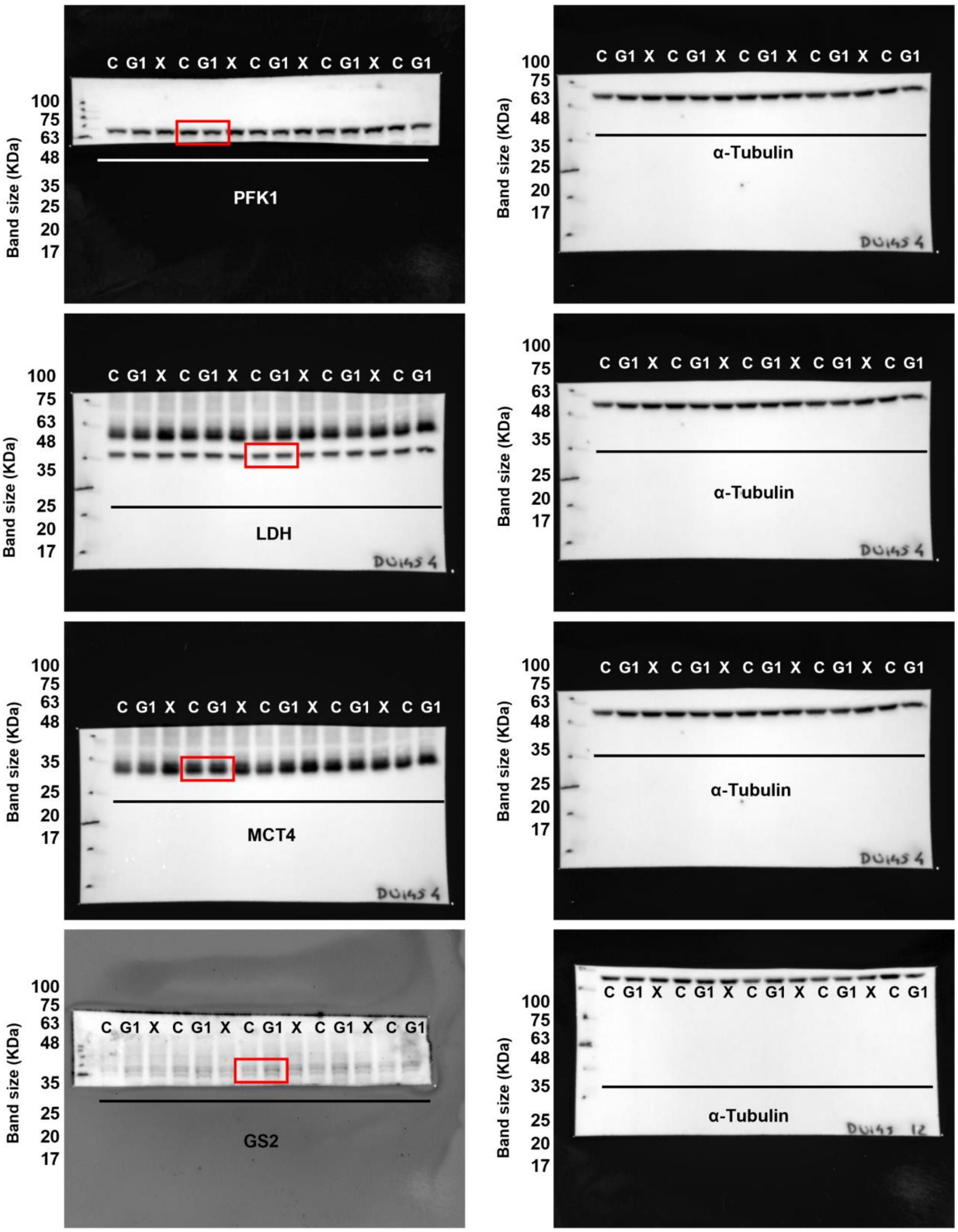
C G1 X C G1 X C G1 X C G1 X C G1
100
75
63
48
35
25
20
17
Band size (KDa)
PFK1
LDH
MCT4
GS2
α-Tubulin
α-Tubulin
α-Tubulin
α-Tubulin

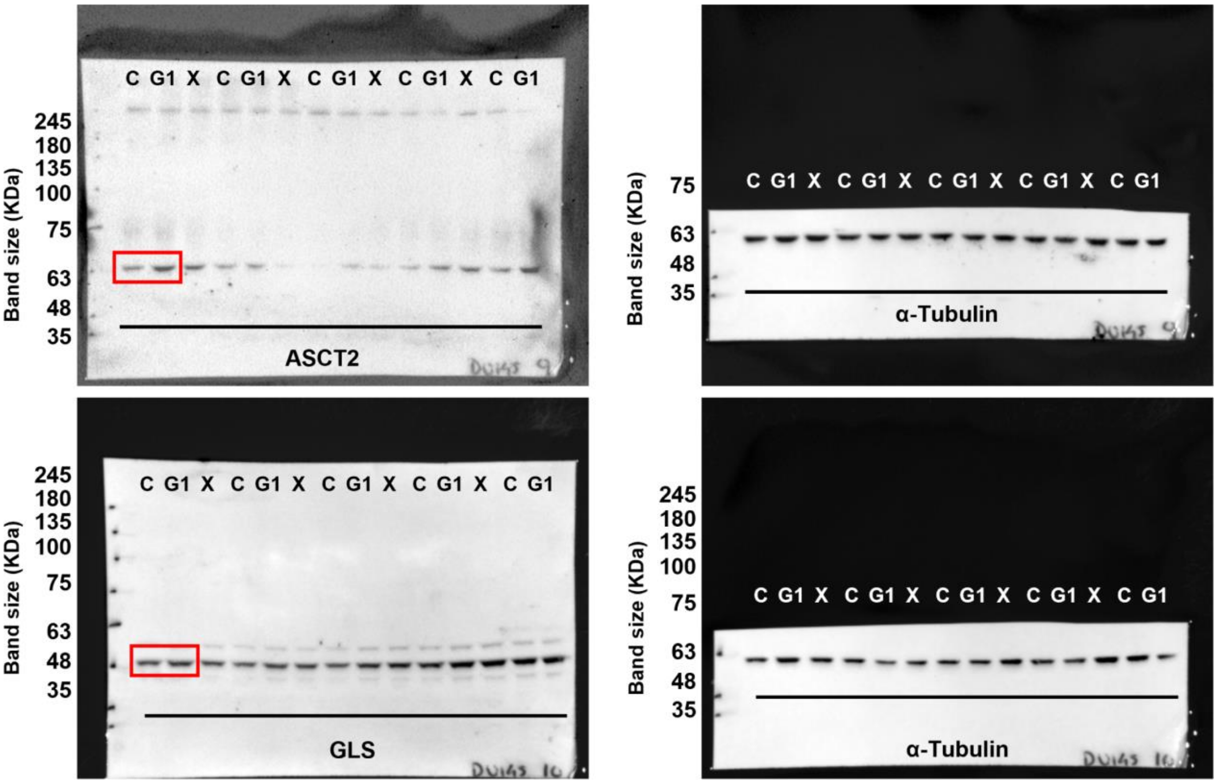
C G1 X C G1 X C G1 X C G1 X C G1
Band size (KDa)
245
180
135
100
75
63
48
35
ASCT2
α-Tubulin
GLS
α-Tubulin

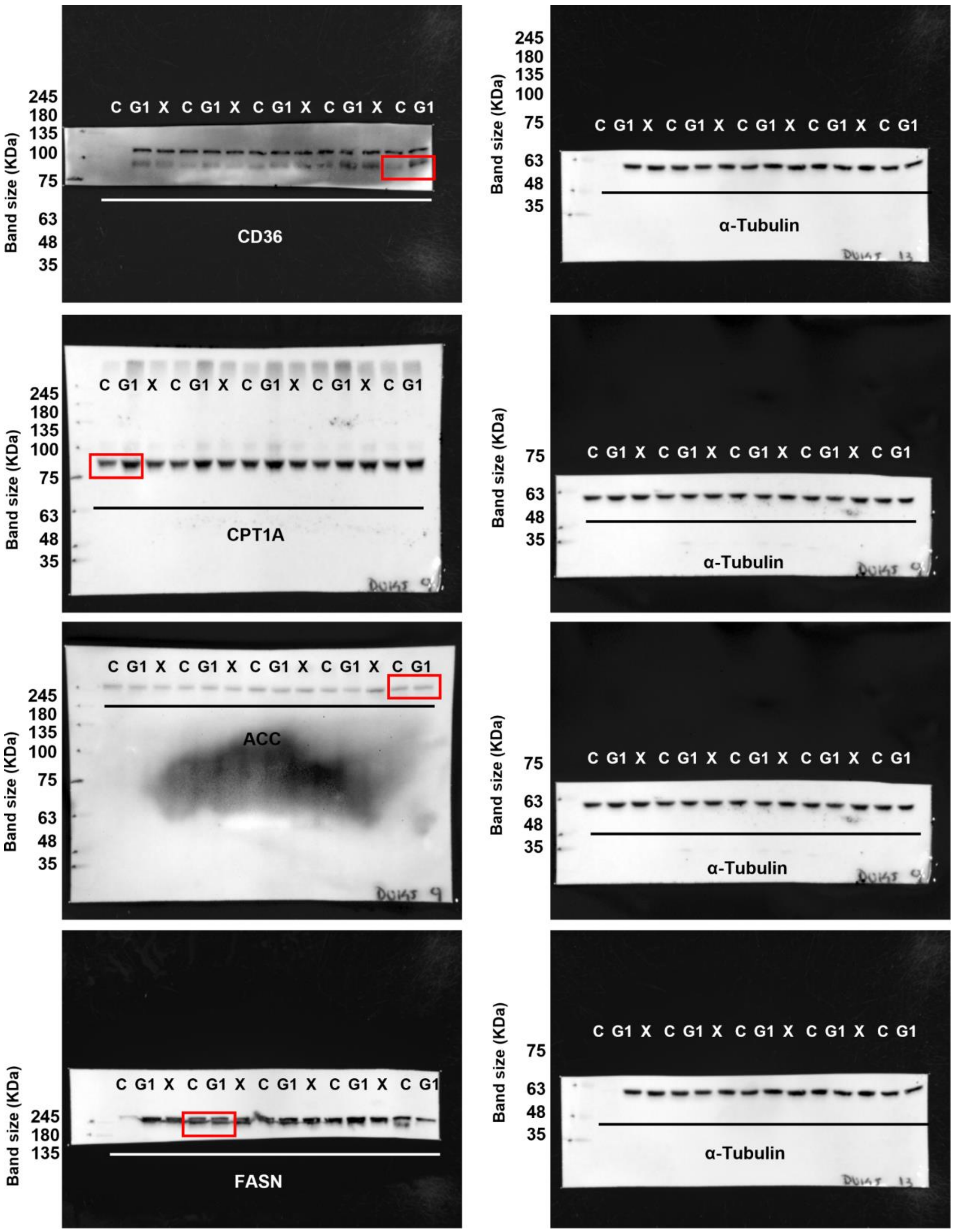
CD36
α-Tubulin
CPT1A
α-Tubulin
ACC
α-Tubulin
FASN
α-Tubulin
C G1 X C G1 X C G1 X C G1 X C G1
Band size (KDa)
245
180
135
100
75
63
48
35

(C)

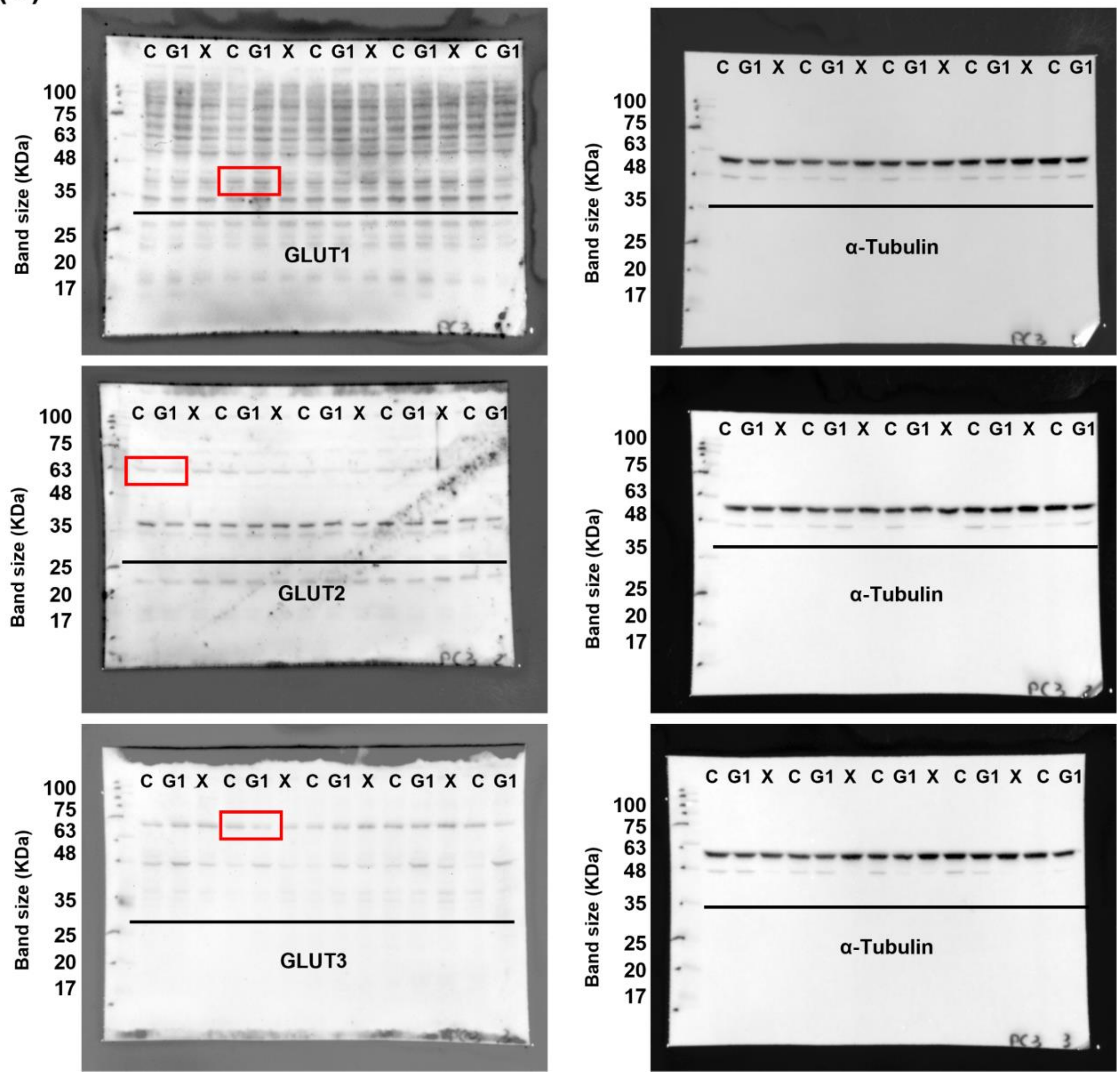
C G1 X C G1 X C G1 X C G1 X C G1
100
75
63
48
35
25
20
17
Band size (KDa)
GLUT1
α-Tubulin
GLUT2
GLUT3

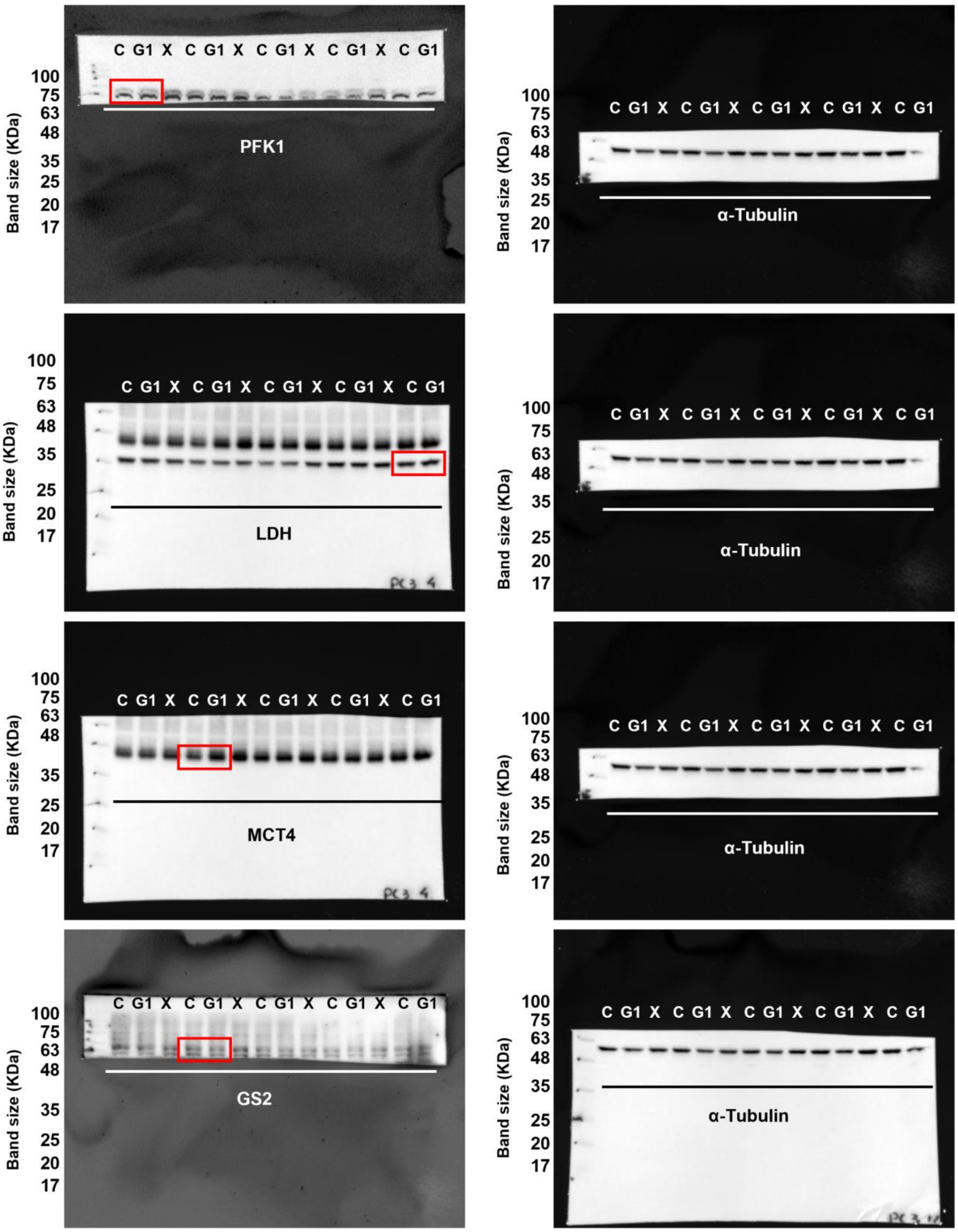
C G1 X C G1 X C G1 X C G1 X C G1
Band size (KDa)
100
75
63
48
35
25
20
17
PFK1
α-Tubulin
LDH
α-Tubulin
MCT4
α-Tubulin
GS2
α-Tubulin

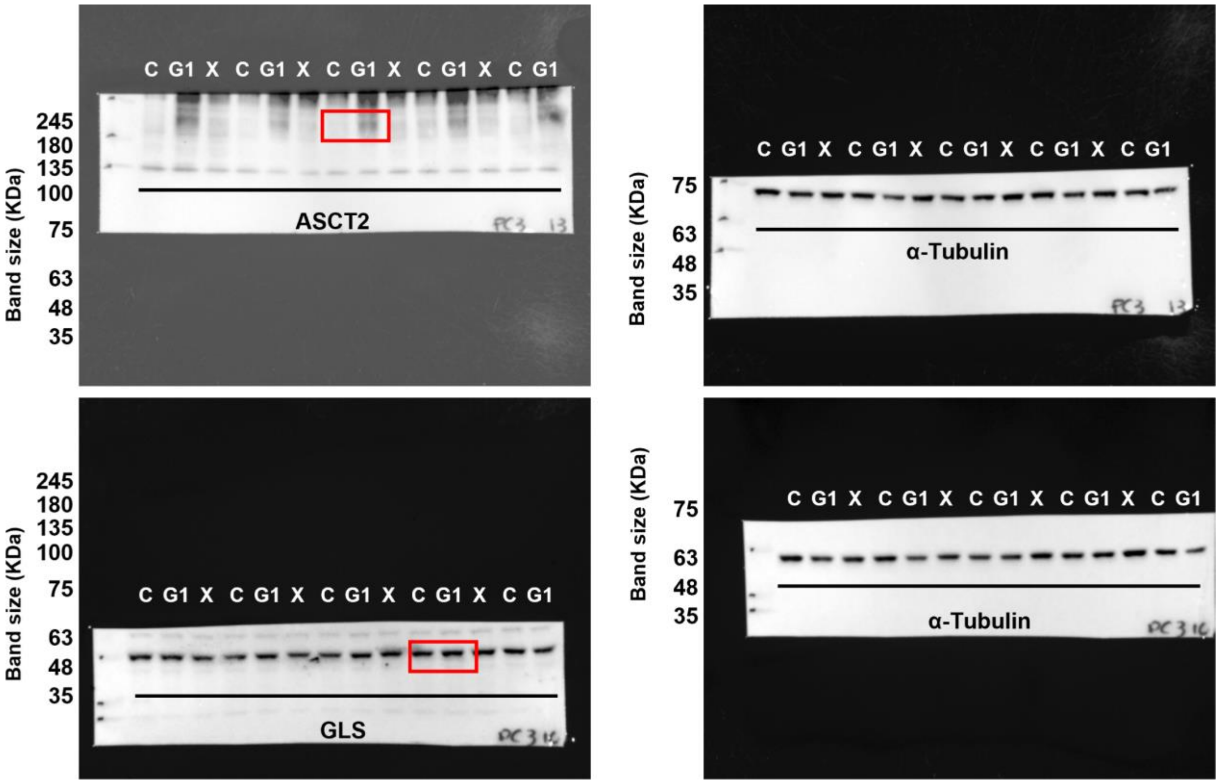
C G1 X C G1 X C G1 X C G1 X C G1
245
180
135
100
75
63
48
35
Band size (KDa)
ASCT2
α-Tubulin
GLS

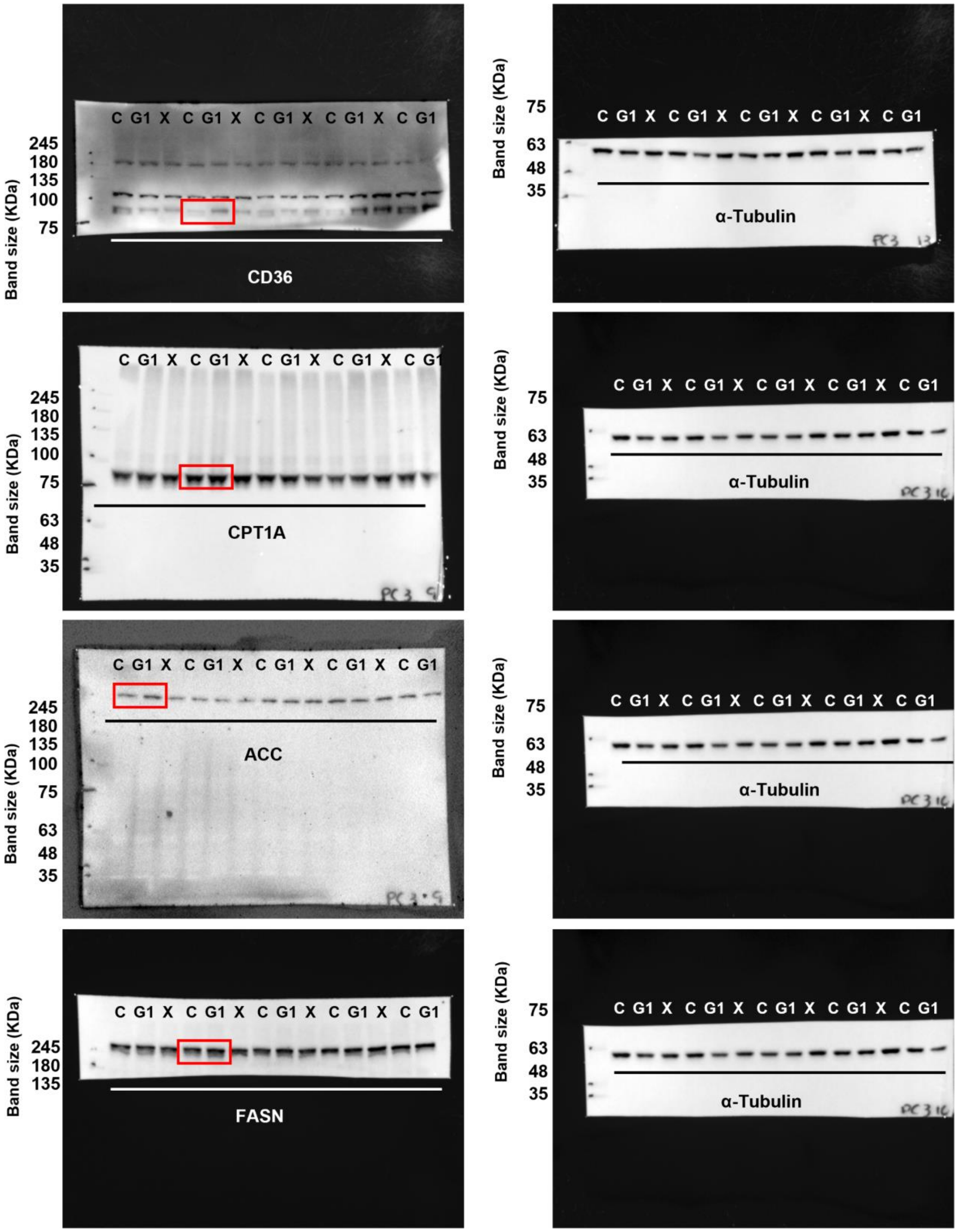

Figure S6. Original Western blots images after GLUT1, GLUT2, GLUT3, PFK1, LDH, MCT4, GS2, ASCT2, GLS, CD36, CPT1A, ACC, FASN and α-tubulin immunodetection in (A) LNCaP, (B) DU145 and (C) PC3 cells protein extracts after treatment with G1 for 24 h. Legend: C – control group; G1- G1-treated group; X – other samples analyzed. Red boxes represent the E, F and G pairs selected as representative to be shown in Figure 11.

Methodological notes:

Due to the presence of unspecific staining, to acquire the immunoblot images, some membranes were cut. Moreover, to remove the unspecific bands around 56 KDa, a soft stripping protocol was performed after GLUT1, GLUT2, GLUT3, MCT4, GS2, ASCT2 and GLS detection and before incubating the membrane with the anti-α-tubulin antibody.

All the proteins blots represented were grouped by cell line and only one membrane of α-tubulin was shown in the manuscript.

https://doi.org/10.3390/xxxxx
