# Supplementary material for: G Protein-Coupled Oestrogen Receptor Actions Targeting the Hallmarks of Cancer in Human Prostate Cells: From Cell Fate to Metabolic Reprogramming
Source: Cancers (Basel). 2026 Apr 1;18(7):1137. doi: 10.3390/cancers18071137 (PMC13072317; doi:10.3390/cancers18071137)
Supplement: Supplementary file 1 [file cancers-18-01137-s001.zip › cancers-4157962-supplementary.pdf]

Supplementary Table S1. Primary antibodies and working dilutions used in the WB analysis

Biological target/process	Antibody	Host species	Dilution	Catalogue number	Supplier
Hormone receptors	Anti-GPER	Rabbit	1:500	(K-19)-R: sc-48524-R	SCBT
	Anti-GPER	Rabbit	1:250	ab 39742	Abcam, Cambridge, UK
	Anti-Androgen receptor	Rabbit	1:1000	N-20: sc-816	SCBT
	Anti-Androgen receptor	Rabbit	1:1000	C-19: sc-815	SCBT
Cell cycle	Anti-Stem Cell Factor (SCF)	Rabbit	1:250	H-189: sc-9132	SCBT
	Anti-c-KIT	Rabbit	1:250	C-19: sc-168	SCBT
	Anti-Phospho-c-Myc (Ser62) (E1J4K)	Rabbit	1:1000	#13748	Cell Signaling Technology, Danvers, MA, USA
	Anti-p53	Rabbit	1:1000	FL-393: sc-6243	SCBT
	Anti-p21	Rabbit	1:1000	C-19: sc-397	SCBT
Intracellular signaling	Anti-AKT	Rabbit	1:500	# 9272	Cell Signaling
	Anti-Phospho-AKT (Ser473)	Rabbit	1:500	# 9271	Cell Signaling
	Anti-p44/42 MAPK (Erk1/2)	Rabbit	1:1000	# 9102	Cell Signaling
	Anti-Phospho-p44/42 MAPK (Erk1/2) (Thr202/Tyr204)	Rabbit	1:500	# 9101	Cell Signaling
Apoptosis	Anti-Bax	Rabbit	1:1000	# 2772	Cell Signaling
	Anti-Bcl-2	Rabbit	1:1000	# 2876	Cell Signaling
	Anti-Caspase-9 p35	Rabbit	1:1000	H-170:sc-8355	SCBT
	Anti-Caspase-8 p18	Mouse	1:100	D-8: sc-5263	SCBT
	Anti-FAS	Rabbit	1:1000	A-20: sc-1023	SCBT
	Anti-FAS-L	Rabbit	1:1000	C-178: sc-6237	SCBT
Metabolism	Anti-Glucose Transporter GLUT1	Rabbit	1:1000	CBL 242	Millipore, Darmstadt, Germany
	Anti-Glucose Transporter GLUT2	Rabbit	1:1000	H-67: sc-9117	SCBT
	Anti-Glucose Transporter GLUT3	Rabbit	1:1000	H-50: sc-30107	SCBT

	Anti-Phosphofructokinase 1 (PFK1)	Rabbit	1:1000	H-55: sc-67028	SCBT
	Anti-Lactate Dehydrogenase (LDH) [EP1566Y]	Rabbit	1:10000	ab 52488	Abcam
	Anti-Monocarboxylate Transporter 4 (MCT4)	Rabbit	1:1000	H-90: sc-50329	SCBT
	Anti-Glycogen Synthase 2 (GS2)	Mouse	1:200	G-8: sc-390391	SCBT
	Anti-Alanine, Serine, Cysteine Transporter 2 (ASCT2) (V501)	Rabbit	1:500	# 5345	Cell Signaling
	Anti-Glutaminase (GLS)	Rabbit	1:1000	ab 93434	Abcam
	Anti-Cluster of Differentiation 36 (CD36)	Rabbit	1:400	ab 64014	Abcam
	Anti-Carnitine Palmitoyltransferase 1A (CPT1A) [8F6AE9]	Mouse	1:1000	ab 128568	Abcam
	Anti-Acetyl CoA Carboxylase (ACC)	Rabbit	1:1000	# 3662	Cell Signaling
	Anti-Fatty Acid Synthase (FASN) (C20G5)	Rabbit	1:1000	# 3180	Cell Signaling
**EMT**	Anti-E-cadherin	Mouse	1:100	G-10: sc-8426	SCBT
	Anti-N-cadherin	Mouse	1:500	13A9: sc-59987	SCBT
	Anti-Vimentin	Mouse	1:1000	V9: sc-6260	SCBT
	Anti-Cytokeratin-8	Rabbit	1:1000	ab 59400	Abcam
	Anti-Cytokeratin-18 [E431-1]	Rabbit	1:10000	ab 32118	Abcam
**Housekeeping genes**	Anti-α-Tubulin	Mouse	1:10000	T9026	Sigma-Aldrich
	Anti-β-Actin	Mouse	1:10000	A1978	Sigma-Aldrich
